# Achmatowicz rearrangement-enabled unified total syntheses of (+)-passifetilactones A–C

**DOI:** 10.1039/d5ra06982c

**Published:** 2025-10-21

**Authors:** Aman Kumar Verma, Dharmaraju Jeddi, Ravindar Kontham

**Affiliations:** a Organic Chemistry Division, CSIR-National Chemical Laboratory Dr Homi Bhabha Road Pune-411008 India k.ravindar@ncl.res.in; b Academy of Scientific and Innovative Research (AcSIR) Ghaziabad-201002 India

## Abstract

In this manuscript, we report the enantio- and diastereoselective total synthesis of three cytotoxic 2-pyrone-derived natural products passifetilactones A–C. Our strategy leverages a unified synthetic approach that originates from simple furan-based building blocks. Key transformations include the Corey–Bakshi–Shibata (CBS) reduction to access chiral furan-derived alcohol, NBS-mediated Achmatowicz rearrangement to construct the α-hydroxy–*δ*-pyrone core, followed by a highly stereoselective, iridium-catalyzed dynamic kinetic intramolecular redox isomerization to access the *δ*-hydroxy-α-pyrone framework. This streamlined route enables efficient access to passifetilactones A, B, and C in 13, 5, and 8 steps, with overall yields of 12%, 54%, and 37%, respectively.

## Abstract 2

In this manuscript, we report the enantio- and diastereoselective total synthesis of three cytotoxic 2-pyrone-derived natural products passifetilactones A–C.

## Graphical abstract

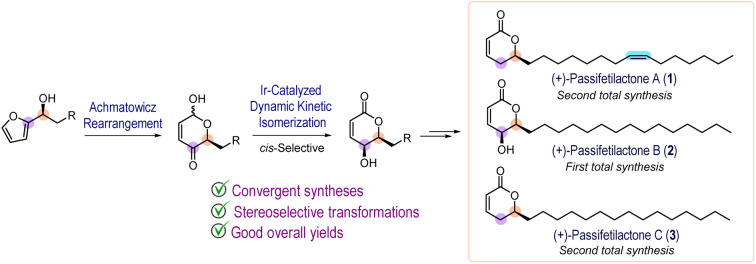

## Introduction

Nature is a prolific source of diverse heterocyclic compounds essential to countless biochemical processes and known for their valuable biological activities. Studying these natural products is a cornerstone of medicinal chemistry and a key driver in drug discovery.^1^ Indeed, most drugs developed over the past five decades are either natural products or inspired by them. Among these, α-pyrone, an unsaturated six-membered lactone is a prominent natural heterocycle found across all life forms, including bacteria, fungi, marine organisms, plants, and animals. In nature, α-pyrones serve as metabolic intermediates, signaling molecules, and defense agents. Notably, they exhibit a wide range of biologically significant activities with high therapeutic potential.^2^

**Fig. 1:**
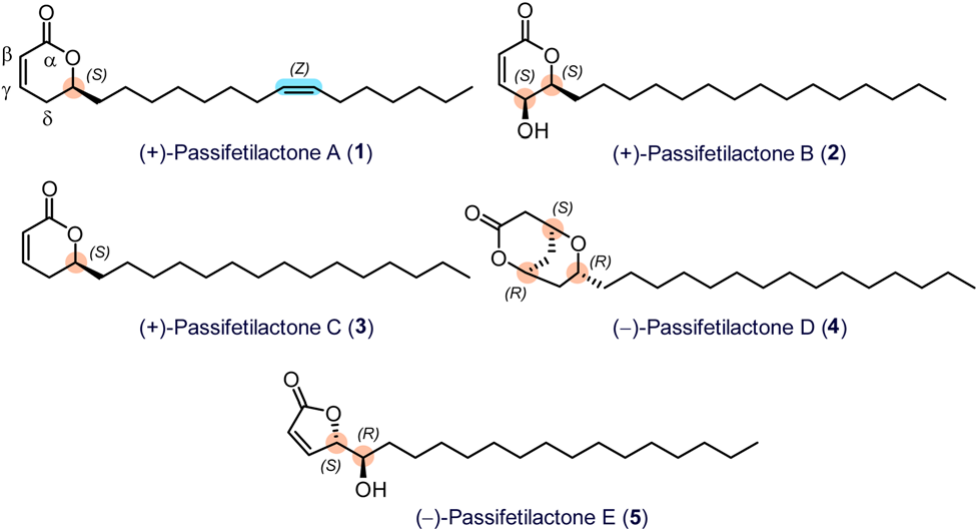
Reported chemical structures of natural passifetilactones A–E (1–5).

In 2024, Schevenels' research group in Thailand reported the isolation, structural characterization, and cytotoxicity assessment of a new series of fatty acid lactones, named passifetilactones A–E (1–5), from previously unexplored fruits and flowers of *Passiflora foetida* (the plant is utilized in traditional medicine across its entire geographic range).^3^ The absolute stereochemistry of these compounds was determined through comprehensive NMR spectroscopy (1D and 2D) combined with a comparison of experimentally obtained and calculated electronic circular dichroism (ECD) spectra, the latter generated using time-dependent density functional theory (TDDFT). These newly identified lactones were evaluated for their cytotoxic effects on a panel of cancer cell lines, including HeLa, A549, PC-3, KKU-055, and KKU-213A, as well as two non-cancerous cell lines, Vero and MMNK-1. Passifetilactones B (2) and C (3) exhibited moderate to notable cytotoxicity, with IC_50_ values ranging from 3.7 to 25.9 μM and 12.2 to 19.8 μM, respectively, across six cell lines. However, both compounds demonstrated only limited activity against the MMNK-1 line. Furthermore, flow cytometry analysis revealed that passifetilactones B and C (2 and 3) triggered apoptotic cell death in the KKU-055 cancer cell line (Fig. 1).^3^

**Scheme 1:**
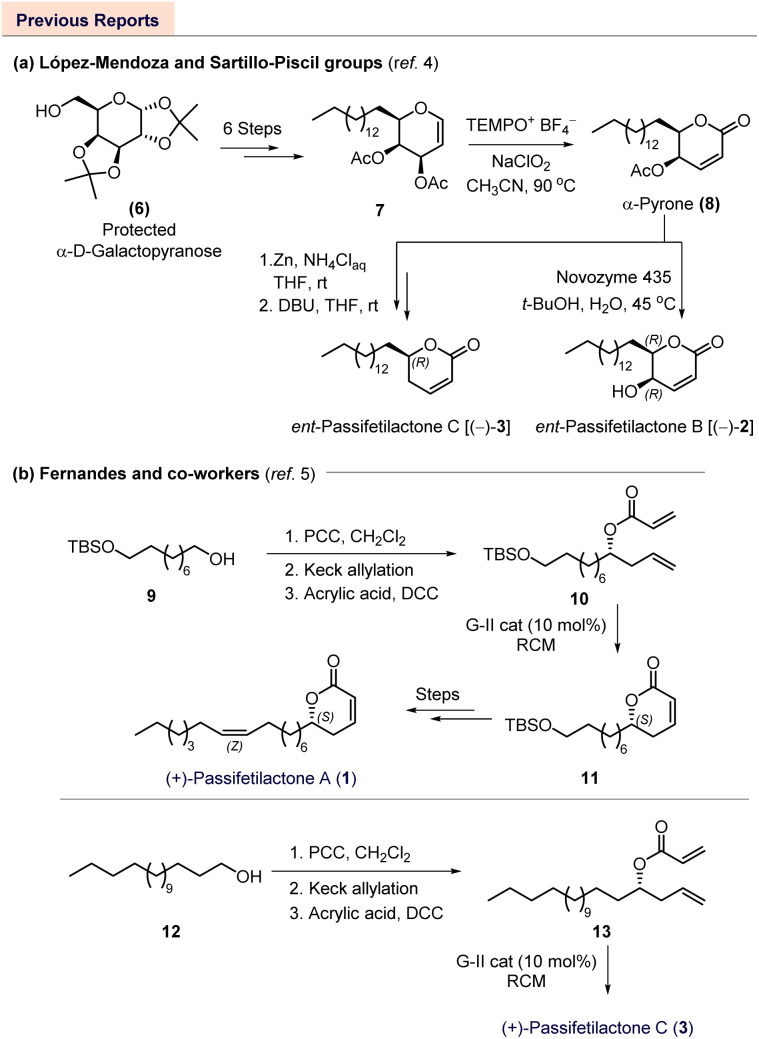
Previous reports on the synthesis of (−)-passifetilactone B and C (enantiomers of isolated natural products), and (+)-passifetilactone A and C (isolated natural products).

In the course of our ongoing research, López-Mendoza and Sartillo-Piscil reported an elegant synthetic strategy for the putative enantiomers of passifetilactones B [(−)-2] and C [(−)-3]. Their work was part of a broader study on the TEMPO-cation- and NaClO_2_-mediated oxa-Ferrier rearrangement of glycals to access chiral α,β-unsaturated *δ*-lactones (entry a, Scheme 1).^4^

More recently, Fernandes and co-workers developed an efficient synthetic route to passifetilactones A (1), C (3), E (5), and 4-*epi*-passifetilactone B (*epi*-2), utilizing Keck allylation, Sharpless kinetic resolution (SKR), and ring-closing metathesis (RCM) as key steps (entry b, Scheme 1).^5^

Inspired by the intriguing biological activities and structural features of pyrone-derived natural products, and as part of our ongoing efforts in the stereoselective total synthesis of natural products,^6^ we embarked on the development of a unified stereoselective synthetic route to passifetilactones A–C (1–3).

## Results and discussion

**Scheme 2:**
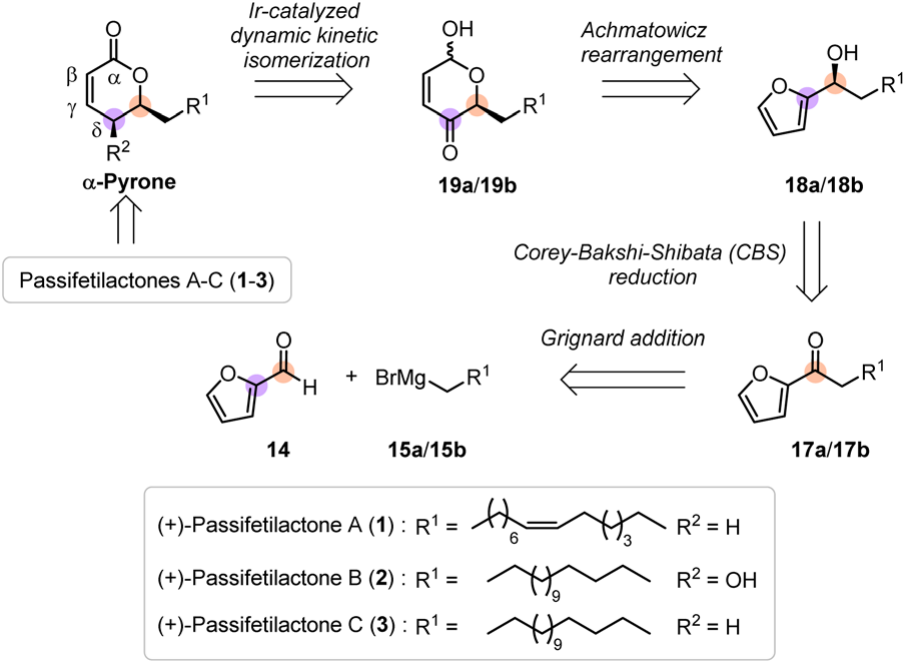
Retrosynthetic analysis of passifetilactones A-C (1–3).

In our initial retrosynthetic analysis, we envisioned a concise, protecting-group-free synthetic route to passifetilactones A–C (1–3), which feature *distinct* aliphatic C15 side chains appended to a common pyrone core. These natural products differ in the presence or absence of a hydroxyl group at the *δ*-position of the pyrone ring and in a *cis* double bond within the side chain (Scheme 2).

We proposed accessing these targets 1–3 from a suitably functionalized α-pyrone intermediate, prepared *via* an iridium-catalyzed dynamic kinetic intramolecular redox isomerization (*cis*-selective) of chiral lactol precursors 19a/19b (α-hydroxy-*δ*-pyrones). These lactols, in turn, could be efficiently generated through an NBS-mediated Achmatowicz rearrangement of chiral hydroxyalkyl-tethered furans 18a/18b. The furan precursors are readily synthesized from the inexpensive feedstock chemical furfural (14) *via* Grignard addition of appropriately selected organometallic reagents 15a/26a, followed by oxidation to yield furyl ketones 17a/17b, and subsequent enantioselective [(*S*)-selective] Corey–Bakshi–Shibata (CBS) reduction (Scheme 2).

**Scheme 3:**
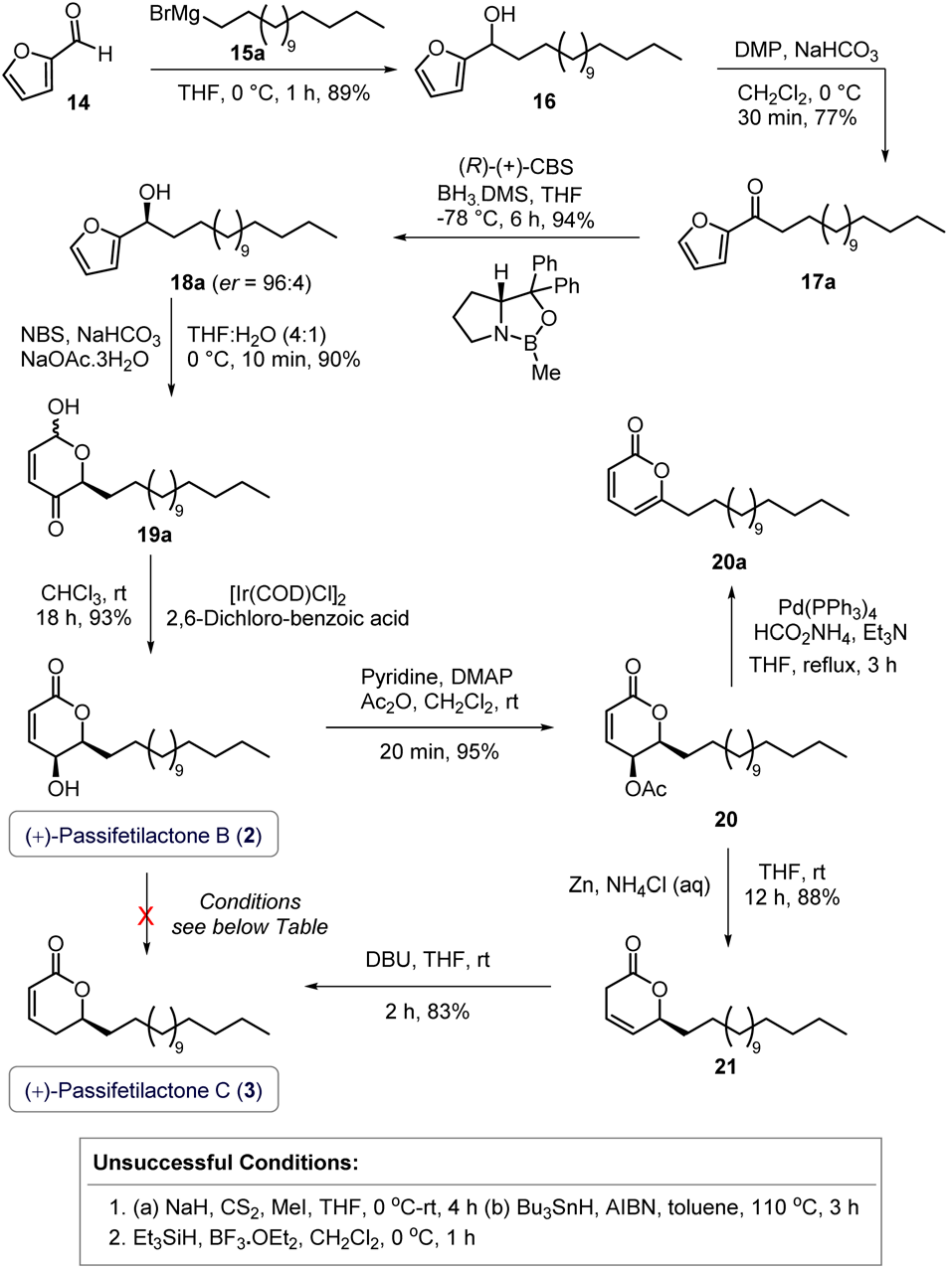
Total synthesis of (+)-passifetilactone B (2) and (+)-passifetilactone C (3).

Hence, our efforts were primarily directed toward developing a synthetic route to passifetilactone B (2), which can serve as a precursor for the synthesis of its *δ*-dehydroxylated analog, passifetilactone C (3) (Scheme 3). Starting from commercially available furfural (14), a Grignard reaction with freshly prepared pentadecylmagnesium bromide (15a) in THF afforded the secondary alcohol 16 in 89% yield. Oxidation of 16 using Dess–Martin periodinane (DMP)^7^ provided the corresponding ketone intermediate 17a. The desired chirality [(*S*)] was then introduced *via* CBS reduction^8^ of ketone 17a using (*R*)-(+)-CBS catalyst, furnishing alcohol 18a with an enantiomeric ratio (er) of 96 : 4, as determined by chiral HPLC.^9^ With sufficient quantities of (*S*)-furyl alcohol 18a in hand, we proceeded with an NBS-mediated Achmatowicz rearrangement,^10,11^ which cleanly delivered the α-hydroxy-*δ*-pyrone 19a in 90% isolated yield (dr = 3 : 1). Subsequently, an iridium-catalyzed dynamic kinetic intramolecular redox isomerization, developed by Guo and Tang,^12^ was performed on 19a, efficiently yielding (+)-passifetilactone B (2) in five linear steps with an overall yield of 54% (first total synthesis) and exclusive substrate-controlled diastereoselectivity. The spectroscopic and spectrometric data of 2 were consistent with the reported values.^3,5^ The observed optical rotation {this work: [*α*]^25^_D_ = +14.6, *c* = 2.0, in MeOH; lit (isolation):^3^: [*α*]^21^_D_ = +8.0 (*c* = 0.1, MeOH)}, matched the reported sign, though with a slightly higher magnitude (Scheme 3).

We then turned our attention to the synthesis of (+)-passifetilactone C (3) from passifetilactone B (2) *via* reductive dehydroxylation. Initial strategies involving Barton–McCombie deoxygenation^13^ and BF_3_·Et_2_O-mediated Et_3_SiH reduction of 2 proved ineffective.^14^ Pd(PPh_3_)_4_-catalyzed reductive dehydroxylation^15^ of acetate 20 (obtained from 2, through acetylation) afforded the undesired doubly conjugated pyrone 20a.^9^ Hence, we followed the sequence reported by López-Mendoza and Sartillo-Piscil (during their synthesis of unnatural (−)- isomer of passifetilactone C)^4^ in which the free hydroxyl group of compound 2 was converted into its corresponding acetate 20. A subsequent zinc-mediated elimination furnished the double bond–isomerized intermediate 21. Finally, DBU-mediated double bond *trans* position of compound 21 smoothly led to the formal total synthesis of passifetilactone C (3) with overall yield of 37% (Scheme 3).^4^ Spectroscopic and spectrometric data of 3 was in agreement with the literature. The observed optical rotation {this work: [*α*]^25^_D_ = +18.4, *c* = 0.5, in CHCl_3_; lit (isolation^3^ and first synthesis):^5^: [*α*]^21^_D_ = +2.0 (*c* = 0.1, MeOH); lit (first synthesis):^5^: [*α*]^21^_D_ = +2.4 (*c* = 0.1, MeOH)}, matched the reported sign, though with a slightly higher magnitude (Scheme 3).

**Scheme 4:**
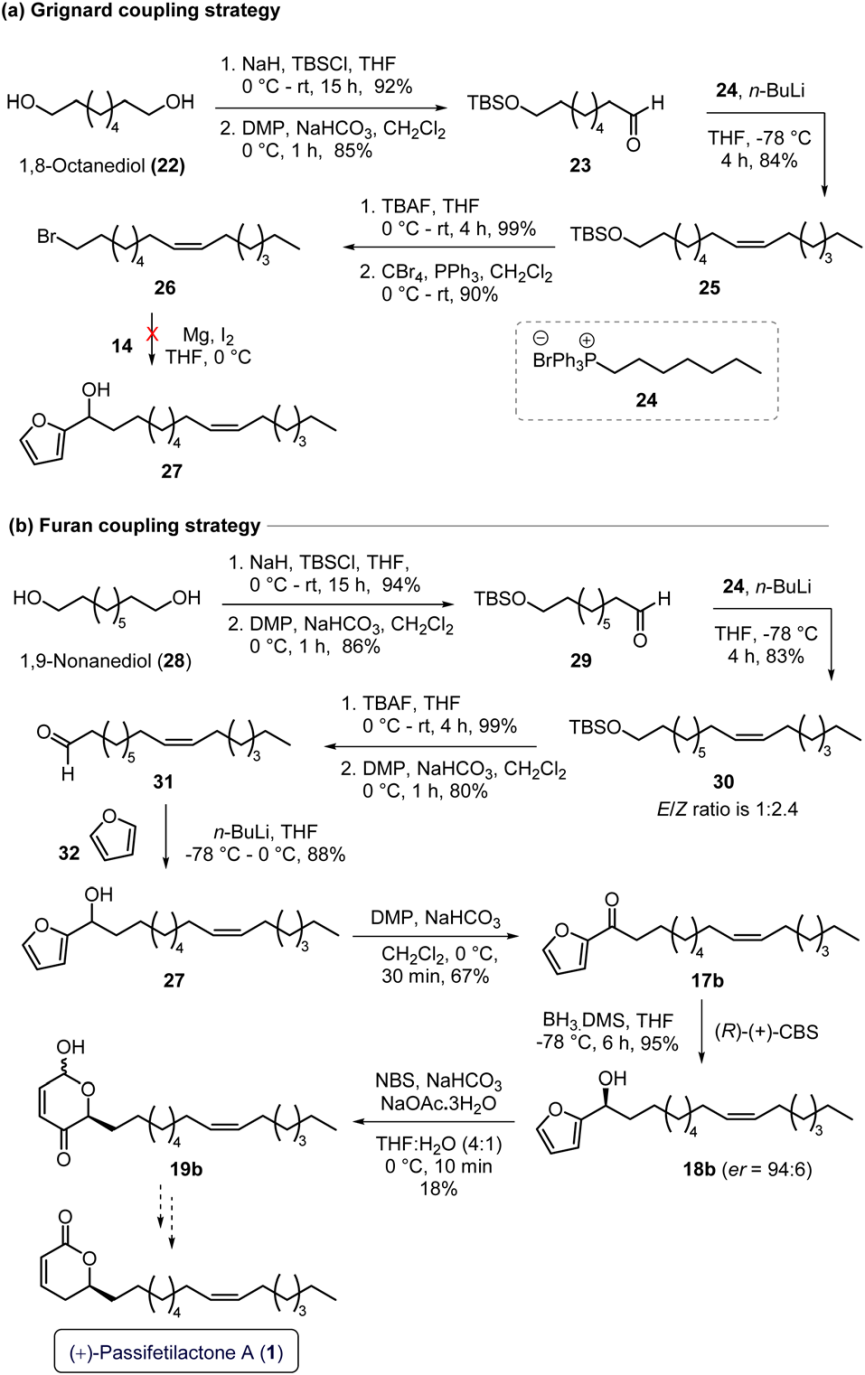
Studies directed towards the synthesis of (+)-passifetilactone A (1).

Having successfully accomplished the total synthesis of (+)-passifetilactones B (2) and C (3), we next set out to synthesize (+)-passifetilactone A (1), which features a *Z*-olefin moiety in its alkyl side chain (Scheme 4). Initially, we employed a strategy similar to that used for the synthesis of 2 and 3, relying on alkenyl bromide 26 and furfural (14) building blocks (entry a, Scheme 4).

To this end, the *Z*-olefin-containing bromide intermediate 26 was synthesized from 1,8-octanediol (22) *via* monoprotection with TBSCl, followed by Dess–Martin periodinane (DMP) oxidation to furnish aldehyde 23. A subsequent Wittig olefination with freshly prepared phosphonium salt 24 provided the alkenyl-tethered TBS ether 25. Deprotection of the TBS group followed by treatment with CBr_4_ and triphenylphosphine (TPP) furnished the desired alkenyl bromide 26. However, several attempts at Grignard addition to furfural (14) were unsuccessful, prompting us to revise our approach and avoid the Grignard step altogether (entry a, Scheme 4).

For this revised strategy, we selected 1,9-nonanediol (28) as the precursor, which was converted into the alkenyl-tethered TBS ether 30 using a synthetic sequence analogous to that employed for intermediate 25 (entry a, Scheme 4), involving TBS protection, oxidation, and Wittig olefination (28 → 29 → 30). Subsequent deprotection of the TBS group of 30 using TBAF in THF, followed by DMP oxidation, furnished the desired aldehyde fragment 31. This aldehyde 31 was then coupled with furan (32) to afford the secondary alcohol 27 in 88% yield. The racemic alcohol 27 was transformed into the chiral alcohol (*S*)-18b (er = 94 : 6) *via* DMP oxidation to ketone 17b, followed by CBS reduction (entry b, Scheme 4).

Next, we attempted the key NBS-mediated Achmatowicz rearrangement on intermediate 18b. Unfortunately, the reaction delivered the desired product 19b in only 18% isolated yield, with significant decomposition observed across multiple trials. The low efficiency is likely due to the sensitivity of the *Z*-olefin moiety in the substrate 18b under the conditions employed (entry b, Scheme 4).

**Scheme 5:**
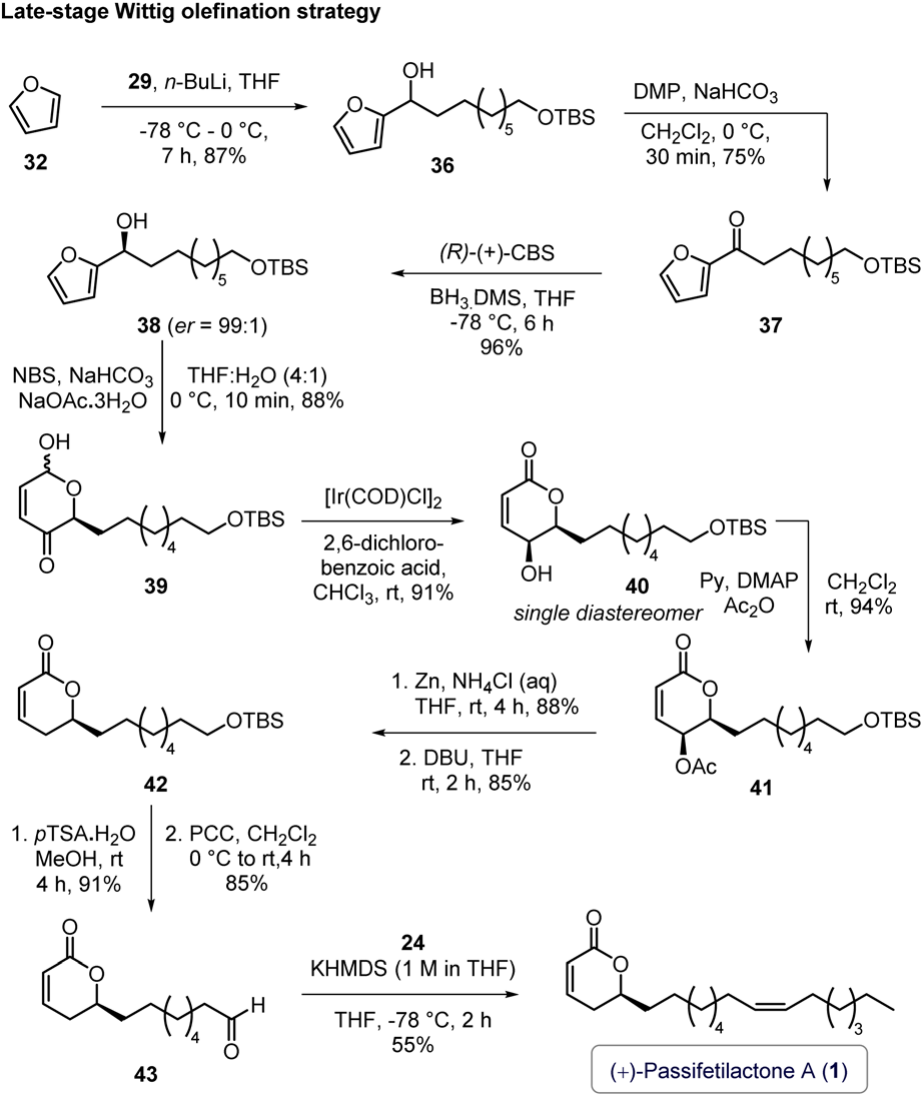
Completion of the total synthesis of (+)-passifetilactone A (1).

To address these challenges, we slightly modified the synthetic sequence by performing the NBS-mediated Achmatowicz rearrangement prior to the Wittig olefination (Scheme 5). Accordingly, aldehyde 29 (obtained from 1,9-nonanediol; see entry b, Scheme 4) was coupled with furan (32) to afford alcohol 36, which was then subjected to DMP oxidation (to give 37), followed by CBS reduction to yield the hydroxyl-alkyl-tethered furan (*S*)-38 (Scheme 5).

Pleasingly, the NBS-mediated Achmatowicz rearrangement of 38 proceeded smoothly, delivering the α-hydroxy-*δ*-pyrone 39 in 88% yield. Subsequent Ir-catalyzed dynamic kinetic isomerization of 39 furnished compound 40 in good yield. This was then subjected to a sequential transformations (as used for (+)-passifetilactone C in Scheme 3) involving O-acylation, Zn-mediated elimination, and DBU-assisted double bond *trans* position (40 → 41 → 42) to afford pyrone 42. Deprotection of the TBS group in 42, followed by PCC oxidation, provided the aldehyde intermediate 43. Finally, KHMDS-mediated Wittig olefination of 43 using phosphonium salt 24 delivered the target compound, passifetilactone A (1), in 55% yield (12% overall yield). ^1^H, ^13^C NMR, and HRMS data of 1 is in full agreement with the literature. Whereas, the optical rotation {this work: [*α*]^25^_D_ = +0.3 (*c* = 0.7, MeOH); lit (isolation):^3^: [*α*]^21^_D_ = +6.0 (*c* = 0.1, MeOH); lit (first synthesis):^5^: [*α*]^21^_D_ = +6.3 (*c* = 0.1, MeOH)}, data was found to be low in magnitude, and with same sign (Scheme 5).^9^

## Conclusions

In summary, we have accomplished the total syntheses of passifetilactones A, B, and C in 13, 5, and 8 steps, with overall yields of 12%, 54%, and 37%, respectively. This unified synthetic strategy leverages high-yielding and operationally simple transformations, including a key Corey–Bakshi–Shibata (CBS) reduction to establish the chiral furan-derived alcohol, an NBS-mediated Achmatowicz rearrangement to construct the α-hydroxy-*δ*-pyrone framework, and a highly stereoselective, Ir-catalyzed dynamic kinetic intramolecular redox isomerization to access the *δ*-hydroxy-α-pyrone motif. This streamlined and modular approach not only provides efficient access to these natural products but also lays a foundation for future medicinal chemistry exploration of passifetilactones and related bioactive natural products.

## Experimental

### General information

All reactions were performed under an argon atmosphere and using an oven (80 °C) or flame-dried glassware with a septum seal or sealed tubes. Tetrahydrofuran (THF) was distilled from sodium benzophenone under an argon atmosphere immediately before use. Anhydrous methanol, chloroform, and dichloromethane were purchased from commercial sources. Reaction temperatures are reported as the oil bath temperature surrounding the reaction vessel. Analytical thin layer chromatography (TLC) was performed on TLC Silica gel 60 F254. Visualization was accomplished with shortwave UV light, anisaldehyde, or KMnO_4_ staining solutions, followed by heating. Chromatography was performed on silica gel (100–200 mesh) or using neutral aluminium oxide by standard techniques eluting with solvents as indicated. For NMR analysis, ^1^H and ^13^C NMR spectra were recorded on Bruker AV 400 and 500 in solvents as indicated. Chemical shifts (*δ*) are given in ppm. The residual solvent signals were used as references, and the chemical shifts were converted to the TMS scale (CDCl_3_: *δ*_H_ = 7.26 ppm, *δ*_C_ = 77.16 ppm). The following abbreviations were used: s, singlet; d, doublet; t, triplet; q, quartet; m, multiplet; dd, doublet of doublet; td, triplet of doublet; and br, broad. HRMS data were recorded on a Thermo Scientific QExactive, Accela 1250 pump. Chiral HPLC separations were achieved using an Agilent 1260 Infinity series normal phase HPLC unit and HP Chemstation software with Chiralpak Daicel columns (250 × 4.6 mm). Compounds that are not presented in the main text of the manuscript are numbered starting from S1.

### 1-(Furan-2-yl)hexadecan-1-ol (16)

In a 50 mL two-neck round-bottom flask, magnesium turnings (0.129 g, 20.8 mmol) were heated at 90 °C under an argon atmosphere in the presence of a crystal of iodine until purple fumes evolved. A solution of 1-bromopentadecane (1.94 g, 6.66 mmol) in anhydrous THF (10 mL) was then added, and the mixture was stirred at room temperature for 5 h to generate the corresponding Grignard reagent. This freshly prepared Grignard reagent 15 was added dropwise to a solution of furfural 14 (0.4 g, 4.1 mmol) in another flask in anhydrous THF (10 mL) at 0 °C. The reaction mixture was allowed to cool to room temperature and stirred for the next 1 h. After completion of the reaction, it was quenched with a saturated aqueous solution of NH_4_Cl. The organic layer was separated, and the aqueous layer was extracted with EtOAc (20 mL × 3), dried over Na_2_SO_4_, and filtered. The solvent was evaporated under reduced pressure, and the crude product was purified by column chromatography using neutral aluminium oxide to afford the desired product 16 as an amorphous white solid (1.13 g, 89%). TLC: *R*_f_ = 0.5 (SiO_2_, 20% EtOAc : hexane). IR (CHCl_3_) 3371, 2921, 2852, 1670, 1465, 1215, 1150, 1008, 733, 667 cm^−1^; ^1^H NMR (400 MHz, CDCl_3_) *δ* 7.37 (s, 1H), 6.33 (d, *J* = 5.13 Hz, 1H), 6.23 (d, *J* = 3.13 Hz, 1H), 4.67 (br, s, 1H), 1.86–1.83 (m, 2H), 1.45–1.38 (m, 1H), 1.32–1.24 (m, 25H), 0.90–0.86 (m, 3H); ^13^C{^1^H} NMR (101 MHz, CDCl_3_) *δ* 157.1, 142.0, 110.2, 105.9, 68.0, 35.7, 32.1, 29.84, 29.81, 29.8, 29.75, 29.72, 29.67, 29.58, 29.55, 29.51, 25.7, 22.8, 14.3; HRMS (ESI): *m*/*z* calcd for C_20_H_35_O_2_ [M − H]^−^ 307.2625, found 307.2632.

### 1-(Furan-2-yl)hexadecan-1-one (17a)

In a 50 mL two-neck round-bottom flask, furyl alcohol 16 (1 g, 3.24 mmol) in anhydrous CH_2_Cl_2_ (10 mL) was cooled at 0 °C. To it was added Dess–Martin periodinane (2.06 g, 4.86 mmol) portion-wise, followed by the addition of solid NaHCO_3_ (0.326 g, 3.88 mmol), and the resulting suspension was stirred at the same temperature for 30 minutes. After completion of the reaction, it was quenched with a 1 : 1 ratio of a saturated aqueous solution of NaHCO_3_ and Na_2_S_2_O_3_, and the aqueous layer was extracted with CH_2_Cl_2_ (20 mL × 3). Then, the combined organic layers were washed with brine, dried over Na_2_SO_4_, filtered, and the solvent was evaporated under reduced pressure. The resulting crude product was purified by silica gel column chromatography to afford the desired product 17a as an amorphous white solid (0.775 g, 77%). TLC: *R*_f_ = 0.6 (SiO_2_, 20% EtOAc : hexane). IR (CHCl_3_) 2921, 2852, 1711, 1681, 1569, 1467, 1261, 1011, 755, 721 cm^−1^; ^1^H NMR (400 MHz, CDCl_3_) *δ* 7.57–7.56 (m, 1H), 7.17 (d, *J* = 3.63 Hz, 1H), 6.52 (d, *J* = 5.25 Hz, 1H), 2.8 (d, *J* = 7.63 Hz, 2H), 1.71 (m, 2H), 1.36–1.25 (m, 24H), 0.89–0.86 (m, 3H); ^13^C{^1^H} NMR (101 MHz, CDCl_3_) *δ* 190.0, 153.0, 146.3, 116.9, 112.2, 38.7, 32.1, 29.83, 29.81, 29.79, 29.75, 29.74, 29.62, 29.54, 29.5, 29.49, 29.48, 24.5, 22.8, 14.3; HRMS (ESI): *m*/*z* calcd for C_20_H_35_O_2_ [M + H]^+^ 307.2639, found 307.2632.

### (*S*)-1-(Furan-2-yl)hexadecan-1-ol (18a)

In a 25 mL two-neck round-bottom flask, furyl ketone 17 (0.3 g, 0.978 mmol) in anhydrous THF (8 mL) was cooled to −78 °C under an argon atmosphere. To it was added a premixed solution of (*R*)-2-methyl-CBS-oxazaborolidine (0.342 mL, 0.342 mmol, 1 M in THF) and borane dimethylsulfide (0.665 mL, 1.33 mmol, 2 M in THF) and allowed to stir at the same temperature for 6 h. After completion, the reaction mixture was quenched by saturated aqueous solution of NH_4_Cl, and the aqueous layer was extracted with EtOAc (10 mL × 3), dried over Na_2_SO_4_, and filtered. The solvent was evaporated under reduced pressure, and the crude product was purified by column chromatography using neutral aluminium oxide to afford the desired product 18a as an amorphous white solid (0.282 g, 94% yield). TLC: *R*_f_ = 0.5 (SiO_2_, 20% EtOAc : hexane). [*α*]^25^_D_ = −63.0 (*c* = 1.0, MeOH). The enantiomeric purity was determined by HPLC (CHIRALCEL OD-H column, *n*-hexane : *i*-PrOH = 97 : 3, flow rate = 1 mL min^−1^, *λ* = 235 nm, *t*_major_ = 7.45 min, *t*_minor_ = 6.76 min), er = 96 : 4. IR (CHCl_3_) 3371, 2921, 2852, 1670, 1465, 1215, 1150, 1008, 733, 667 cm^−1^; ^1^H NMR (400 MHz, CDCl_3_) *δ* 7.39–7.34 (m, 1H), 6.33 (d, *J* = 5.0 Hz, 1H), 6.13 (d, *J* = 3.25 Hz, 1H), 4.67 (d, *J* = 4.13 Hz, 1H), 3.65 (t, *J* = 6.63 Hz, 1H), 1.94–1.82 (m, 2H), 1.58–1.54 (m, 1H), 1.3–1.25 (m, 25H), 0.90–0.86 (m, 3H); ^13^C{^1^H} NMR (101 MHz, CDCl_3_) *δ* 157.1, 142.0, 110.2, 105.9, 68.0, 63.2, 35.7, 33.0, 32.1, 29.84, 29.81, 29.8, 29.76, 29.73, 29.67, 29.59, 29.58, 29.55, 29.51, 25.9, 25.7, 22.8, 14.3; HRMS (ESI): *m*/*z* calcd for C_20_H_37_O_2_ [M + H]^+^ 325.2737, found 325.2732.

### (2*S*)-6-Hydroxy-2-pentadecyl-2*H*-pyran-3(6*H*)-one (19a)

In a 25 mL two-neck round-bottom flask, furyl alcohol 18a (0.19 g, 0.615 mmol) in a 4 : 1 ratio of THF/H_2_O (2 mL) was cooled to 0 °C. To the above solution, solid NaHCO_3_ (0.103 g, 1.23 mmol), NaOAc·3H_2_O (0.083 g, 0.615 mmol), and *N*-bromosuccinimide (0.109 g, 0.615 mmol) were added sequentially, and the reaction mixture was allowed to stir for 10 min. After completion of reaction, the reaction mixture was quenched by saturated aqueous solution of NaHCO_3_, and the aqueous layer was extracted with EtOAc (20 mL × 3), dried over Na_2_SO_4_, and filtered. The solvent was evaporated under reduced pressure, and the crude product was purified by silica gel column chromatography to afford the desired product 19a as an amorphous white solid (0.18 g, 90%). TLC: *R*_f_ = 0.4 (SiO_2_, 40% EtOAc : hexane). [*α*]^25^_D_ = −107.3 (*c* = 1.0, MeOH). (dr = 3 : 1). IR (CHCl_3_) 3387, 2922, 2853, 2359, 1706, 1463, 1410, 1376, 1250, 1219, 1087, 1033, 928, 772, 724 cm^−1^; ^1^H NMR (400 MHz, CDCl_3_) *δ* 6.91–6.87 (m, 1H), 6.15–6.09 (m, 1H), 5.65 (t, *J* = 4.88 Hz, 1H), 4.56–4.53 (m, 1H), 3.06–3.04 (m, 1H), 1.92 (dd, *J* = 4.13, 10.26 Hz, 1H), 1.44–1.38 (m, 2H), 1.29–1.25 (m, 25H), 0.87 (t, *J* = 6.88 Hz, 3H); ^13^C{^1^H} NMR (101 MHz, CDCl_3_) *δ* 196.8, 144.3, 127.9, 87.8, 74.4, 32.1, 30.8, 29.84, 29.8, 29.7, 29.65, 29.58, 29.55, 29.54, 29.51, 25.3, 25.1, 22.8, 14.3; HRMS (ESI): *m*/*z* calcd for C_20_H_37_O_3_ [M + H]^+^ 325.2737, found 325.2730.

### Passifetilactone B (2)

In a 5 mL Schlenk tube, pyran derivative 19a (0.055 g, 0.169 mmol) in anhydrous CHCl_3_ (1.5 mL) was purged with N_2_ gas and stirred at room temperature for homogeneity. Then, [Ir(COD)Cl]_2_ (0.0028 g, 0.004 mmol) and 2,6-dichlorobenzoic acid (0.016 g, 0.0847 mmol) were added sequentially and stirred for 18 h and monitored by TLC. After completion of reaction, the solvent was evaporated under reduced pressure and the crude residue was purified by silica gel column chromatography to afford Passifetilactone B (2) as an amorphous white solid (0.0503 g, 93% yield). TLC: *R*_f_ = 0.3 (SiO_2_, 40% EtOAc : hexane). [*α*]^25^_D_ = +14.6 (*c* = 2.0, MeOH). IR (CHCl_3_) 3400, 2913, 2848, 1685, 1624, 1470, 1397, 1280, 1219, 1167, 1075, 830, 772, 719, 646 cm^−1^; ^1^H NMR (400 MHz, CDCl_3_) *δ* 7.03–6.99 (m, 1H), 6.1 (d, *J* = 9.63 Hz, 1H), 4.33–4.28 (m, 1H), 4.07–4.04 (m, 1H), 2.69 (br, s, 1H), 1.94–1.87 (m, 1H), 1.82–1.76 (m, 1H), 1.55–1.50 (m, 1H), 1.34–1.25 (m, 25H), 0.87 (t, *J* = 7.0 Hz, 3H); ^13^C{^1^H} NMR (101 MHz, CDCl_3_) *δ* 164.3, 144.7, 144.6, 144.6, 123.1, 81.2, 62.2, 32.1, 30.1, 29.84, 29.82, 29.8, 29.73, 29.64, 29.6, 29.5, 25.1, 22.8, 14.3; HRMS (ESI): *m*/*z* calcd for C_20_H_37_O_3_ [M + H]^+^ 325.2737, found 325.2740.

### (2*S*,3*S*)-6-Oxo-2-pentadecyl-3,6-dihydro-2H-pyran-3-yl acetate (20)

In a 10 mL two-neck round-bottom flask, compound 2 (0.045 g, 0.138 mmol) in CH_2_Cl_2_ (2 mL) was added pyridine (0.022 mL, 0.277 mmol), DMAP (0.0016 g, 0.0138 mmol), and Ac_2_O (0.0196 mL, 0.208 mmol) sequentially and stirred at room temperature for 20 min. After completion of reaction, the reaction mixture was diluted with CH_2_Cl_2_ (5 mL) and quenched with 1 M HCl solution. The aqueous layer was extracted with CH_2_Cl_2_ (10 mL × 3), dried over Na_2_SO_4_, and filtered. The solvent was evaporated under reduced pressure, and the crude product was purified by silica gel column chromatography to afford the desired product 20 as an amorphous white solid (0.0477 g, 95%). TLC: *R*_f_ = 0.5 (SiO_2_, 30% EtOAc : hexane). [*α*]^25^_D_ = +316.7 (*c* = 0.8, CHCl_3_). IR (CHCl_3_) 2952, 2920, 2850, 1729, 1712, 1634, 1470, 1375, 1259, 1233, 1152, 1112, 1067, 1025, 966, 824, 771 cm^−1^; ^1^H NMR (400 MHz, CDCl_3_) *δ* 6.98–6.94 (m, 1H), 6.21 (d, *J* = 9.63 Hz, 1H), 5.17 (dd, *J* = 2.63, 5.16 Hz, 1H), 4.46–4.42 (m, 1H), 2.10 (s, 3H), 1.90–1.82 (m, 1H), 1.66–1.62 (m, 1H), 1.57–1.51 (m, 1H), 1.31–1.25 (m, 25H), 0.87 (t, *J* = 7.13 Hz, 3H); ^13^C{^1^H} NMR (101 MHz, CDCl_3_) *δ* 170.3, 163.1, 140.4, 125.2, 79.0, 63.2, 32.0, 30.2, 29.8, 29.8, 29.8, 29.8, 29.7, 29.6, 29.5, 29.5, 29.5, 29.4, 25.0, 22.8, 20.7, 14.2; HRMS (ESI): *m*/*z* calcd for C_22_H_39_O_4_ [M + H]^+^ 367.2843, found 367.2842.

### 6-Pentadecyl-2*H*-pyran-2-one (20a)

In a two-necked 25 mL round bottle flask, a solution of 20 and Et_3_N in THF was added HCO_2_NH_4_ and Pd(PPh_3_)_4_. The resulting mixture was refluxed for 3 h, and after completion of the reaction, the mixture was diluted with EtOAc, washed with brine, and the crude product was purified by silica gel column chromatography to afford 20a. IR (CHCl_3_) 2921, 2852, 1738, 1634, 1558, 1465, 1376, 1084, 979, 795, 721 cm^−1^; ^1^H NMR (400 MHz, CDCl_3_) *δ* 7.30–7.28 (m, 1H), 6.17 (d, *J* = 9.38 Hz, 1H), 5.99 (d, *J* = 6.63 Hz, 1H), 2.50 (t, *J* = 7.63 Hz, 2H), 1.70–1.66 (m, 2H), 1.28 (s, 24H), 0.91 (t, *J* = 7.13 Hz, 3H); ^13^C{^1^H} NMR (101 MHz, CDCl_3_) *δ* 167.0, 163.1, 143.9, 113.2, 102.7, 34.0, 32.1, 29.82, 29.79, 29.76, 29.72, 29.6, 29.5, 29.4, 29.1, 27.0, 22.8, 14.2; HRMS (ESI): *m*/*z* calcd for C_20_H_35_O_2_ [M + H]^+^ 307.2632 found 307.2629.

### (*S*)-6-Pentadecyl-3,6-dihydro-2*H*-pyran-2-one (21)

In a 10 mL two-neck round-bottom flask, solution of compound 20 (0.039 g, 0.106 mmol) in THF (3.5 mL) was added zinc powder (0.069 g, 1.06 mmol), and the reaction was stirred for 5 min at room temperature. Then, a saturated aqueous solution of NH_4_Cl (3.5 mL) was added, and the mixture was stirred overnight. After completion of the reaction, the reaction mixture was filtered over Celite and washed with EtOAc. The aqueous layer was then extracted with EtOAc (10 mL × 3), dried over Na_2_SO_4_, and filtered. The solvent was evaporated under reduced pressure, and the crude product was purified by silica gel column chromatography to afford the desired product 21 as an amorphous white solid (0.029 g, 88%). TLC: *R*_f_ = 0.4 (SiO_2_, 30% EtOAc : hexane). [*α*]^25^_D_ = +105.3 (*c* = 0.3, CHCl_3_). IR (CHCl_3_) 2954, 2914, 2848, 1730, 1469, 1376, 1366, 1225, 1172, 1083, 1046, 941, 772, 717, 689 cm^−1^; ^1^H NMR (400 MHz, CDCl_3_) *δ* 5.84–5.83 (m, 2H), 5.99–4.95 (m, 1H), 3.06–3.04 (m, 2H), 1.74–1.69 (m, 2H), 1.29–1.25 (m, 26H), 0.88 (t, *J* = 7.13 Hz, 3H); ^13^C{^1^H} NMR (101 MHz, CDCl_3_) *δ* 169.2, 126.7, 121.4, 79.8, 35.7, 31.9, 29.9, 29.83, 29.79, 29.6, 29.5, 29.4, 29.3, 24.3, 22.7, 14.1; HRMS (ESI): *m*/*z* calcd for C_20_H_37_O_2_ [M + H]^+^ 309.2788, found 309.2785.

### Passifetilactone C (3)

In a 10 mL two-neck round-bottom flask, a solution of the compound 13 (0.022 g, 0.071 mmol) in anhydrous THF (1 mL) was added DBU (0.021 mL, 0.142 mmol) and stirred for 2 h at room temperature. After completion of the reaction, the solvent was removed under reduced pressure, and the crude residue was purified on silica gel column chromatography to afford Passifetilactone C (3) as an amorphous white solid (0.0175 g, 83%). TLC: *R*_f_ = 0.4 (SiO_2_, 40% EtOAc : hexane). [*α*]^25^_D_ = +18.4 (*c* = 0.5, CHCl_3_). IR (CHCl_3_) 2953, 2916, 2849, 1723, 1690, 1468, 1391, 1268, 1161, 1127, 1031, 969, 951, 861, 819, 772, 720, 665 cm^−1^; ^1^H NMR (400 MHz, CDCl_3_) *δ* 6.89–6.85 (m, 1H), 6.02 (ddd, *J* = 1.38, 3.36, 9.76 Hz, 1H), 4.41 (tdd, *J* = 5.38, 7.13, 10.63 Hz, 1H), 2.34–2.30 (m, 2H), 1.79–1.75 (m, 1H), 1.65–1.62 (m, 1H), 1.51–1.48 (m, 1H), 1.41–1.38 (t, *J* = m, 1H), 1.25 (br, s, 24H), 0.87 (t, *J* = 7.0 Hz, 3H); ^13^C{^1^H} NMR (101 MHz, CDCl_3_) *δ* 164.8, 145.2, 121.6, 78.2, 35.0, 32.1, 29.83, 29.8, 29.77, 29.68, 29.61, 29.53, 29.5, 25.0, 22.8, 14.3; HRMS (ESI): *m*/*z* calcd for C_20_H_37_O_2_ [M + H]^+^ 309.2788, found 309.2780.

### 8-((*Tert*-Butyldimethylsilyl)oxy)octan-1-ol (S-1)

In a 250 mL two-neck round-bottom flask, a suspension of NaH (60% dispersion in mineral oil) (1.36 g, 34.2 mmol) in anhydrous THF (80 mL), cooled at 0 °C. To it was added a solution of 1,8-octanediol (22) (5 g, 34.2 mmol) in anhydrous THF (20 mL). The reaction mixture was stirred for 1 h, followed by portion-wise addition of TBSCl (5.157 g, 34.2 mmol). The mixture was then allowed to warm to room temperature and stirred for an additional 15 h. After completion of the reaction, it was quenched with a saturated aqueous solution of NH_4_Cl. The organic layer was separated, and the aqueous layer was extracted with EtOAc (60 mL × 3), dried over Na_2_SO_4_, and filtered. The solvent was evaporated under reduced pressure, and the crude product (S1) was directly forwarded for the next step without any further purification (8.2 g, 92% crude yield). TLC: *R*_f_ = 0.5 (SiO_2_, 20% EtOAc : hexane).

### 8-((*Tert*-Butyldimethylsilyl)oxy)octanal (23)

In a 100 mL two-neck round-bottom flask, aliphatic alcohol S-1 (3 g, 9.72 mmol) in anhydrous CH_2_Cl_2_ (30 mL) was cooled at 0 °C. To it was added Dess–Martin periodinane (6.15 g, 14.5 mmol) portion-wise, followed by the addition of solid NaHCO_3_ (0.98 g, 11.6 mmol), and the resulting suspension was stirred at the same temperature for 30 minutes. After completion of the reaction, it was quenched with a 1 : 1 ratio of a saturated aqueous solution of NaHCO_3_ and Na_2_S_2_O_3_, and the aqueous layer was extracted with CH_2_Cl_2_ (50 mL × 3). Then, the combined organic layers were washed with brine, dried over Na_2_SO_4_, filtered, and the solvent was evaporated under reduced pressure. The resulting crude product was purified by silica gel column chromatography to afford the desired product 23 as a yellow liquid (2.14 g, 85%). TLC: *R*_f_ = 0.6 (SiO_2_, 20% EtOAc : hexane). IR (CHCl_3_) 2922, 2844, 1710, 1462, 1411, 1253, 1096, 1005, 774, 661 cm^−1^; ^1^H NMR (400 MHz, CDCl_3_) *δ* 9.72–9.71 (m, 1H), 3.55 (dt, *J* = 2.63, 6.5 Hz, 2H), 2.39–2.35 (m, 2H), 1.59–1.57 (m, 2H), 1.46–1.44 (m, 2H), 1.28 (br, s, 6H), 0.85 (s, 10H), 0.00 (d, *J* = 3.0 Hz, 6H); ^13^C{^1^H} NMR (101 MHz, CDCl_3_) *δ* 202.7, 63.2, 43.9, 32.8, 29.2, 26.0, 25.7, 22.1, 18.4, −5.2; HRMS (ESI): *m*/*z* calcd for C_14_H_31_O_2_Si [M + H]^+^ 259.2088, found 259.2080.

### Heptyltriphenylphosphonium bromide (24)

In a single neck round bottle flask (50 mL), a stirred solution of 1-bromoheptane (0.5 g, 2.79 mmol) and PPh_3_ (0.878 g, 3.35 mmol) in toluene (8 mL), was refluxed for 36 h. After completion of reaction the solvent was evaporated and the crude product was dissolved into the DCM (3 mL) and then added dropwise to the diethyl ether for 1 h, the precipitate was filtered and dried over vacuum, afford the wittig salt 24 (1.18 g, 96%), which used into next step without further purification.

### (*Z*)-*Tert*-Butyldimethyl(pentadec-8-en-1-yloxy)silane (25)

In a 100 mL two-neck round-bottom flask, a stirred solution of the Wittig salt 24 (2.042 g, 4.64 mmol) in anhydrous THF (10 mL) was cooled at −78 °C, and *n*-BuLi (2.9 mL, 4.64 mmol, 1.6 M in hexane) was added dropwise and stirred for 30 min at the same temperature. Next, a solution of aldehyde 23 (1 g, 3.86 mmol) in anhydrous THF (5 mL) was added and the reaction mixture was allowed to warm to 0 °C for 4 h. After completion, the reaction mixture was quenched by saturated aqueous solution of NH_4_Cl, and the aqueous layer was extracted with EtOAc (30 mL × 3), dried over Na_2_SO_4_, and filtered. The solvent was evaporated under reduced pressure, and the crude product was purified by silica gel column chromatography to afford the desired product 25 as a yellow liquid (1.38 g, 84%). TLC: *R*_f_ = 0.9 (SiO_2_, 2% EtOAc : hexane); IR (CHCl_3_) 2953, 2924, 2854, 1462, 1386, 1253, 1099, 1005, 965, 834, 774, 723 cm^−1^. ^1^H NMR (400 MHz, CDCl_3_) *δ* 5.38–5.33 (m, 2H), 3.59 (t, *J* = 6.63 Hz, 2H), 2.02–1.96 (m, 4H), 1.51 (t, *J* = 6.38 Hz, 2H), 1.30 (br, s, 16H), 0.89–0.87 (m, 12H), 0.05 (s, 6H); ^13^C{^1^H} NMR (101 MHz, CDCl_3_) *δ* 130.6, 130.5, 130.1, 130.0, 63.5, 33.0, 32.8, 32.7, 31.94, 31.92, 29.9, 29.87, 29.77, 29.75, 29.5, 29.4, 29.3, 29.1, 29.0, 27.4, 27.3, 26.1, 25.9, 22.8, 18.5, 14.3, −5.1; HRMS (ESI): *m*/*z* calcd for C_21_H_44_OSi [M + H]^+^ 341.3234, found 341.3226.

### (*Z*)-Pentadec-8-en-1-ol (S-2)

In a 100 mL two-neck round-bottom flask, compound 25 (1.2 g, 3.52 mmol) in anhydrous THF (15 mL) was cooled at 0 °C. To it, was added TBAF (10.56 mL, 10.5 mmol; 1 M in THF) dropwise and the reaction mixture was stirred for 4 h. After completion of reaction, the reaction mixture was quenched by saturated aqueous solution of NH_4_Cl, and the aqueous layer was extracted with EtOAc (10 mL × 3), dried over Na_2_SO_4_, and filtered. The solvent was evaporated under reduced pressure, and the crude product was purified by silica gel column chromatography to afford the desired product S-2 as a yellow liquid (0.79 g, 99%). TLC: *R*_f_ = 0.3 (SiO_2_, 50% EtOAc : hexane); IR (CHCl_3_) 3330, 3004, 2922, 2853, 1463, 1404, 1377, 1350, 1219, 1056, 965, 772, 723 cm^−1^. ^1^H NMR (400 MHz, CDCl_3_) *δ* 5.39–5.30 (m, 2H), 3.61 (t, *J* = 6.6 Hz, 2H), 2.05–1.91 (m, 4H), 1.59–1.50 (m, 2H), 1.37–1.25 (m, 16H), 0.91–0.85 (m, 4H); ^13^C{^1^H} NMR (101 MHz, CDCl_3_) *δ* 130.6, 130.3, 130.1, 129.9, 63.1, 32.9, 32.72, 32.68, 31.9, 31.87, 29.9, 29.8, 29.73, 29.68, 29.45, 29.41, 29.36, 29.2, 29.1, 29.0, 27.3, 27.29, 25.84, 25.76, 22.8, 14.2, −3.5; HRMS (ESI): *m*/*z* calcd for C_16_H_33_O [M + H]^+^ 241.2526, found 241.2526.

### (*Z*)-15-Bromopentadec-7-ene (26)

In a 100 mL two-neck round-bottom flask, the corresponding alcohol S-2 (1 g, 4.41 mmol) was added CH_2_Cl_2_ (50 mL), and the flask was cooled at 0 °C. Then, carbon tetrabromide (2.99 g, 8.83 mmol) and triphenylphosphine (2.317 g, 8.83 mmol) were added, and the reaction mixture was allowed to stir at room temperature for the next 2 h. After completion, the solvent was removed under reduced pressure, and the crude residue was purified by silica gel column chromatography to afford the desired product 26 as a colourless liquid (1.151 g, 90%). IR (CHCl_3_) 2924, 2854, 2360, 2349, 2338, 1708, 1463, 1376, 1252, 1219, 968, 772, 724, 646 cm^−1^. TLC: *R*_f_ = 0.9 (SiO_2_, 100% hexane). ^1^H NMR (400 MHz, CDCl_3_) *δ* 5.39–5.33 (m, 2H), 3.40 (t, *J* = 6.88 Hz, 2H), 2.02–1.87 (m, 4H), 1.85 (quin, *J* = 7.5 Hz, 2H), 1.44–1.42 (m, 2H), 1.31–1.27 (m, 14H), 0.88 (t, *J* = 7.13 Hz, 3H); ^13^C{^1^H} NMR (101 MHz, CDCl_3_) *δ* 130.7, 130.3, 130.2, 129.8, 34.1, 33.0, 32.8, 32.7, 31.94, 31.91, 31.7, 29.9, 29.8, 29.6, 29.2, 29.1, 29.05, 28.99, 28.81, 28.77, 28.3, 27.4, 27.3, 22.8, 14.3; HRMS (ESI): *m*/*z* calcd for C_15_H_30_Br [M + H]^+^ 289.1525, found 289.2526.

### 9-((*Tert*-Butyldimethylsilyl)oxy)nonan-1-ol (S-3)

In a 250 mL two-neck round-bottom flask, a suspension of NaH (60% dispersion in mineral oil) (1.24 g, 31.2 mmol) in anhydrous THF (80 mL), cooled at 0 °C. To it was added a solution of 1,9-nonanediol (28) (5 g, 31.2 mmol) in anhydrous THF (20 mL). The reaction mixture was stirred for 1 h, followed by portion-wise addition of TBSCl (4.7 g, 31.2 mmol). The mixture was then allowed to warm to room temperature and stirred for an additional 15 h. After completion of the reaction, it was quenched with a saturated aqueous solution of NH_4_Cl. The organic layer was separated, and the aqueous layer was extracted with EtOAc (100 mL × 3), dried over Na_2_SO_4_, and filtered. The solvent was evaporated under reduced pressure, and the crude product was purified by silica gel column chromatography to afford the desired product S-3 as a yellow liquid (8.09 g, 94%). TLC: *R*_f_ = 0.5 (SiO_2_, 20% EtOAc : hexane). IR (CHCl_3_) 2925, 2854, 1710, 1462, 1411, 1360, 1253, 1096, 1005, 938, 835, 774, 661 cm^−1^; ^1^H NMR (400 MHz, CDCl_3_) *δ* 3.57–3.52 (m, 4H), 2.62–2.41 (m, 1H), 1.47 (dd, *J* = 7.13, 14.0 Hz, 4H), 1.25 (br, s, 10H), 0.86–0.84 (m, 9H), 0.00 (s, 6H); ^13^C{^1^H} NMR (101 MHz, CDCl_3_) *δ* 63.4, 62.8, 32.9, 32.8, 29.7, 29.5, 29.4, 26.0, 25.8, 25.7, 18.4, −5.2; HRMS (ESI): *m*/*z* calcd for C_15_H_35_O_2_Si [M − H]^−^ 275.2401, found 275.2401.

### 9-((*Tert*-Butyldimethylsilyl)oxy)nonanal (29)

In a 100 mL two-neck round-bottom flask, aliphatic alcohol S-3 (3 g, 10.9 mmol) in anhydrous CH_2_Cl_2_ (50 mL) was cooled at 0 °C. To it was added Dess–Martin periodinane (6.91 g, 16.3 mmol) portion-wise, followed by the addition of solid NaHCO_3_ (1.1 g, 13 mmol), and the resulting suspension was stirred at the same temperature for 30 minutes. After completion of the reaction, it was quenched with a 1 : 1 ratio of a saturated aqueous solution of NaHCO_3_ and Na_2_S_2_O_3_, and the aqueous layer was extracted with CH_2_Cl_2_ (100 mL × 3). Then, the combined organic layers were washed with brine, dried over Na_2_SO_4_, filtered, and the solvent was evaporated under reduced pressure. The resulting crude product was purified by silica gel column chromatography to afford the desired product 29 as a yellow liquid (2.49, 86%). TLC: *R*_f_ = 0.6 (SiO_2_, 20% EtOAc : hexane). IR (CHCl_3_) 2925, 2854, 1710, 1462, 1411, 1360, 1253, 1096, 1005, 938, 835, 774, 661 cm^−1^; ^1^H NMR (400 MHz, CDCl_3_) *δ* 9.75–9.74 (m, 1H), 3.59–3.56 (m, 2H), 2.42–2.38 (m, 2H), 1.62–1.57 (m, 2H), 1.48 (t, *J* = 6.13 Hz, 2H), 1.29 (br, s, 8H), 0.87 (s, 9H), 0.03 (s, 6H); ^13^C{^1^H} NMR (101 MHz, CDCl_3_) *δ* 203.1, 63.4, 44.0, 32.9, 29.5, 29.3, 29.2, 26.1, 25.9, 22.2, 18.5, −5.1; HRMS (ESI): *m*/*z* calcd for C_15_H_33_O_2_Si [M + H]^+^ 273.2244, found 273.2237.

### (*Z*)-*Tert*-Butyl(hexadec-9-en-1-yloxy)dimethylsilane (30)

In a 100 mL two-neck round-bottom flask, a stirred solution of the Wittig salt 24 (2.9 g, 6.6 mmol) in anhydrous THF (20 mL) was cooled at −78 °C, and *n*-BuLi (4.12 mL, 6.61 mmol, 1.6 M in hexane) was added dropwise and stirred for 30 min at the same temperature. Next, a solution of aldehyde 29 (1.5 g, 5.5 mmol) in anhydrous THF (10 mL) was added and the reaction mixture was allowed to warm to 0 °C for 4 h. After completion, the reaction mixture was quenched by saturated aqueous solution of NH_4_Cl, and the aqueous layer was extracted with EtOAc (50 mL × 3), dried over Na_2_SO_4_, and filtered. The solvent was evaporated under reduced pressure, and the crude product was purified by silica gel column chromatography to afford the desired product 30 as a yellow liquid (1.622 g, 83%). TLC: *R*_f_ = 0.9 (SiO_2_, 5% EtOAc : hexane). (*E*/*Z* ratio = 1 : 2.4). IR (CHCl_3_) 2924, 2854, 1462, 1254, 1098, 1005, 965, 833, 773, 722, 661 cm^−1^. ^1^H NMR (400 MHz, CDCl_3_) *δ* 5.41–5.31 (m, 2H), 3.60 (t, *J* = 6.6 Hz, 2H), 2.07–1.94 (m, 4H), 1.56–1.46 (m, 2H), 1.36–1.25 (m, 18H), 0.93–0.85 (m, 12H), 0.05 (s, 6H); ^13^C{^1^H} NMR (101 MHz, CDCl_3_) *δ* 130.5, 130.5, 130.1, 130.0, 63.5, 33.1, 32.8, 32.0, 31.9, 29.9, 29.8, 29.7, 29.68, 29.59, 29.4, 29.3, 29.2, 29.0, 27.4, 26.1, 26.0, 22.8, 18.5, 14.3, −5.1.

### (*Z*)-Hexadec-9-en-1-ol (S-4)

In a 100 mL two-neck round-bottom flask, compound 30 (1.45 g, 4.08 mmol) in anhydrous THF (20 mL) was cooled at 0 °C. To it, was added TBAF (12.2 mL, 12.2 mmol; 1 M in THF) dropwise and the reaction mixture was stirred for 4 h. After completion of reaction, the reaction mixture was quenched by saturated aqueous solution of NH_4_Cl, and the aqueous layer was extracted with EtOAc (50 mL × 3), dried over Na_2_SO_4_, and filtered. The solvent was evaporated under reduced pressure, and the crude product was purified by silica gel column chromatography to afford the desired product S-4 as a yellow liquid (0.975 g, 99%). TLC: *R*_f_ = 0.3 (SiO_2_, 30% EtOAc : hexane); IR (CHCl_3_) 3328, 3004, 2922, 2853, 1463, 1377, 1219, 1056, 966, 772, 722 cm^−1^; ^1^H NMR (400 MHz, CDCl_3_) *δ* 5.37–5.32 (m, 2H), 3.61 (t, *J* = 6.63 Hz, 2H), 2.01–1.95 (m, 4H), 1.75 (br, s, 1H), 1.56–1.51 (m, 2H), 1.29 (br, s, 18H), 0.87 (t, *J* = 6.63 Hz, 3H); ^13^C{^1^H} NMR (101 MHz, CDCl_3_) *δ* 130.5, 130.4, 130.0, 129.9, 63.0, 32.9, 32.72, 32.69, 31.89, 31.87, 29.8, 29.7, 29.62, 29.58, 29.53, 29.3, 29.2, 29.1, 28.9, 27.32, 27.29, 25.9, 22.8, 14.2; HRMS (ESI): *m*/*z* calcd for C_16_H_33_O [M + H]^+^ 241.2526, found 241.2523.

### (*Z*)-Hexadec-9-enal (31)

In a 100 mL two-neck round-bottom flask, aliphatic alcohol S-4 (0.95 g, 3.95 mmol) in anhydrous CH_2_Cl_2_ (20 mL) was cooled at 0 °C. To it was added Dess–Martin periodinane (2.51 g, 5.92 mmol) portion-wise, followed by the addition of solid NaHCO_3_ (0.398 g, 4.74 mmol), and the resulting suspension was stirred at the same temperature for 30 minutes. After completion of the reaction, it was quenched with a 1 : 1 ratio of a saturated aqueous solution of NaHCO_3_ and Na_2_S_2_O_3_, and the aqueous layer was extracted with CH_2_Cl_2_ (50 mL × 3). Then, the combined organic layers were washed with brine, dried over Na_2_SO_4_, filtered, and the solvent was evaporated under reduced pressure. The resulting crude product was purified by silica gel column chromatography to afford the desired product 31 as a yellow liquid (0.753 g, 80%). TLC: *R*_f_ = 0.6 (SiO_2_, 30% EtOAc : hexane). IR (CHCl_3_) 2922, 2853, 1728, 1711, 1463, 1377, 966, 772, 723 cm^−1^; ^1^H NMR (400 MHz, CDCl_3_) *δ* 9.76 (t, *J* = 1.75 Hz, 1H), 5.39–5.30 (m, 2H), 2.42 (dt, *J* = 1.75, 7.4 Hz, 2H), 2.04–1.93 (m, 4H), 1.64–1.61 (m, 2H), 1.30 (br, s, 16H), 0.88 (t, *J* = 7.13, 3H); ^13^C{^1^H} NMR (101 MHz, CDCl_3_) *δ* 203.1, 130.7, 130.3, 130.2, 129.8, 44.1, 32.75, 32.68, 31.93, 31.9, 29.9, 29.8, 29.75, 29.68, 29.4, 29.35, 29.28, 29.2, 29.1, 29.03, 29.0, 27.4, 27.3, 22.8, 22.2, 14.2; HRMS (ESI): *m*/*z* calcd for C_16_H_31_O [M + H]^+^ 239.2369, found 239.2366.

### (*Z*)-1-(Furan-2-yl)hexadec-9-en-1-ol (27)

In a 50 mL two-neck round-bottom flask, furan 32 (0.36 g, 5.28 mmol) in anhydrous THF (10 mL) was taken and cooled at −78 °C, and to it was added *n*-BuLi (3.3 mL, 2.96 mmol; 1.6 M in hexanes). The reaction mixture was allowed to warm up to 0 °C in the next 1 h, and a solution of aldehyde 31 (0.702 g, 2.96) in anhydrous THF (6 mL) at −78 °C and left stirring up to 0 °C for 4 h. After completion of reaction, the reaction mixture was quenched by saturated aqueous solution of NH_4_Cl, and the aqueous layer was extracted with EtOAc (20 mL × 3), dried over Na_2_SO_4_, and filtered. The solvent was evaporated under reduced pressure, and the crude product was purified by silica gel column chromatography to afford the desired product 27 as a yellow liquid (1.411 g, 88%). TLC: *R*_f_ = 0.4 (SiO_2_, 20% EtOAc : hexane). IR (CHCl_3_) 3403, 3003, 2922, 2853, 1628, 1464, 1377, 1309, 1221, 1150, 1008, 884, 840, 731 cm^−1^; ^1^H NMR (400 MHz, CDCl_3_) *δ* 7.36 (d, *J* = 1.13 Hz, 1H), 6.32 (dd, *J* = 1.75, 3.25 Hz, 1H), 6.22 (d, *J* = 3.13 Hz, 1H), 5.39–5.33 (m, 2H), 4.66 (t, *J* = 6.75 Hz, 1H), 2.02–1.97 (m, 5H), 1.86–1.81 (m, 2H), 1.46–1.41 (m, 1H), 1.29 (br, s, 16H), 0.88 (t, *J* = 7.13 Hz, 3H); ^13^C{^1^H} NMR (101 MHz, CDCl_3_) *δ* 157.1, 142.0, 130.6, 130.4, 130.1, 129.9, 110.2, 105.9, 68.0, 35.7, 32.73, 32.7, 31.91, 31.88, 29.9, 29.7, 29.54, 29.49, 29.3, 29.2, 29.1, 29.0, 27.3, 27.3, 25.7, 22.8, 14.2; HRMS (ESI): *m*/*z* calcd for C_20_H_33_O_2_ [M − H]^−^ 305.2475, found 305.2473.

### (*Z*)-1-(Furan-2-yl)hexadec-9-en-1-one (17b)

In a 100 mL two-neck round-bottom flask, furyl alcohol 27 (1.1 g, 3.58 mmol) in anhydrous CH_2_Cl_2_ (30 mL) was cooled at 0 °C. To it was added Dess–Martin periodinane (2.28 g, 5.38 mmol) portion-wise, followed by the addition of solid NaHCO_3_ (0.361 g, 4.306 mmol), and the resulting suspension was stirred at the same temperature for 40 minutes. After completion of the reaction, it was quenched with a 1 : 1 ratio of a saturated aqueous solution of NaHCO_3_ and Na_2_S_2_O_3_, and the aqueous layer was extracted with CH_2_Cl_2_ (50 mL × 3). Then, the combined organic layers were washed with brine, dried over Na_2_SO_4_, filtered, and the solvent was evaporated under reduced pressure. The resulting crude product was purified by silica gel column chromatography to afford the desired product 17b as a yellow liquid (0.739 g, 67%). TLC: *R*_f_ = 0.6 (SiO_2_, 20% EtOAc : hexane). IR (CHCl_3_) 3003, 2923, 2853, 1678, 1568, 1467, 1393, 1256, 1157, 1082, 1010, 968, 883, 830, 755, 724, 646 cm^−1^; ^1^H NMR (400 MHz, CDCl_3_) *δ* 7.56 (d, *J* = 1.0 Hz, 1H), 7.16 (d, *J* = 3.5 Hz, 1H), 6.51 (dd, *J* = 1.63, 3.5 Hz, 1H), 5.37–5.31 (m, 2H), 2.79 (t, *J* = 7.5 Hz, 2H), 2.04–1.93 (m, 4H), 1.72–1.66 (m, 2H), 1.31–1.26 (m, 16H), 0.87 (t, *J* = 7.13 Hz, 3H); ^13^C{^1^H} NMR (101 MHz, CDCl_3_) *δ* 189.9, 153.0, 146.2, 130.6, 130.3, 130.1, 129.9, 116.9, 112.2, 38.6, 32.71, 32.66, 31.89, 31.86, 29.83, 29.8, 29.71, 29.68, 29.4, 29.36, 29.2, 29.1, 29.06, 28.9, 27.33, 27.27, 24.4, 22.8, 14.2; HRMS (ESI): *m*/*z* calcd for C_20_H_33_O_2_ [M + H]^+^ 305.2475, found 305.2475.

### (*S*,*Z*)-1-(Furan-2-yl)hexadec-9-en-1-ol (18b)

In a 50 mL two-neck round-bottom flask, furyl ketone 17b (0.3 g, 0.985 mmol) in anhydrous THF (10 mL) was cooled to −78 °C under an argon atmosphere. To it was added a premixed solution of (*R*)-2-methyl-CBS-oxazaborolidine (0.344 mL, 0.344 mmol; 1 M in THF) and borane dimethylsulfide (0.665 mL, 1.33 mmol; 2 M in THF) and allowed to stir at the same temperature for 6 h. After completion, the reaction mixture was quenched by saturated aqueous solution of NH_4_Cl, and the aqueous layer was extracted with EtOAc (20 mL × 3), dried over Na_2_SO_4_, and filtered. The solvent was evaporated under reduced pressure, and the crude product was purified by column chromatography using neutral alumina oxide to afford the desired product 18b as a colorless oil (0.288 g, 95% yield). TLC: *R*_f_ = 0.4 (SiO_2_, 20% EtOAc : hexane). [*α*]^25^_D_ = −5.3 (*c* = 2.1, MeOH). The enantiomeric purity was determined by HPLC (CHIRALCEL OD-H column, *n*-hexane : *i*-PrOH = 97 : 3, flow rate = 1 mL min^−1^, *λ* = 235 nm, *t*_major_ = 7.96 min, *t*_minor_ = 7.24 min), er = 94 : 6. IR (CHCl_3_) 3403, 3003, 2922, 2853, 1628, 1464, 1377, 1309, 1221, 1150, 1008, 884, 840, 731 cm^−1^; ^1^H NMR (400 MHz, CDCl_3_) *δ* 7.37 (d, *J* = 1.0 Hz, 1H), 6.32 (dd, *J* = 1.88, 3.13 Hz, 1H), 6.22 (d, *J* = 3.25 Hz, 1H), 5.39–5.33 (m, 2H), 4.66 (t, *J* = 6.75 Hz, 1H), 2.02–1.95 (m, 5H), 1.87–1.82 (m, 2H), 1.44–1.39 (m, 1H), 1.29 (br, s, 16H), 0.88 (t, *J* = 7.13 Hz, 3H); ^13^C{^1^H} NMR (101 MHz, CDCl_3_) *δ* 157.1, 142.0, 130.6, 130.4, 130.1, 129.9, 110.2, 105.9, 67.9, 35.7, 32.74, 32.7, 31.91, 31.89, 29.9, 29.7, 29.55, 29.5, 29.3, 29.2, 29.1, 29.0, 27.35, 27.31, 25.7, 22.8, 14.2; HRMS (ESI): *m*/*z* calcd for C_20_H_33_O_2_ [M − H]^−^ 305.2475, found 305.2473.

### (2*S*)-6-Hydroxy-2-((*Z*)-pentadec-8-en-1-yl)-2*H*-pyran-3(6*H*)-one (19b)

In a 10 mL two-neck round-bottom flask, furyl alcohol 18b (0.1 g, 0.326 mmol) in a 4 : 1 ratio of THF/H_2_O (2 mL) was cooled to 0 °C. To the above solution, solid NaHCO_3_ (0.0548 g, 0.652 mmol), NaOAc·3H_2_O (0.044 g, 0.326 mmol), and *N*-bromosuccinimide (0.058 g, 0.326 mmol) were added sequentially, and the reaction mixture was allowed to stir for 10 min. After completion of reaction, the reaction mixture was quenched by saturated aqueous solution of NaHCO_3_, and the aqueous layer was extracted with EtOAc (5 mL × 3), dried over Na_2_SO_4_, and filtered. The solvent was evaporated under reduced pressure, and the crude product was purified by silica gel column chromatography to afford the desired product 19b as a pale-yellow liquid (0.023 g, 18%). TLC: *R*_f_ = 0.3 (SiO_2_, 30% EtOAc : hexane). IR (CHCl_3_) 3411, 3003, 2922, 2853, 1691, 1631, 1464, 1375, 1261, 1153, 1085, 1030, 965, 804, 758, 723, 691 cm^−1^; ^1^H NMR (400 MHz, CDCl_3_) *δ* 6.93–6.87 (m, 1H), 6.15–6.08 (m, 1H), 5.64 (br, s, 1H), 5.38–5.33 (m, 2H), 4.55 (dd, *J* = 3.88, 8.13 Hz, 1H), 4.22–4.05 (m, 1H), 3.13 (br, s, 1H), 2.04–1.95 (m, 4H), 1.73–1.64 (m, 2H), 1.42–1.39 (m, 2H), 1.29 (br, s, 16H), 0.88 (t, *J* = 7 Hz, 3H); ^13^C{^1^H} NMR (101 MHz, CDCl_3_) *δ* 196.8, 196.4, 147.7, 144.4, 130.6, 130.4, 130.1, 130.0, 128.9, 127.8, 91.0, 87.8, 79.1, 74.4, 74.3, 60.0, 59.9, 32.75, 32.72, 31.92, 31.89, 30.8, 29.9, 29.77, 29.75, 29.5, 29.49, 29.4, 29.2, 29.1, 29.0, 28.8, 28.6, 27.9, 27.4, 27.3, 25.3, 25.1, 22.8, 22.7, 14.2, 14.2; HRMS (ESI): *m*/*z* calcd for C_20_H_37_O_3_ [M + H]^+^ 323.2581, found 323.2571.

### 9-((*Tert*-Butyldimethylsilyl)oxy)-1-(furan-2-yl)nonan-1-ol (36)

In a 100 mL two-neck round-bottom flask, furan 32 (1.5 g, 22.3 mmol) in anhydrous THF (30 mL) was taken and cooled at −78 °C, and to it was added *n*-BuLi (13.93 mL, 22.3 mmol; 1.6 M in hexanes). The reaction mixture was allowed to warm up to 0 °C in the next 1 h, and a solution of aldehyde 24 (3.351 g, 12.3 mmol) in anhydrous THF (10 mL) at −78 °C and left stirring up to 0 °C for 4 h. After completion of reaction, the reaction mixture was quenched by a saturated aqueous solution of NH_4_Cl, and the aqueous layer was extracted with EtOAc (50 mL × 3), dried over Na_2_SO_4_, and filtered. The solvent was evaporated under reduced pressure, and the crude product was purified by silica gel column chromatography to afford the desired product 36 as a yellow liquid (6.318 g, 87%). TLC: *R*_f_ = 0.4 (SiO_2_, 20% EtOAc : hexane). IR (CHCl_3_) 3383, 2927, 2855, 1504, 1462, 1387, 1254, 1150, 1095, 1005, 938, 884, 833, 773, 731, 661 cm^−1^; ^1^H NMR (400 MHz, CDCl_3_) *δ* 7.35 (m, 1H), 6.31–6.3 (m, 1H), 6.20 (d, *J* = 3.0 Hz, 1H), 4.64 (t, *J* = 6.25 Hz, 1H), 3.58 (t, *J* = 6.63 Hz, 2H), 2.22–2.16 (m, 1H), 1.87–1.81 (m, 2H), 1.51–1.46 (m, 2H), 1.28 (br, s, 10H), 0.89 (s, 9H), 0.04 (s, 6H); ^13^C{^1^H} NMR (101 MHz, CDCl_3_) *δ* 157.1, 141.9, 110.2, 105.8, 67.9, 63.4, 35.7, 33.0, 29.6, 29.4, 26.1, 25.9, 25.6, 18.5, −5.2; HRMS (ESI): *m*/*z* calcd for C_19_H_35_O_3_Si [M − H]^−^ 339.2350, found 339.2341.

### 9-((*Tert*-Butyldimethylsilyl)oxy)-1-(furan-2-yl)nonan-1-one (37)

In a 100 mL two-neck round-bottom flask, furyl alcohol 36 (2 g, 5.87 mmol) in anhydrous CH_2_Cl_2_ (40 mL) was cooled at 0 °C. To it was added Dess–Martin periodinane (3.736 g, 8.81 mmol) portion-wise, followed by the addition of solid NaHCO_3_ (0.592 g, 7.04 mmol), and the resulting suspension was stirred at the same temperature for 40 minutes. After completion of the reaction, it was quenched with a 1 : 1 ratio of a saturated aqueous solution of NaHCO_3_ and Na_2_S_2_O_3_, and the aqueous layer was extracted with CH_2_Cl_2_ (60 mL × 3). Then, the combined organic layers were washed with brine, dried over Na_2_SO_4_, filtered, and the solvent was evaporated under reduced pressure. The resulting crude product was purified by silica gel column chromatography to afford the desired product 37 as a pale-yellow liquid (1.488 g, 75%). TLC: *R*_f_ = 0.6 (SiO_2_, 20% EtOAc : hexane). IR (CHCl_3_) 2927, 2855, 1679, 1569, 1469, 1391, 1360, 1253, 1156, 1095, 1008, 883, 834, 773, 661 cm^−1^; ^1^H NMR (400 MHz, CDCl_3_) *δ* 7.57 (dd, *J* = 0.63, 1.63 Hz, 1H), 7.17 (dd, *J* = 0.63, 3.63 Hz, 1H), 6.52 (dd, *J* = 1.75, 3.63 Hz, 1H), 3.59 (t, *J* = 6.63 Hz, 2H), 2.80 (t, *J* = 7.63 Hz, 2H), 1.73–1.67 (m, 2H), 1.51–1.46 (m, 2H), 1.37–1.30 (m, 8H), 0.89 (s, 9H), 0.04 (s, 6H); ^13^C{^1^H} NMR (101 MHz, CDCl_3_) *δ* 190.0, 153.0, 146.3, 116.9, 112.2, 63.4, 38.7, 33.0, 29.9, 29.5, 29.43, 29.40, 26.1, 25.9, 24.5, 18.5, −5.1; HRMS (ESI): *m*/*z* calcd for C_19_H_35_O_3_Si [M + H]^+^ 339.2350, found 339.2350.

### (*S*)-9-((*Tert*-Butyldimethylsilyl)oxy)-1-(furan-2-yl)nonan-1-ol (38)

In a 100 mL two-neck round-bottom flask, furyl ketone 37 (1 g, 2.953 mmol) in anhydrous THF (15 mL) was cooled to −78 °C under an argon atmosphere. To it was added a premixed solution of (*R*)-2-methyl-CBS-oxazaborolidine (1.03 mL, 1.03 mmol; 1 M in THF) and borane dimethylsulfide (2 mL, 4.017 mmol; 2 M in THF) and allowed to stir at the same temperature for 6 h. After completion, the reaction mixture was quenched by saturated aqueous solution of NH_4_Cl, and the aqueous layer was extracted with EtOAc (30 mL × 3), dried over Na_2_SO_4_, and filtered. The solvent was evaporated under reduced pressure, and the crude product was purified by column chromatography using neutral alumina oxide to afford the desired product 38 as a colorless oil (0.967 g, 96% yield). TLC: *R*_f_ = 0.6 (SiO_2_, 20% EtOAc : hexane). [*α*]^25^_D_ = −10.73 (*c* = 1, MeOH). The enantiomeric purity was determined by HPLC (CHIRALCEL OD-H column, *n*-hexane : *i*-PrOH = 97 : 3, flow rate = 1 mL min^−1^, *λ* = 235 nm, *t*_major_ = 8.07 min, *t*_minor_ = 7.35 min), er = 99 : 1. IR (CHCl_3_) 3383, 2927, 2855, 1504, 1462, 1387, 1254, 1150, 1095, 1005, 938, 884, 833, 773, 731, 661 cm^−1^; ^1^H NMR (400 MHz, CDCl_3_) *δ* 7.31 (d, *J* = 0.9 Hz, 1H), 6.30–6.25 (m, 1H), 6.17 (d, *J* = 3.3 Hz, 1H), 4.60 (t, *J* = 6.8 Hz, 1H), 3.54 (t, *J* = 6.6 Hz, 2H), 2.10 (br, s, 1H), 1.83–1.74 (m, 2H), 1.47–1.42 (m, 2H), 1.24 (br, s, 10H), 0.85 (s, 9H), 0.00 (s, 6H); ^13^C{^1^H} NMR (101 MHz, CDCl_3_) *δ* 157.1, 141.9, 110.2, 105.8, 67.9, 63.4, 35.7, 33.0, 29.6, 29.4, 26.1, 25.9, 25.6, 18.5, −5.1; HRMS (ESI): *m*/*z* calcd for C_19_H_35_O_3_Si [M − H]^−^339.2350, found 339.2341.

### (2*S*)-2-(8-((*Tert*-Butyldimethylsilyl)oxy)octyl)-6-hydroxy-2*H*-pyran-3(6*H*)-one (39)

In a 100 mL two-neck round-bottom flask, furyl alcohol 38 (0.781 g, 2.29 mmol) in a 4 : 1 ratio of THF/H_2_O (10 mL) was cooled to 0 °C. To the above solution, solid NaHCO_3_ (0.385 g, 4.59 mmol), NaOAc·3H_2_O (0.311 g, 2.29 mmol), and *N*-bromosuccinimide (0.407 g, 2.29 mmol) were added sequentially, and the reaction mixture was allowed to stir for 10 min. After completion of reaction, the reaction mixture was quenched by saturated aqueous solution of NaHCO_3_, and the aqueous layer was extracted with EtOAc (20 mL × 3), dried over Na_2_SO_4_, and filtered. The solvent was evaporated under reduced pressure, and the crude product was purified by silica gel column chromatography to afford the desired product 39 as a pale-yellow liquid (0.659 g, 88%). TLC: *R*_f_ = 0.3 (SiO_2_, 30% EtOAc : hexane). [*α*]^25^_D_ = +57.56 (*c* = 0.6, CHCl_3_). IR (CHCl_3_) 3392, 2927, 2855, 1694, 1463, 1387, 1254, 1151, 1091, 1035, 938, 835, 775, 662 cm^−1^; ^1^H NMR (400 MHz, CDCl_3_) *δ* 6.94–6.87 (m, 1H), 6.15–6.08 (m, 1H), 5.65–5.63 (m, 1H), 4.55 (dd, *J* = 3.75, 8.13 Hz, 1H), 3.59 (t, *J* = 6.63 Hz, 2H), 1.96–1.88 (m, 1H), 1.64 (br, s, 2H), 1.50 (t, *J* = 6.63 Hz, 2H), 1.44–1.41 (m, 2H), 1.29 (br, s, 8H), 0.89 (s, 9H), 0.04 (s, 6H); ^13^C{^1^H} NMR (101 MHz, CDCl_3_) *δ* 196.8, 196.4, 147.8, 144.4, 129.0, 127.8, 91.0, 87.8, 79.1, 74.3, 63.6, 33.0, 30.7, 29.7, 29.5, 29.5, 29.4, 29.4, 26.1, 25.9, 25.2, 25.1, 18.5, −5.1; HRMS (ESI): *m*/*z* calcd for C_19_H_37_O_4_Si [M + H]^+^ 357.2456, found 357.2442.

### (5*S*,6*S*)-6-(8-((*Tert*-Butyldimethylsilyl)oxy)octyl)-5-hydroxy-5,6-dihydro-2*H*-pyran-2-one (40)

In a 10 mL Schlenk tube, pyran derivative 39 (0.2 g, 0.56 mmol) in anhydrous CHCl_3_ (4 mL) was purged with N_2_ gas and stirred at room temperature for homogeneity. Then, [Ir(COD)Cl]_2_ (0.0094 g, 0.014 mmol) and 2,6-dichlorobenzoic acid (0.0535 g, 0.28 mmol) were added sequentially and stirred for 18 h and monitored by TLC. After completion of reaction, the solvent was evaporated under reduced pressure, and the crude residue was purified by silica gel column chromatography to afford desired product 40 as a yellow liquid (0.182 g, 91% yield). TLC: *R*_f_ = 0.4 (SiO_2_, 40% EtOAc : hexane). [*α*]^25^_D_ = +59.6 (*c* = 0.9, CHCl_3_). IR (CHCl_3_) 3396, 2927, 2855, 1705, 1629, 1462, 1386, 1253, 1095, 1051, 1005, 897, 831, 774, 722, 661 cm^−1^; ^1^H NMR (400 MHz, CDCl_3_) *δ* 7.00 (dd, *J* = 5.88, 9.63 Hz, 1H), 6.09 (d, *J* = 9.63 Hz, 1H), 4.30 (ddd, *J* = 2.63, 6.13, 8.38 Hz, 1H), 4.05 (dd, *J* = 2.5, 5.75 Hz, 1H), 3.59 (t, *J* = 6.63 Hz, 2H), 1.94–1.86 (m, 1H), 1.83–1.74 (m, 1H), 1.53–1.47 (m, 3H), 1.43–1.38 (m, 1H), 1.30 (br, s, 8H), 0.88 (s, 9H), 0.04 (s, 6H); ^13^C{^1^H} NMR (101 MHz, CDCl_3_) *δ* 164.2, 144.5, 122.9, 81.0, 63.3, 62.0, 32.8, 31.6, 30.0, 29.44, 29.38, 29.34, 26.0, 25.8, 24.9, 22.7, 18.4, 14.1, −5.2; HRMS (ESI): *m*/*z* calcd for C_19_H_37_O_4_Si [M + H]^+^ 357.2456, found 357.2451.

### (2*S*,3*S*)-2-(8-((*Tert*-Butyldimethylsilyl)oxy)octyl)-6-oxo-3,6-dihydro-2*H*-pyran-3-yl acetate (41)

In a 25 mL two-neck round-bottom flask, compound 2 (0.175 g, 0.478 mmol) in CH_2_Cl_2_ (4 mL) was added pyridine (0.0918 mL, 1.14 mmol), DMAP (0.0058 g, 0.0478 mmol), and Ac_2_O (0.067 mL, 0.717 mmol) sequentially and stirred at room temperature for 20 min. After completion of reaction, the reaction mixture was diluted with CH_2_Cl_2_ (5 mL) and quenched with 1 M HCl solution. The aqueous layer was extracted with CH_2_Cl_2_ (10 mL × 3), dried over Na_2_SO_4_, and filtered. The solvent was evaporated under reduced pressure, and the crude product was purified by silica gel column chromatography to afford the desired product 41 as an amorphous white solid (0.179 g, 94%). TLC: *R*_f_ = 0.5 (SiO_2_, 30% EtOAc : hexane). [*α*]^25^_D_ = +145.7 (*c* = 1.4, CHCl_3_). IR (CHCl_3_) 2928, 2855, 1735, 1633, 1463, 1372, 1251, 1225, 1096, 1022, 949, 834, 775 cm^−1^; ^1^H NMR (400 MHz, CDCl_3_) *δ* 6.96 (dd, *J* = 5.88, 9.63 Hz, 1H), 6.20 (d, *J* = 9.76 Hz, 1H), 5.17 (dd, *J* = 2.63, 5.88 Hz, 1H), 4.44 (ddd, *J* = 2.75, 5.0, 8.13 Hz, 1H), 3.59 (t, *J* = 6.59 Hz, 2H), 2.10 (s, 3H), 1.89–1.83 (m, 1H), 1.66–1.60 (m, 2H), 1.52–1.46 (m, 2H), 1.29 (br, s, 8H), 0.89 (s, 9H), 0.04 (s, 6H); ^13^C{^1^H} NMR (101 MHz, CDCl_3_) *δ* 170.3, 163.1, 140.4, 125.2, 79.0, 63.4, 63.2, 33.0, 30.2, 29.5, 29.43, 29.36, 26.1, 25.9, 25.0, 20.7, 18.5, −5.1; HRMS (ESI): *m*/*z* calcd for C_21_H_39_O_5_Si [M + H]^+^ 399.2561, found 399.2570.

### (*S*)-6-(8-((*Tert*-Butyldimethylsilyl)oxy)octyl)-3,6-dihydro-2*H*-pyran-2-one (S-5)

In a 20 mL two-neck round-bottom flask, solution of compound 41 (0.168 g, 0.421 mmol) in THF (6 mL) was added zinc powder (0.275 g, 4.21 mmol), and the reaction was stirred for 5 min at room temperature. Then, a saturated aqueous solution of NH_4_Cl (6 mL) was added, and the mixture was stirred for 4 h. After completion of the reaction, the reaction mixture was filtered over Celite and washed with EtOAc. The aqueous layer was then extracted with EtOAc (10 mL × 3), dried over Na_2_SO_4_, and filtered. The solvent was evaporated under reduced pressure, and the crude product was purified by silica gel column chromatography to afford the desired product S-5 as a yellow liquid (0.126 g, 88%). TLC: *R*_f_ = 0.6 (SiO_2_, 30% EtOAc : hexane). [*α*]^25^_D_ = +68.6 (*c* = 0.4, CHCl_3_). IR (CHCl_3_) 2927, 2855, 1744, 1463, 1383, 1254, 1223, 1155, 1097, 1006, 974, 835, 776, 704, 667 cm^−1^; ^1^H NMR (400 MHz, CDCl_3_) *δ* 5.83 (s, 2H), 4.99–4.97 (m, 1H), 3.59 (t, *J* = 6.63 Hz, 2H), 3.06–3.04 (m, 2H), 1.75–1.69 (m, 2H), 1.51–1.46 (m, 3H), 1.42–1.39 (m, 1H), 1.29 (br, s, 8H), 0.89 (s, 9H), 0.04 (s, 6H); ^13^C{^1^H} NMR (101 MHz, CDCl_3_) *δ* 169.3, 126.8, 121.6, 79.9, 63.4, 35.9, 33.0, 30.1, 29.6, 29.5, 29.4, 26.1, 25.9, 24.5, 18.5, 0.1, −5.1; HRMS (ESI): *m*/*z* calcd for C_19_H_37_O_3_Si [M + H]^+^ 341.2506, found 341.2507.

### (*S*)-6-(8-((*Tert*-Butyldimethylsilyl)oxy)octyl)-5,6-dihydro-2*H*-pyran-2-one (42)

In a 10 mL two-neck round-bottom flask, a solution of the compound S-5 (0.118 g, 0.346 mmol) in anhydrous THF (2 mL) was added DBU (0.103 mL, 0.692 mmol) and stirred for 2 h at room temperature. After completion of the reaction, the solvent was removed under reduced pressure, and the crude residue was purified on silica gel column chromatography to afford desired product 42 as a yellow liquid (0.1 g, 85%). TLC: *R*_f_ = 0.5 (SiO_2_, 30% EtOAc : hexane). [*α*]^25^_D_ = +178.8 (*c* = 0.2, CHCl_3_). IR (CHCl_3_) 2928, 2855, 1725, 1470, 1388, 1250, 1149, 1098, 1039, 1006, 959, 835, 816, 775, 723, 661 cm^−1^; ^1^H NMR (400 MHz, CDCl_3_) *δ* 6.83 (ddd, *J* = 3.5, 5.13, 9.63 Hz, 1H), 5.97 (td, *J* = 1.38, 9.76 Hz, 1H), 4.40–4.33 (m, 1H), 3.55 (t, *J* = 6.63 Hz, 2H), 2.31–2.26 (m, 2H), 1.80–1.71 (m, 1H), 1.63–1.56 (m, 1H), 1.47–1.44 (m, 3H), 1.38–1.34 (m, 1H), 1.25 (br, s, 8H), 0.85 (m, 9H), 0.00 (s, 6H); ^13^C{^1^H} NMR (101 MHz, CDCl_3_) *δ* 164.8, 145.1, 121.6, 78.2, 63.4, 35.0, 33.0, 29.6, 29.54, 29.46, 26.1, 25.9, 25.0, 18.5, −5.1; HRMS (ESI): *m*/*z* calcd for C_19_H_37_O_3_Si [M + H]^+^ 341.2506, found 341.2509.

### (*S*)-6-(8-Hydroxyoctyl)-5,6-dihydro-2*H*-pyran-2-one (S-6)

In a 25 mL two-neck round-bottom flask, compound 42 (0.093 g, 0.273 mmol) in anhydrous MeOH (15 mL) was cooled at 0 °C. To it, was added *p*-TSA.H_2_O (0.0051 g, 0.0273 mmol) dropwise, and the reaction mixture was allowed to warm to room temperature & stirred for 4 h. After completion of reaction, the solvent was evaporated under reduced pressure and the crude product was purified by silica gel column chromatography to afford the desired product S-6 as white solid (0.0563 g, 91%). TLC: *R*_f_ = 0.3 (SiO_2_, 50% EtOAc : hexane); [*α*]^25^_D_ = +64.3 (*c* = 0.3, CHCl_3_). IR (CHCl_3_) 3394, 2926, 2852, 1701, 1465, 1389, 1251, 1154, 1114, 1055, 1034, 959, 818, 724, 661 cm^−1^; ^1^H NMR (400 MHz, CDCl_3_) *δ* 6.90–6.85 (m, 1H), 6.02 (td, *J* = 2.0, 9.88 Hz, 1H), 4.45–4.38 (m, 1H), 3.64 (t, *J* = 6.63 Hz, 2H), 2.34–2.30 (m, 2H), 1.84–1.75 (m, 1H), 1.68–1.64 (m, 1H), 1.58–1.53 (m, 2H), 1.43–1.32 (m, 10H); ^13^C{^1^H} NMR (101 MHz, CDCl_3_) *δ* 164.8, 145.2, 121.6, 78.1, 63.2, 35.0, 32.9, 29.55, 29.51, 29.4, 29.39, 25.8, 24.9; HRMS (ESI): *m*/*z* calcd for C_13_H_23_O_3_ [M + H]^+^ 227.1642, found 227.1638.

### (*S*)-8-(6-Oxo-3,6-dihydro-2*H*-pyran-2-yl)octanal (43)

In a 50 mL two-neck round-bottom flask, pyran substituted alcohol S-6 (0.048 g, 0.212 mmol) in anhydrous CH_2_Cl_2_ (20 mL) was cooled at 0 °C. To it was added pyridinium chlorochromate (PCC) (0.054 g, 0.254 mmol) was stirred at room temperature for 4 h. After completion of the reaction, it was quenched with a 1 : 1 ratio of a saturated aqueous solution of NaHCO_3_ and Na_2_S_2_O_3_, and the aqueous layer was extracted with CH_2_Cl_2_ (30 mL × 3). Then, the combined organic layers were washed with brine, dried over Na_2_SO_4_, filtered, and the solvent was evaporated under reduced pressure. The resulting crude product was purified by silica gel column chromatography to afford the desired product 43 as a pale-yellow solid (0.041 g, 85%). TLC: *R*_f_ = 0.4 (SiO_2_, 40% EtOAc : hexane). [*α*]^25^_D_ = +97.9 (*c* = 0.5, CHCl_3_). IR (CHCl_3_) 2924, 2854, 2719, 1714, 1463, 1388, 1249, 1144, 1035, 958, 816, 724, 661 cm^−1^; ^1^H NMR (400 MHz, CDCl_3_) *δ* 9.76 (t, *J* = 1.75 Hz, 1H), 6.90–6.85 (m, 1H), 6.02 (td, *J* = 2, 9.88 Hz, 1H), 4.45–4.38 (m, 1H), 2.43 (dt, *J* = 1.75, 7.38 Hz, 2H), 2.34–2.31 (m, 2H), 1.83–1.75 (m, 1H), 1.68–1.61 (m, 3H), 1.53–1.50 (m, 1H), 1.43–1.37 (m, 1H), 1.33 (br, s, 6H); ^13^C{^1^H} NMR (101 MHz, CDCl_3_) *δ* 203.0, 164.0, 145.1, 121.6, 78.1, 44.0, 35.0, 29.6, 29.32, 29.27, 29.15, 24.9, 22.1; HRMS (ESI): *m*/*z* calcd for C_13_H_21_O_3_ [M + H]^+^ 225.1485, found 225.1481.

### Passifetilactone A (1)

In a 10 mL two-neck round-bottom flask, a slurry of Wittig salt 24 (0.047 g, 0.0823 mmol) in anhydrous THF (2 mL) was cooled to −78 °C, and KHMDS (0.0123 mL, 0.0123 mmol, 1.0 M in THF) was added. The reaction mixture was allowed to warm to room temperature over 1 h, then re-cooled to −78 °C. A solution of the aldehyde 43 (0.015 g, 0.0823 mmol) in THF (1 mL) was added dropwise, and the mixture was stirred for 1 h at −78 °C followed by 1 h at room temperature. After completion, the reaction mixture was quenched by saturated aqueous solution of NH_4_Cl, and the aqueous layer was extracted with EtOAc (20 mL × 3), dried over Na_2_SO_4_, and filtered. The solvent was evaporated under reduced pressure, and the crude product was purified by column chromatography using neutral alumina oxide to afford Passifetilactone A (1) as a white solid (0.014 g, 55% yield). TLC: *R*_f_ = 0.5 (SiO_2_, 30% EtOAc : hexane). [*α*]^25^_D_ = +0.38 (*c* = 0.7, MeOH). IR (CHCl_3_) 2924, 2854, 1691, 1636, 1462, 1377, 1226, 1000, 962, 830, 724 cm^−1^; ^1^H NMR (400 MHz, CDCl_3_) *δ* 6.89–6.85 (m, 1H), 6.02 (td, *J* = 1.5, 9.88 Hz, 1H), 5.36–5.33 (m, 2H), 4.45–4.38 (m, 1H), 2.34–2.30 (m, 2H), 2.02–1.99 (m, 4H), 1.8–1.75 (m, 1H), 1.63–1.61 (m, 2H), 1.43–1.40 (m, 1H), 1.26 (br, s, 16H), 0.88 (t, *J* = 7.0 Hz 3H); ^13^C{^1^H} NMR (101 MHz, CDCl_3_) *δ* 164.8, 145.1, 130.2, 129.9, 121.6, 78.2, 35.0, 32.1, 31.9, 29.9, 29.55, 29.51, 29.49, 29.39, 29.33, 29.1, 27.4, 27.3, 25.0, 22.8, 14.3; HRMS (ESI): *m*/*z* calcd for C_20_H_35_O_2_ [M + H]^+^ 307.2632, found 307.2631.

## Author contributions

R. K. conceived the project and provided overall direction for the research. A. K. V. and D. R. J conducted synthetic experiments, analyzed the data, and drafted the SI. All authors contributed to reviewing and providing comments on the manuscript and the SI.

## Conflicts of interest

There are no conflicts to declare.

## Supplementary Material

RA-015-D5RA06982C-s001

## Data Availability

Supplementary information (SI): ^1^H and ^13^C NMR spectra for both previously known and newly synthesized compounds, and HPLC data for selected compounds. See DOI: https://doi.org/10.1039/d5ra06982c.
