# Supplementary material for: Achmatowicz rearrangement-enabled unified total syntheses of (+)-passifetilactones A–C
Source: RSC Adv. 2025 Oct 21;15(47):39919–30. doi: 10.1039/d5ra06982c (PMC12538396; doi:10.1039/d5ra06982c)
Supplement: RA-015-D5RA06982C-s001 [file RA-015-D5RA06982C-s001.pdf]

Supplementary Information (SI) for RSC Advances.
This journal is © The Royal Society of Chemistry 2025

Supplementary Information

Achmatowicz rearrangement-enabled unified total syntheses of (+)-passifetilactones A−C

Aman Kumar Verma,[a,b] Dharmaraju Jeddi[a] and Ravindar Kontham*,[a,b]

[a]*Organic Chemistry Division, CSIR-National Chemical Laboratory, Dr. Homi Bhabha Road, Pune - 411008, India.*

[b]*Academy of Scientific and Industrial Research (AcSIR), Ghaziabad – 201002, India.*

*Email: k.ravindar@ncl.res.in, konthamravindar@gmail.com

Table of Contents

S. No	Contents	Page No
1	Table S1: Overall yield comparison with earlier reports.	S2
2	^{1}H, ^{13}C NMR spectra and HPLC chromatograms	S3-S76

Table S1: Overall yield comparison with earlier reports.

Work	Compound	Natural product /Enantiomer/ Epimer	Total steps	Overall Yield (%)
By López-Mendoza and Sartillo-Piscil[1]	(+)-passifetilactone A	Not synthesized	–	–
	(–)-passifetilactone B	Enantiomer	8	5.8
	(–)-passifetilactone C	Enantiomer	8	4.8
By Rodney A. Fernandes[2]	(+)-passifetilactone A	Natural product	8	37.4
	(–)-passifetilactone B	Epimer	10	3.5
	(+)-passifetilactone C	Natural product	4	60
This Work	(+)-passifetilactone A	Natural product	13	12
	(+)-passifetilactone B	Natural product	5	54
	(+)-passifetilactone C	Natural product	8	37

References:

1. J. Bautista-Nava, L. F. Porras-Santos, L. Quintero, J. A. Pérez-Bautista, P. López-Mendoza and F. Sartillo-Piscil, *J. Org. Chem*, 2025, **90**, 6251–6260.
2. S. B. Khandekar, N. R. Barnala and R. A. Fernandes, *Org. Biomol. Chem*, 2025, **23**, 6637–6643.

^{1}H, ^{13}C NMR spectra and HPLC chromatograms

^{1}H NMR spectrum of 1-(Furan-2-yl)hexadecan-1-ol (16):

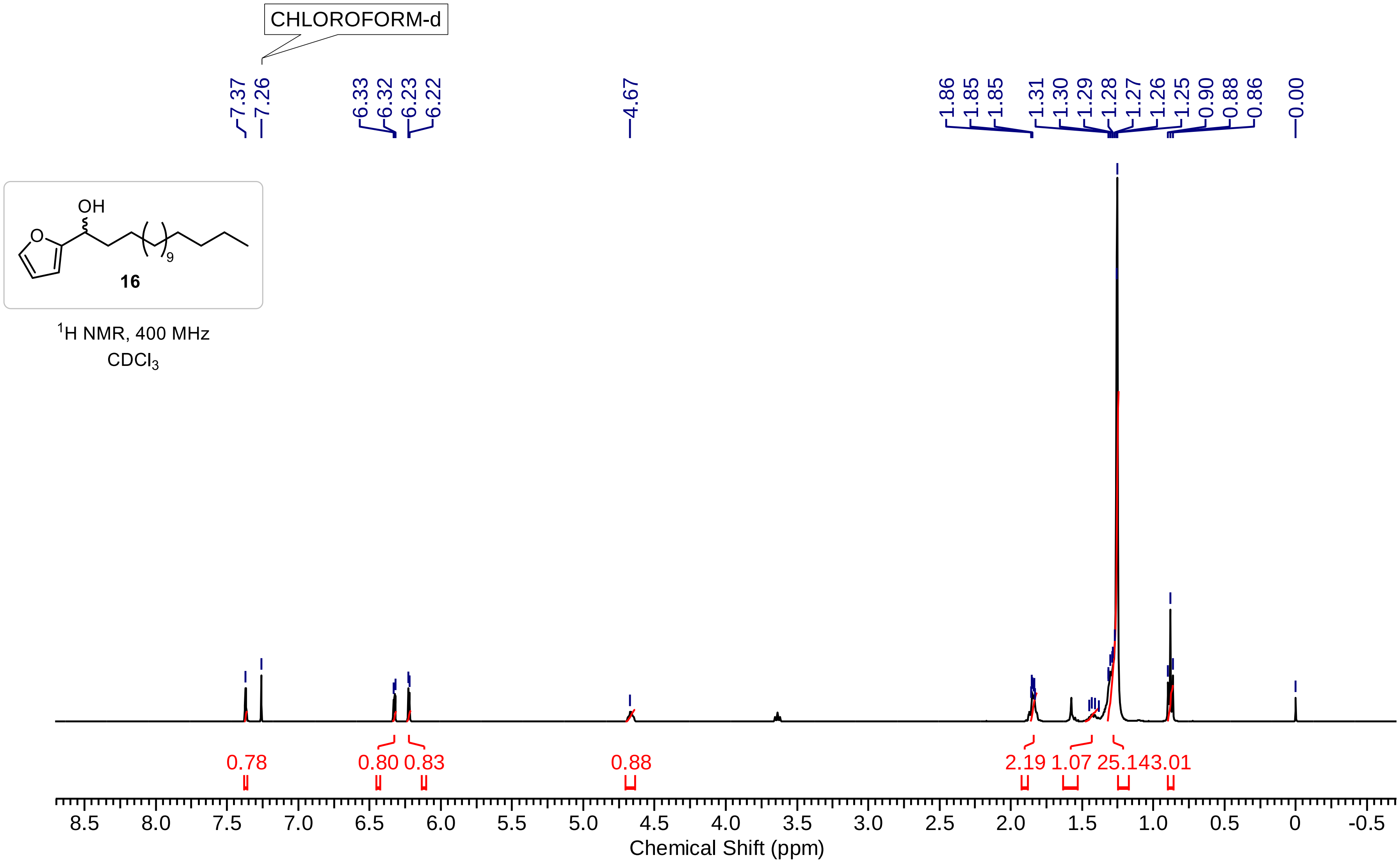

^{1}H NMR, 400 MHz
$CDCl_3$

$^{13}C\{^1H\}$ NMR spectrum of 1-(Furan-2-yl)hexadecan-1-ol (16):

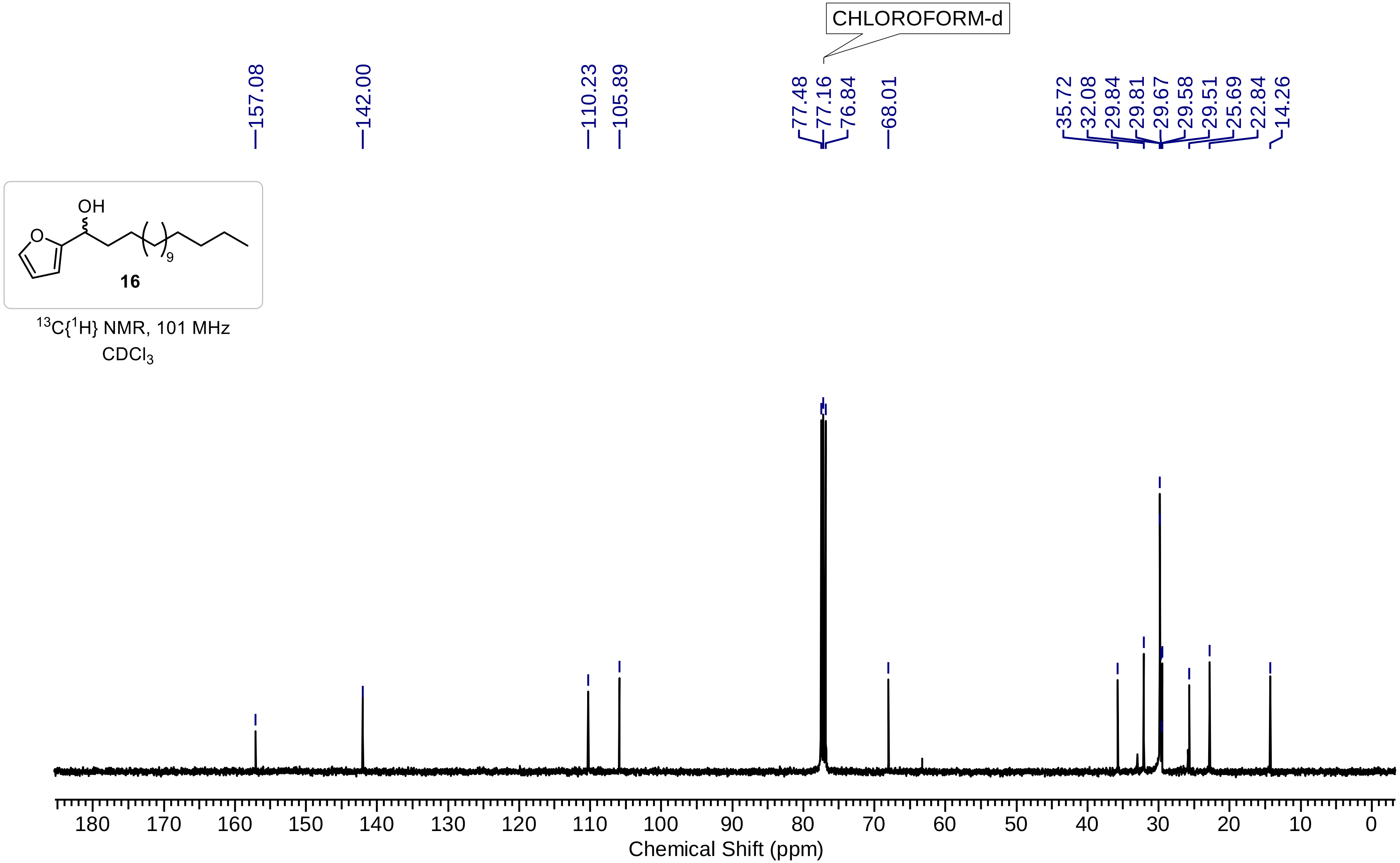

^{1}H NMR spectrum of 1-(Furan-2-yl)hexadecan-1-one (17a):

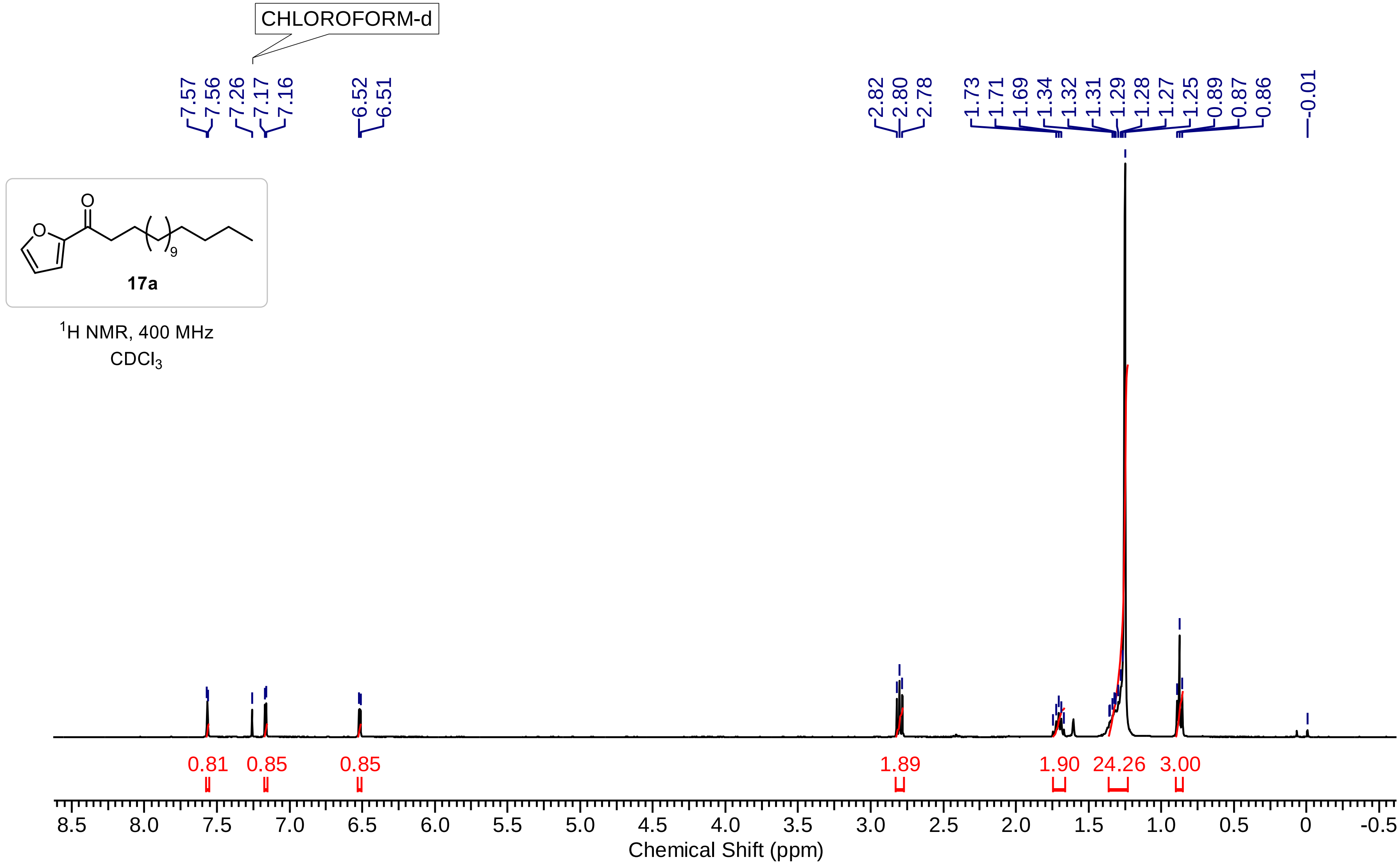

$^{13}C\{^1H\}$ NMR spectrum of 1-(Furan-2-yl)hexadecan-1-one (17a):

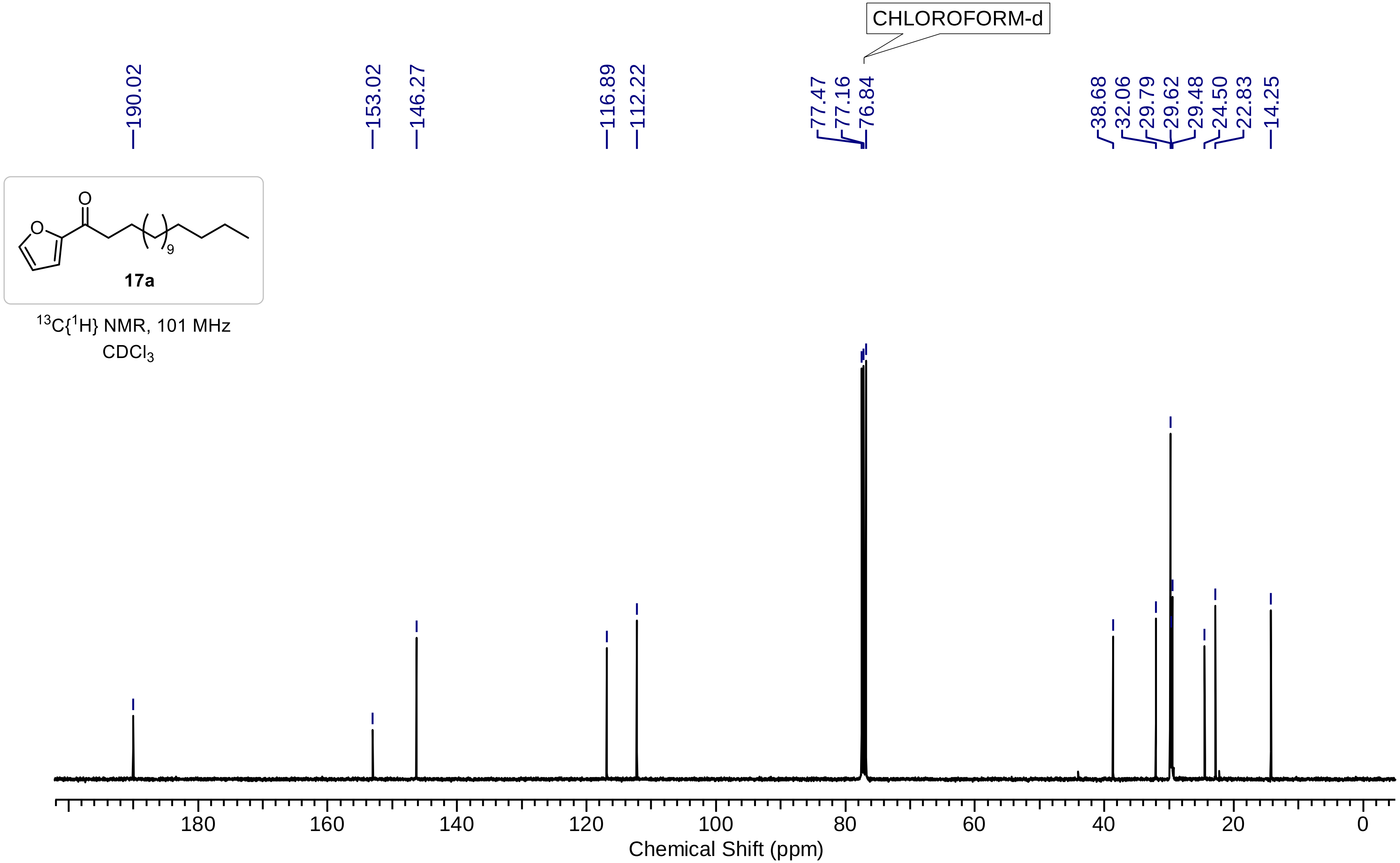

^{1}H NMR spectrum of (*S*)-1-(Furan-2-yl)hexadecan-1-ol (18a):

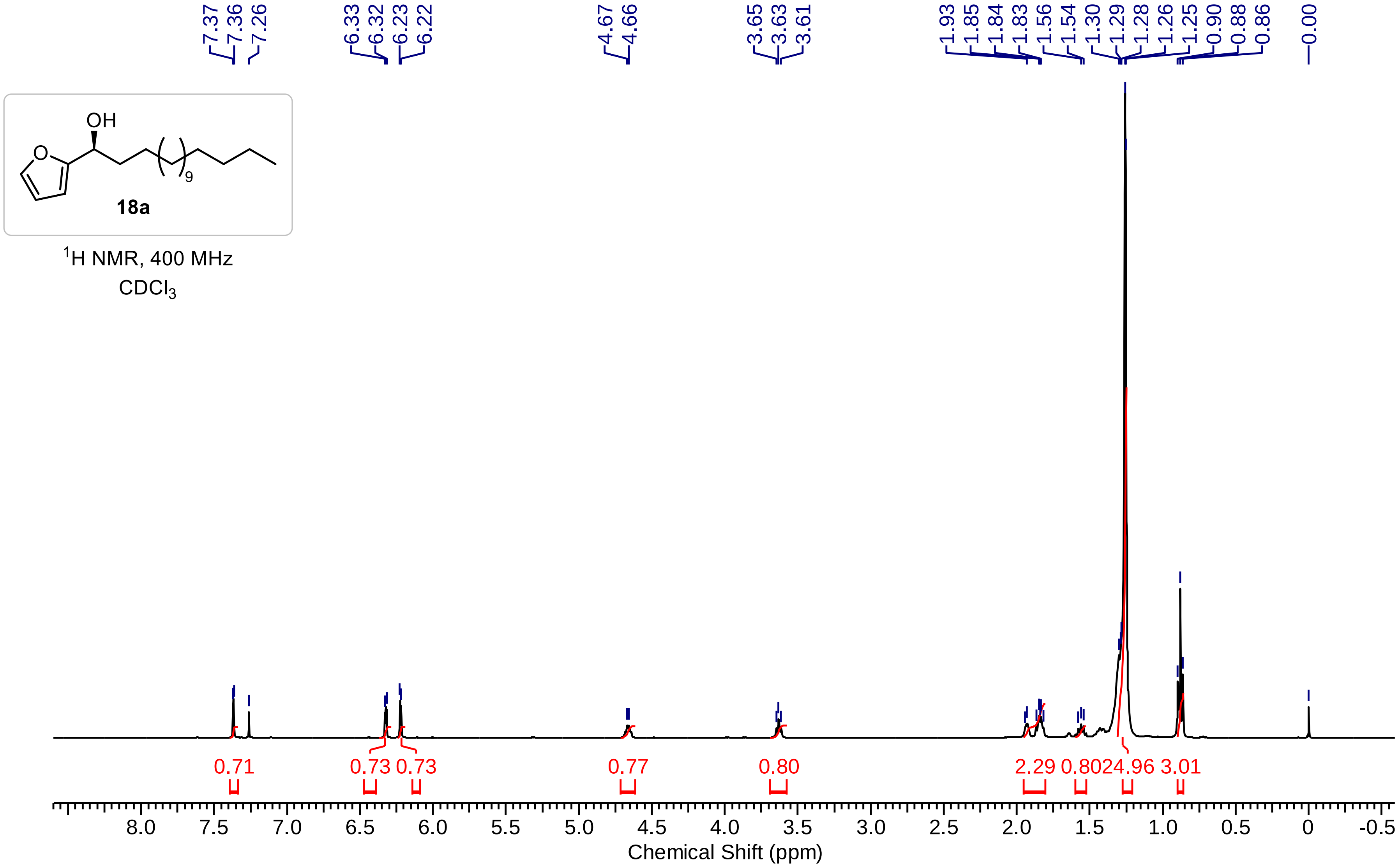

$^{13}C\{^1H\}$ NMR spectrum of (*S*)-1-(Furan-2-yl)hexadecan-1-ol (18a):

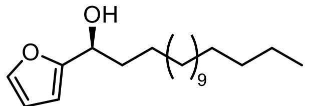

18a

$^{13}C\{^1H\}$ NMR, 101 MHz
$CDCl_3$

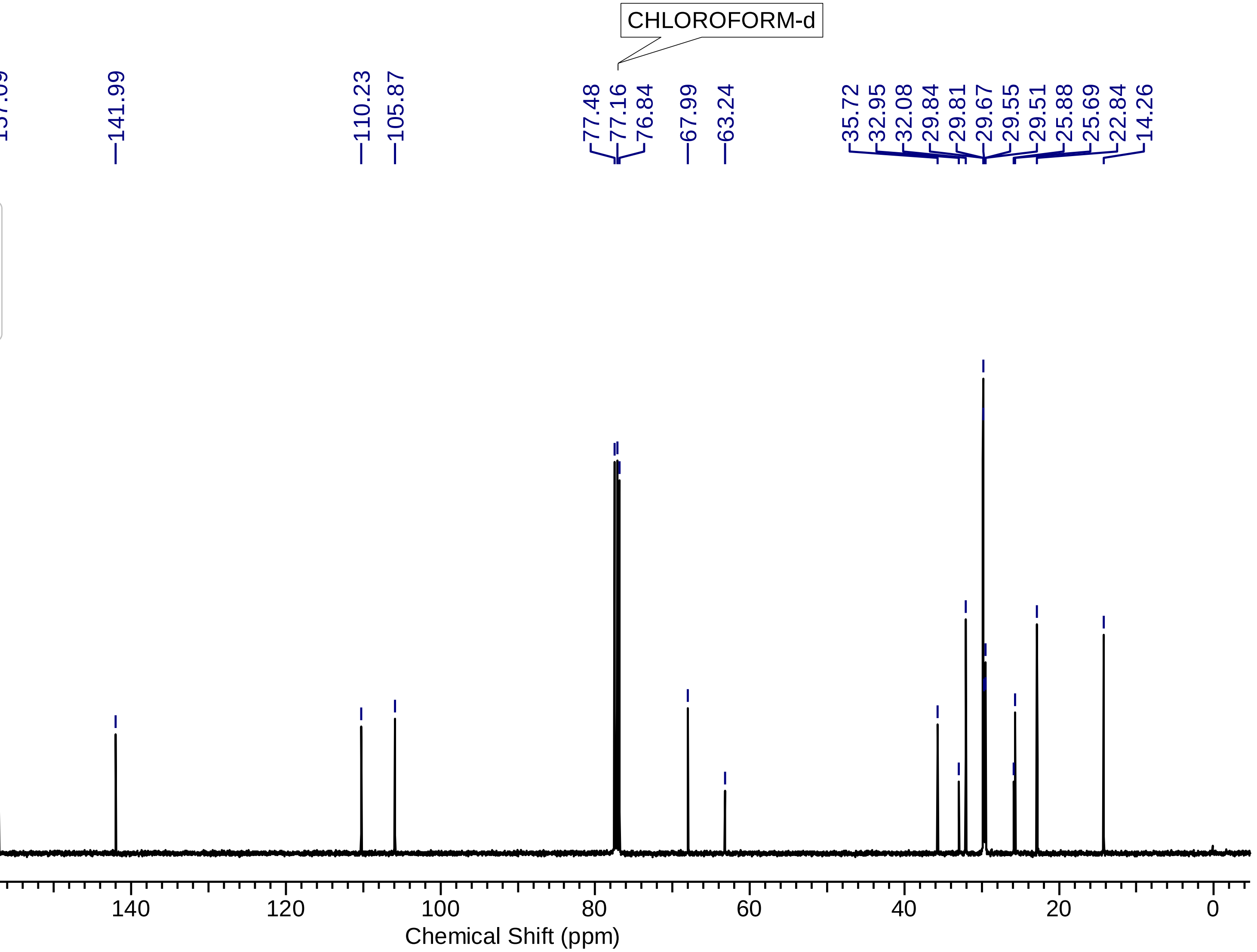

HPLC spectrum of (±)-1-(Furan-2-yl)hexadecan-1-ol (16):

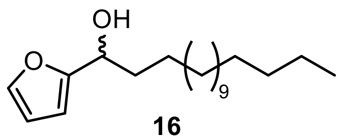

16

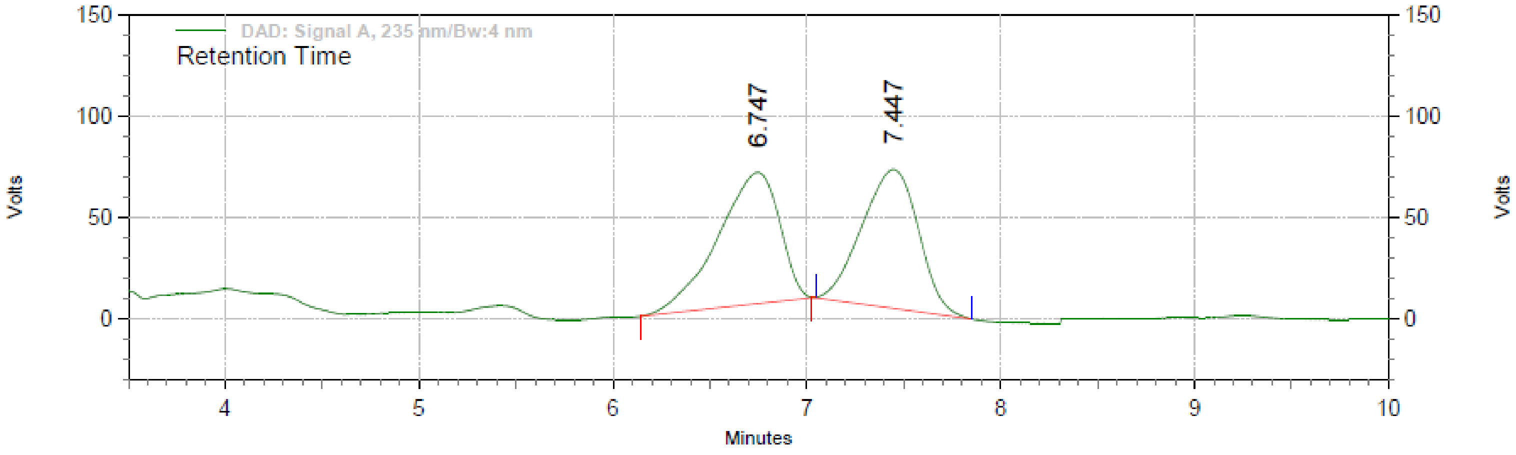

DAD: Signal A,
235 nm/Bw:4 nm
Results

Retention Time	Area	Area %	Height	Height %
6.747	3016014	50.82	135922	48.58
7.447	2918742	49.18	143879	51.42
Totals	5934756	100.00	279801	100.00

HPLC spectrum of (*S*)-1-(Furan-2-yl)hexadecan-1-ol (18a):

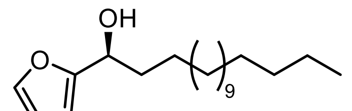

18a

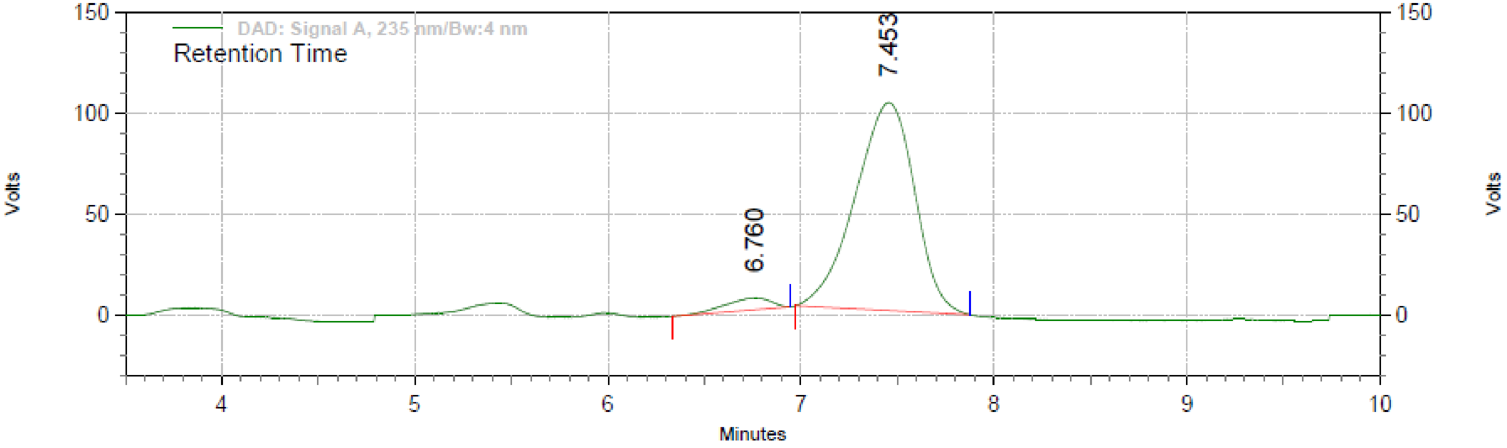

DAD: Signal A,
235 nm/Bw:4 nm
Results

Retention Time	Area	Area %	Height	Height %
6.760	203974	4.21	12334	5.40
7.453	4636874	95.79	216240	94.60
Totals	4840848	100.00	228574	100.00

^{1}H NMR spectrum of (2*S*)-6-Hydroxy-2-pentadecyl-2H-pyran-3(6H)-one (19a):

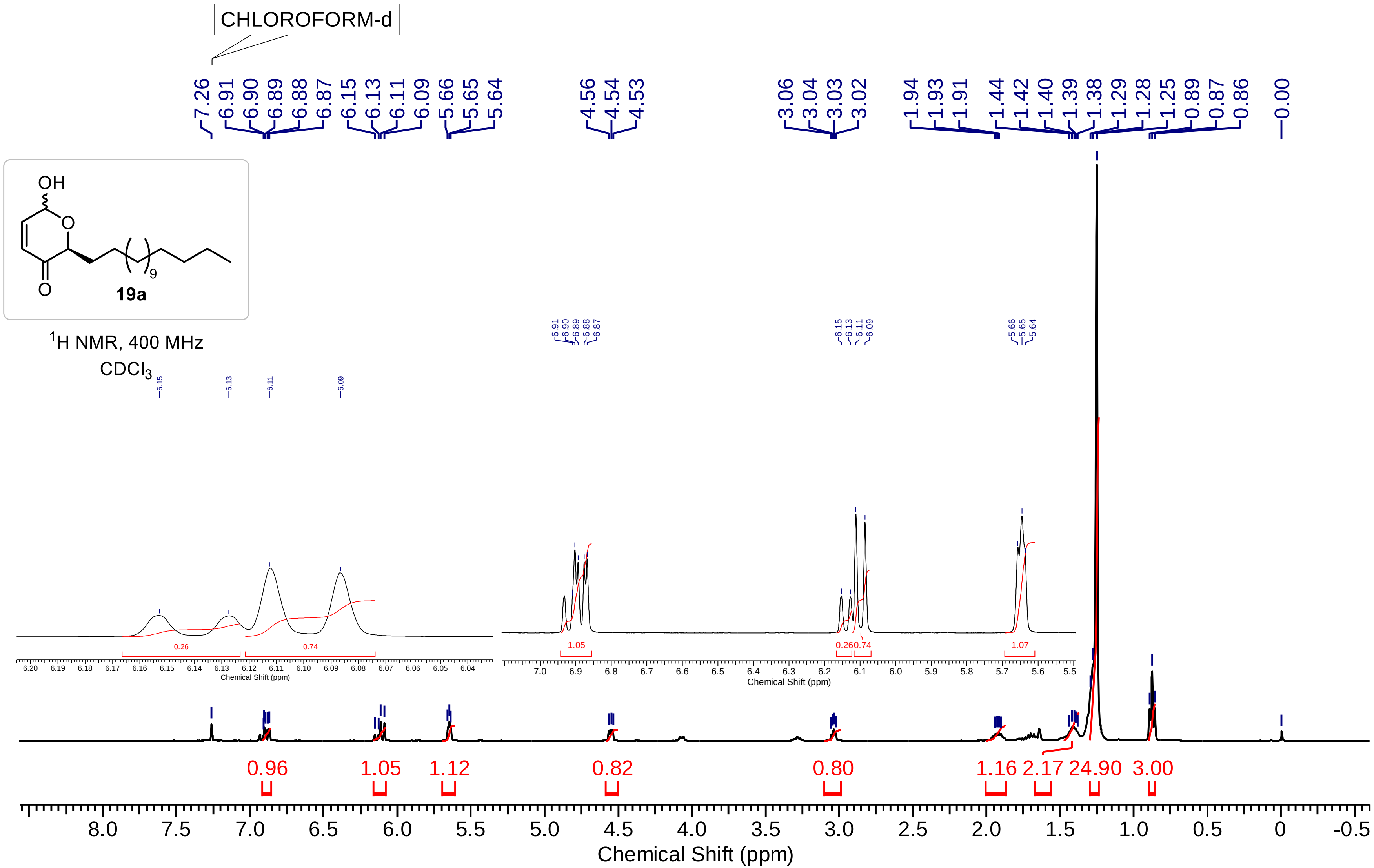

$^{13}C\{^1H\}$ NMR spectrum of (2*S*)-6-Hydroxy-2-pentadecyl-2H-pyran-3(6H)-one (19a):

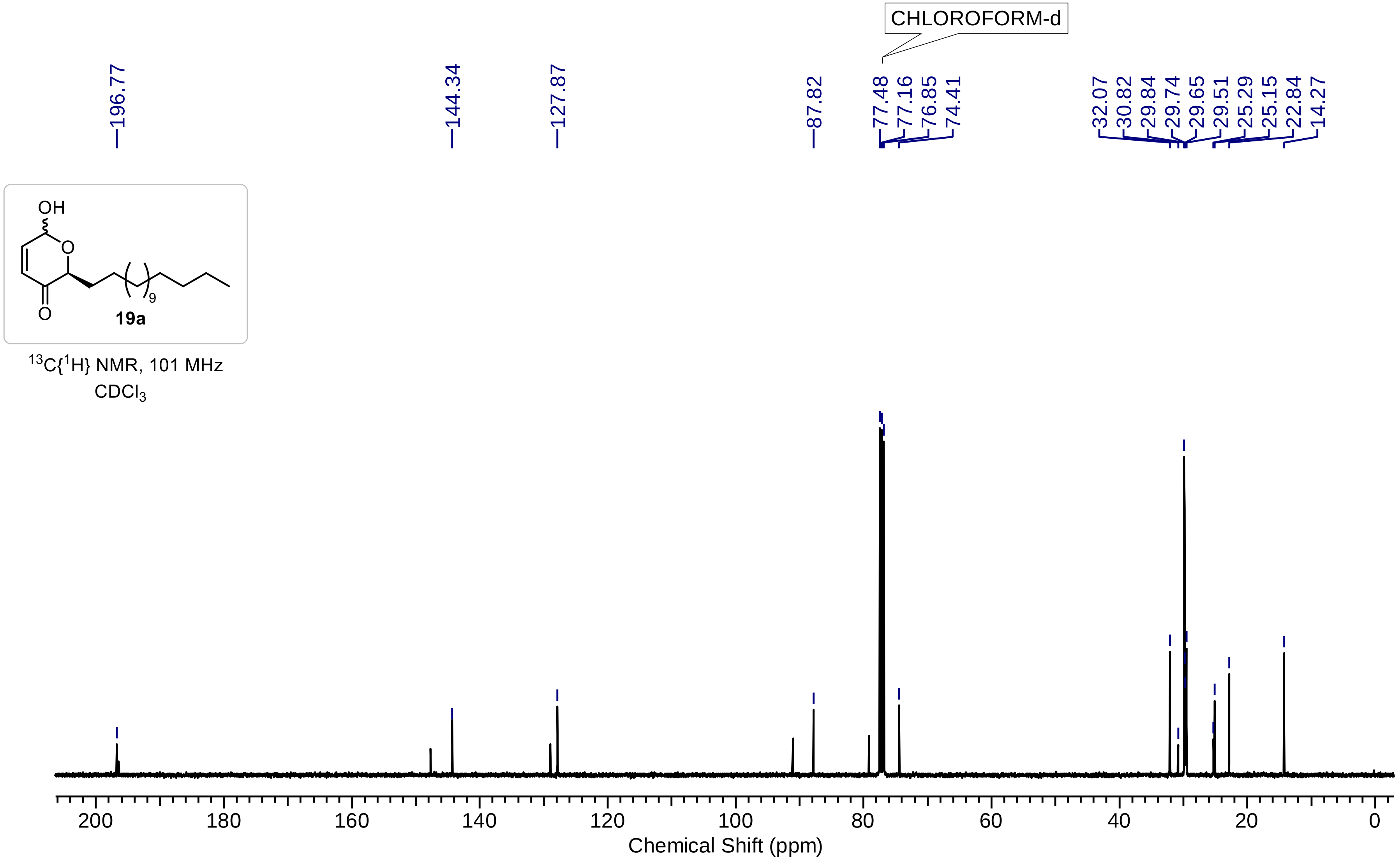

^{1}H NMR spectrum of Passifetilactone B (2):

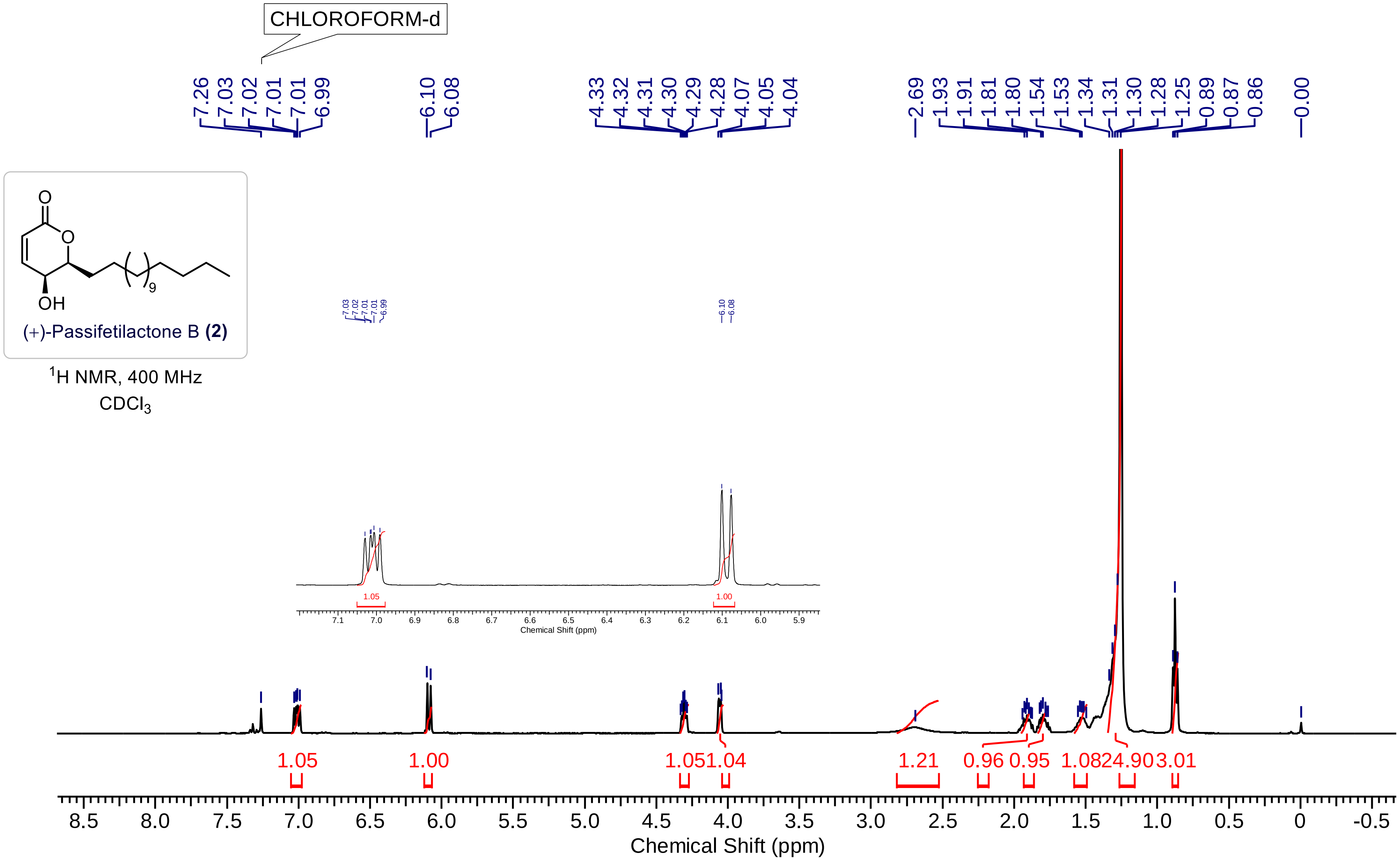

(+)-Passifetilactone B **(2)**

^{1}H NMR, 400 MHz
$CDCl_3$

^{13}C{^{1}H} NMR spectrum of Passifetilactone B (2):

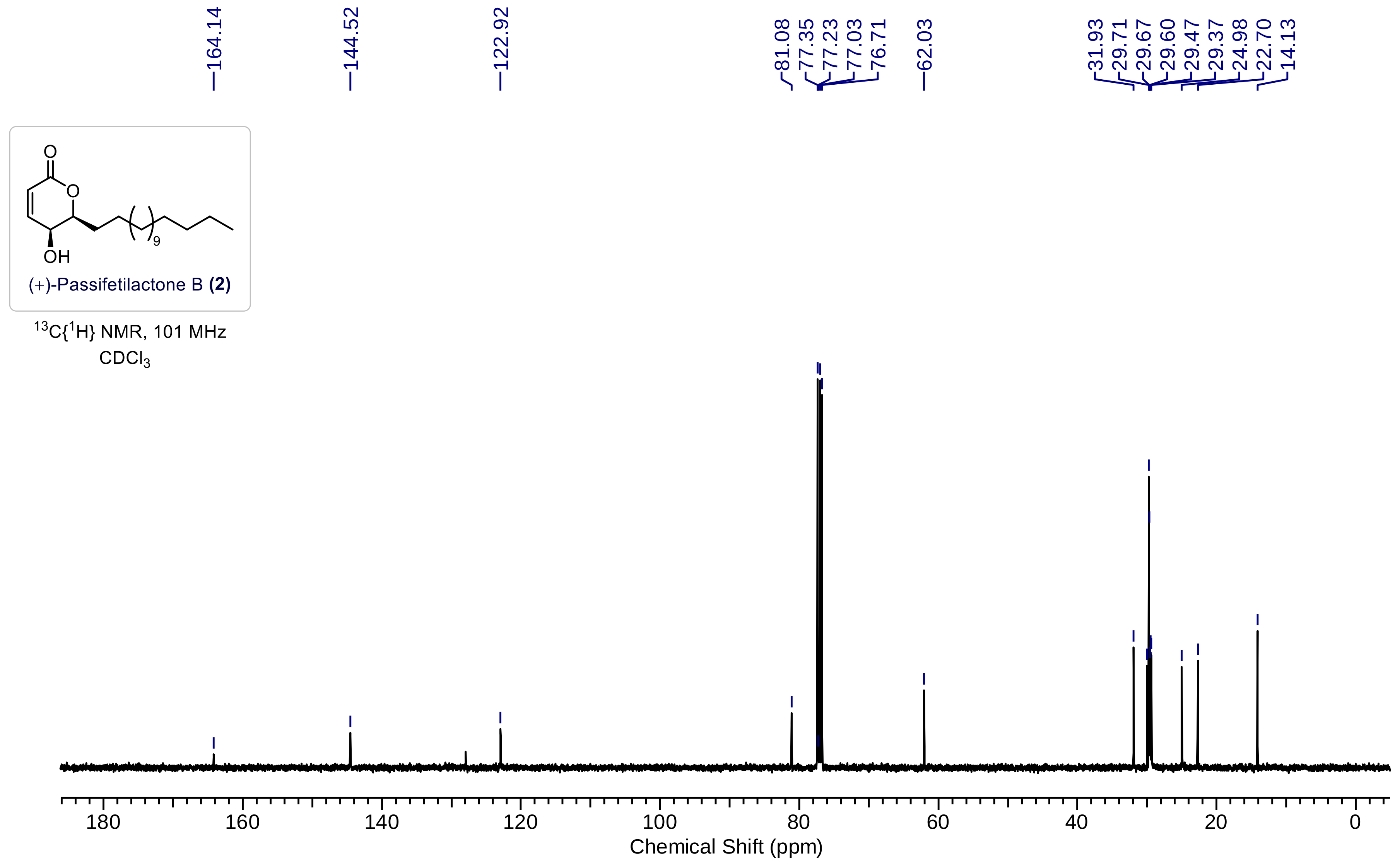

^{1}H NMR spectrum of (2*S*,3*S*)-6-Oxo-2-pentadecyl-3,6-dihydro-2H-pyran-3-yl acetate (20):

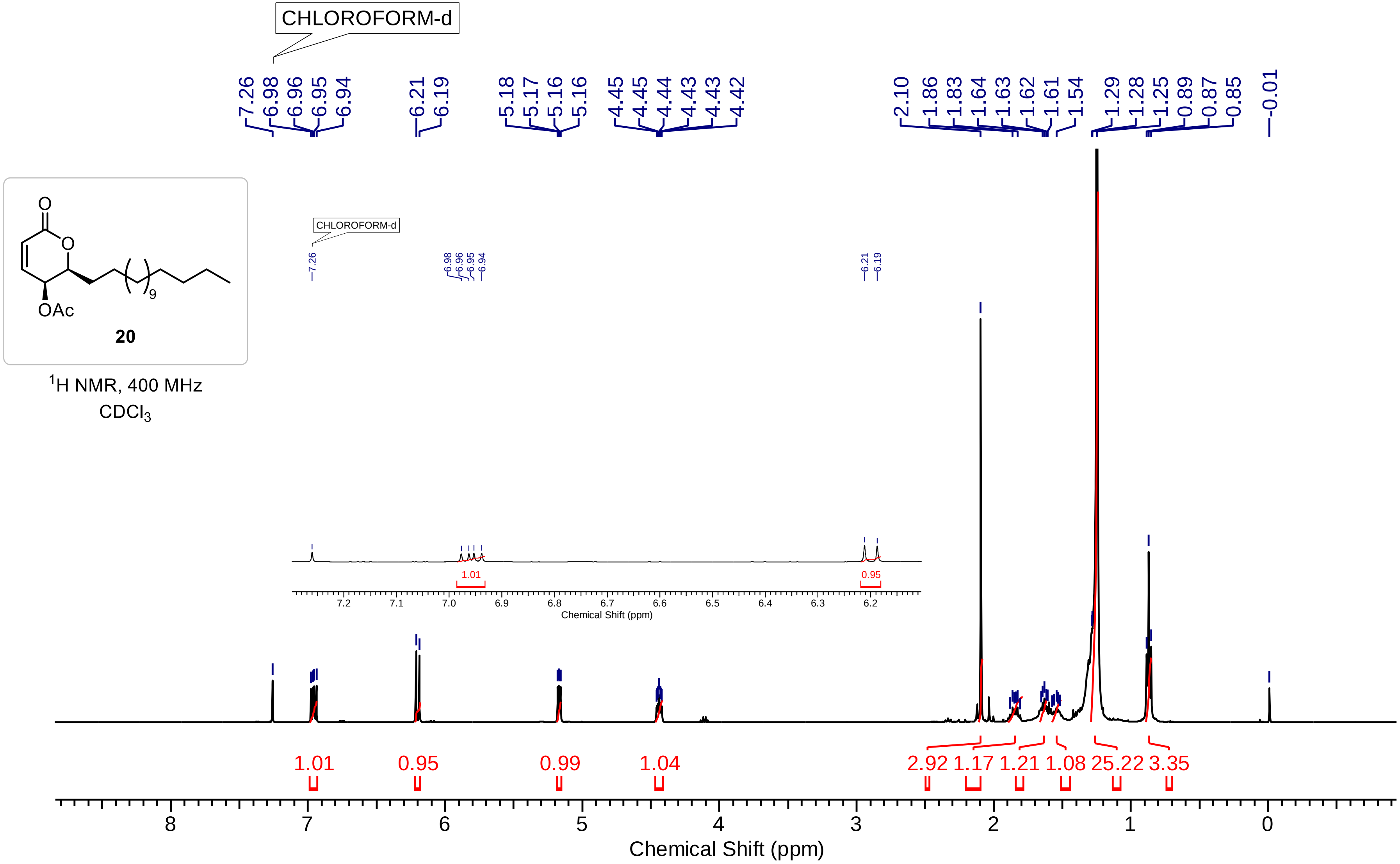

$^{13}C\{^1H\}$ NMR spectrum of (2*S*,3*S*)-6-Oxo-2-pentadecyl-3,6-dihydro-2H-pyran-3-yl acetate (20):

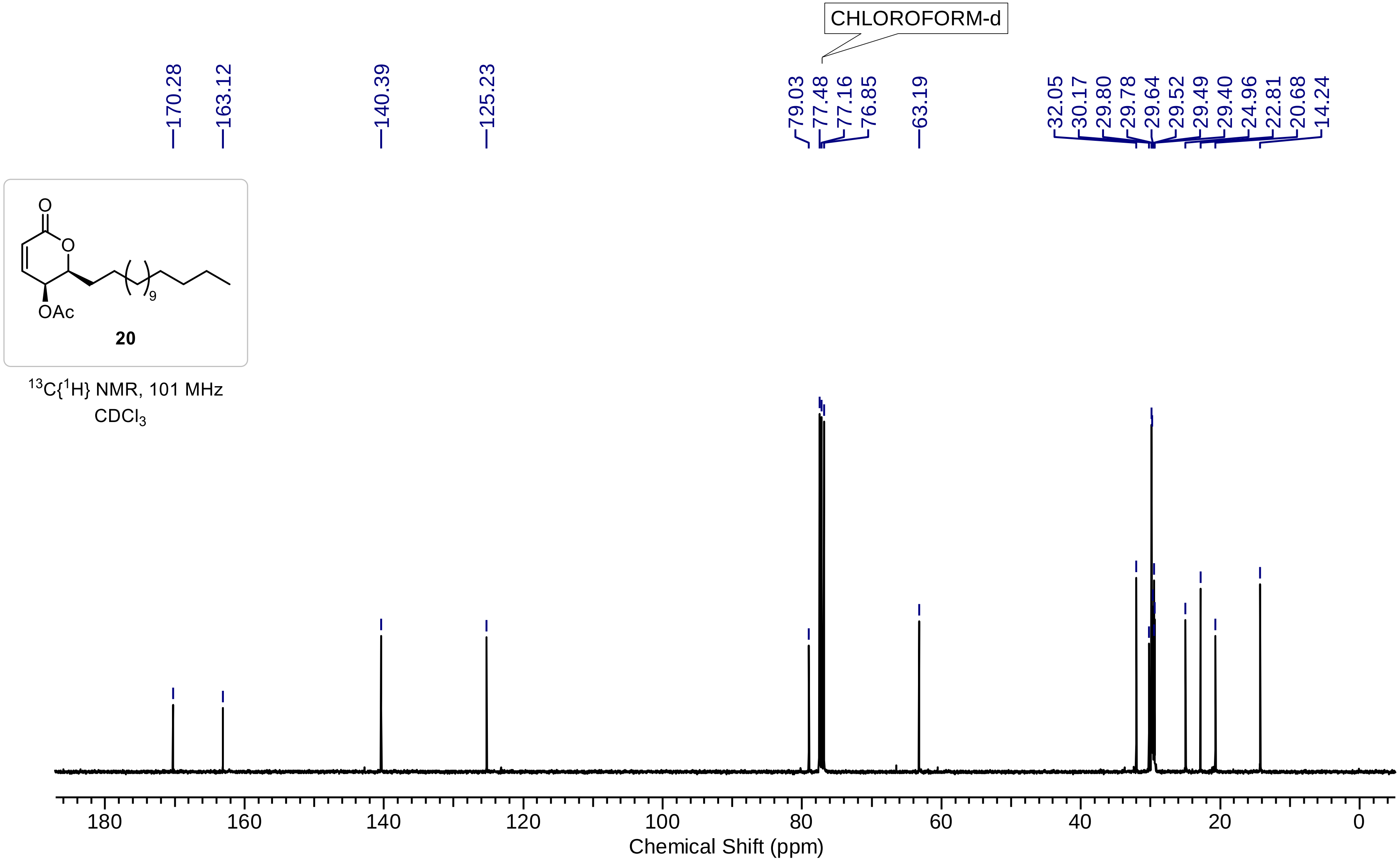

^{1}H NMR spectrum of 6-Pentadecyl-2H-pyran-2-one (20a):

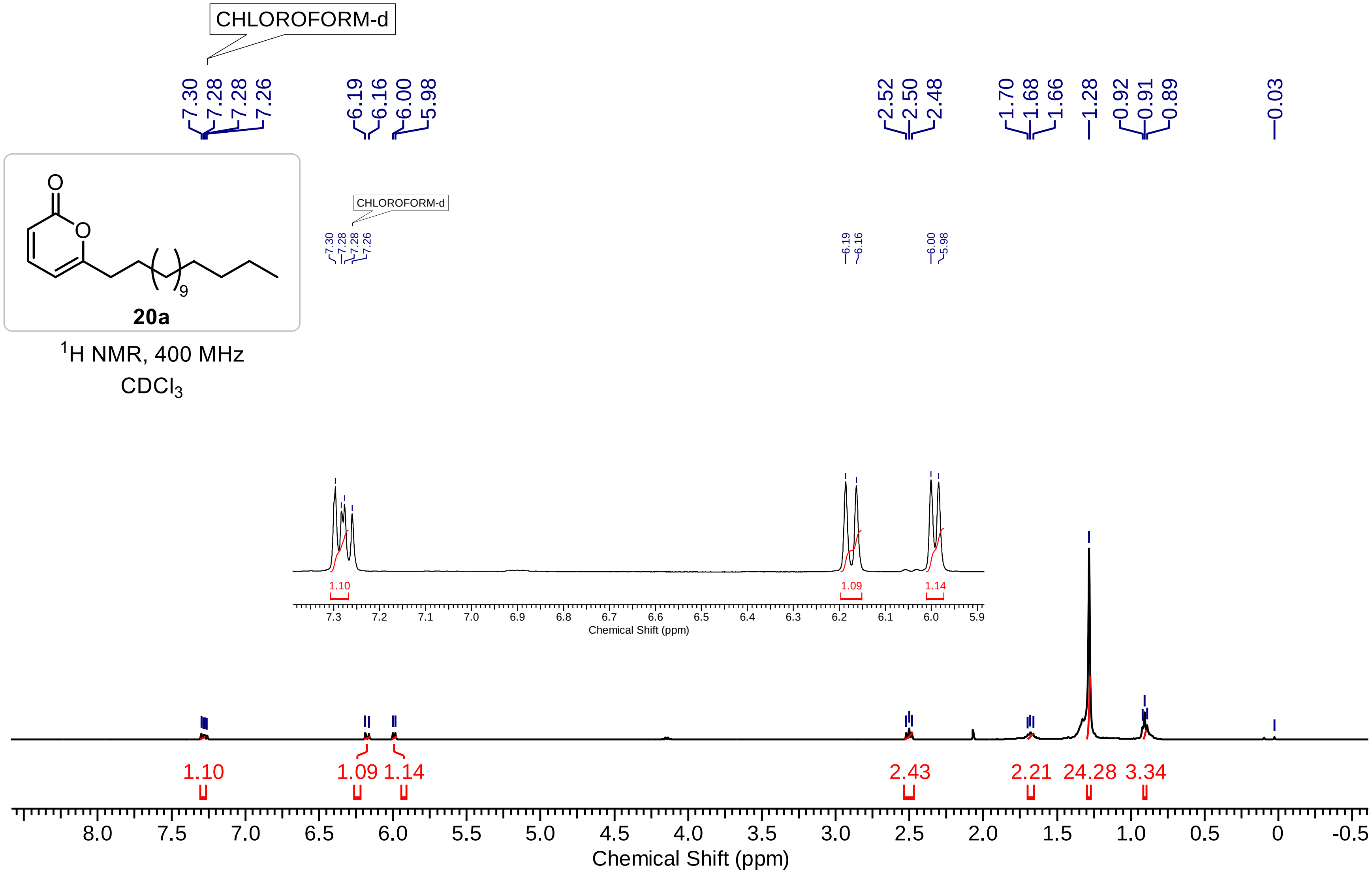

$^{13}C\{^{1}H\}$ NMR spectrum of 6-Pentadecyl-2H-pyran-2-one (20a):

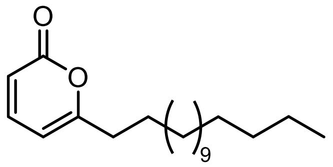

20a

$^{13}C\{^{1}H\}$ NMR, 101 MHz

$CDCl_3$

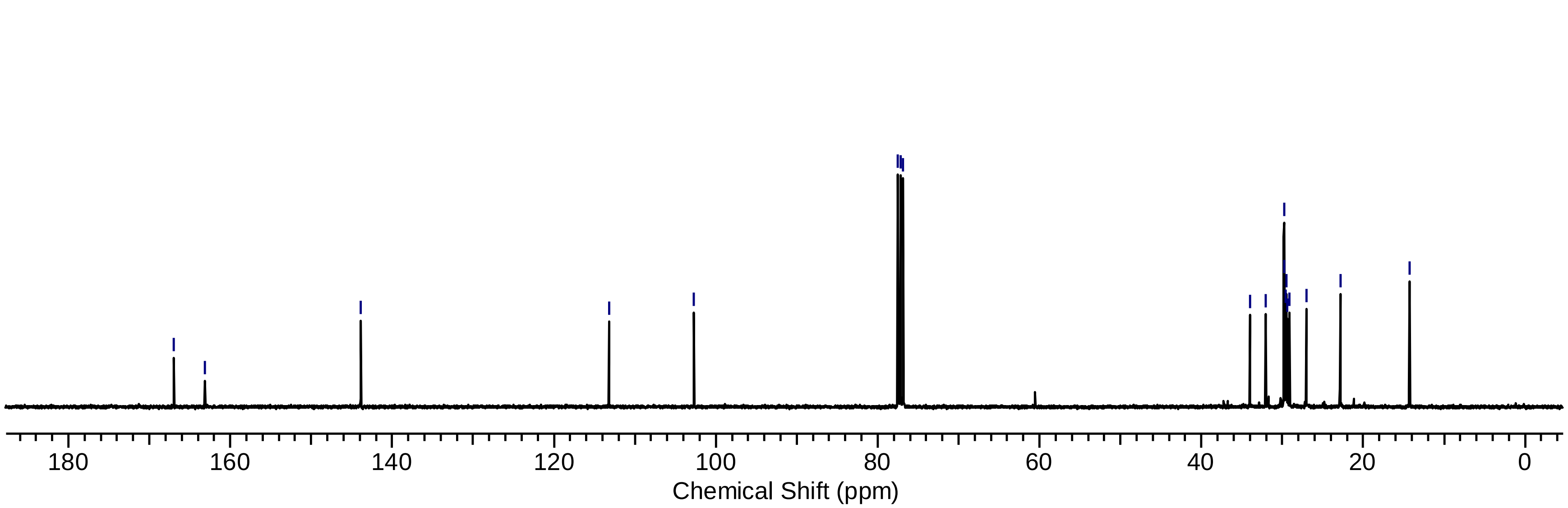

^{1}H NMR spectrum of (*S*)-6-Pentadecyl-3,6-dihydro-2H-pyran-2-one (21):

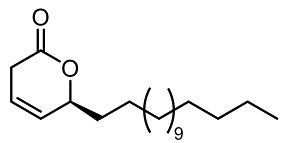

21

^{1}H NMR, 400 MHz
$CDCl_3$

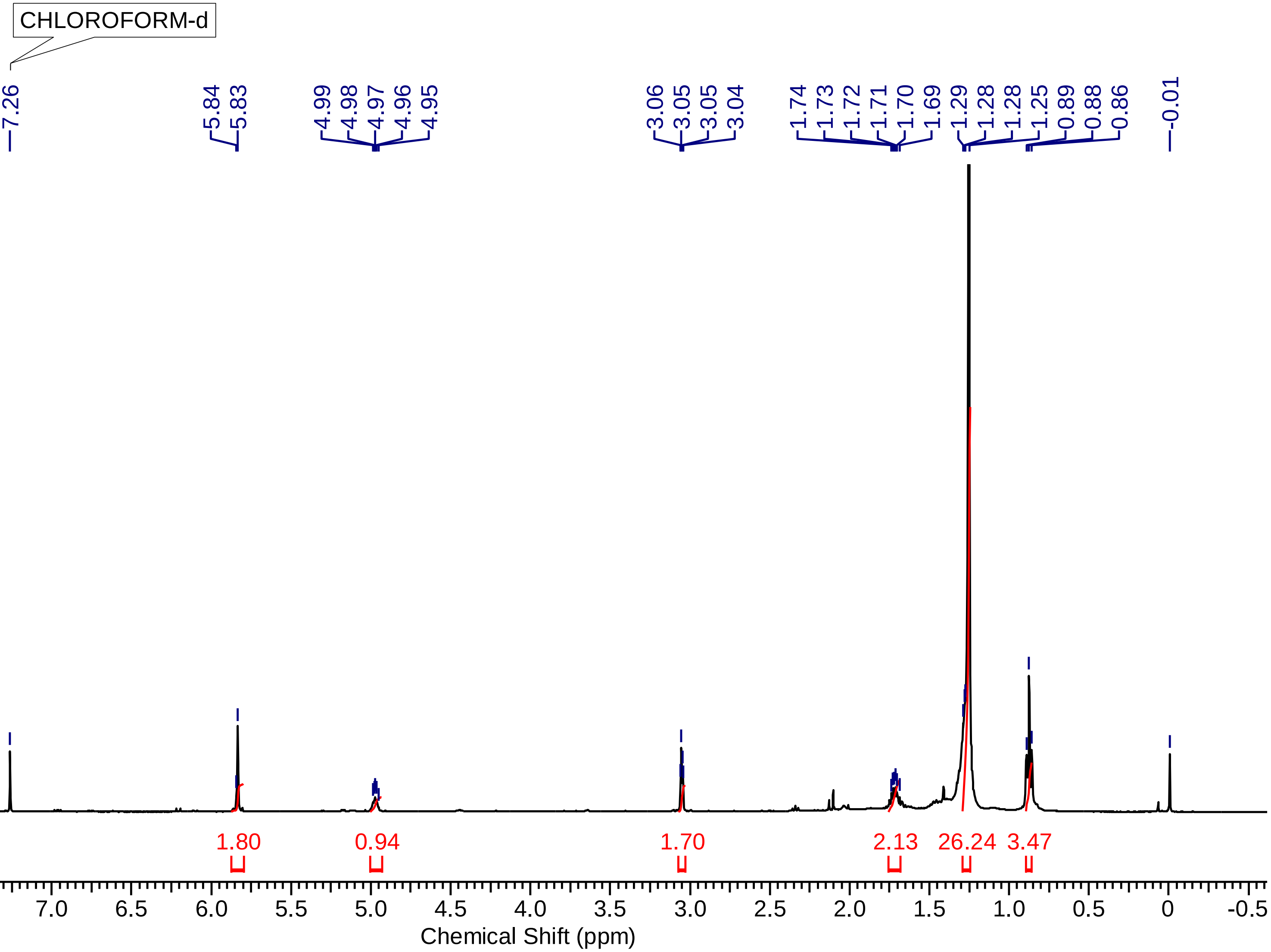

^{13}C{^{1}H} NMR spectrum of (*S*)-6-Pentadecyl-3,6-dihydro-2H-pyran-2-one (21):

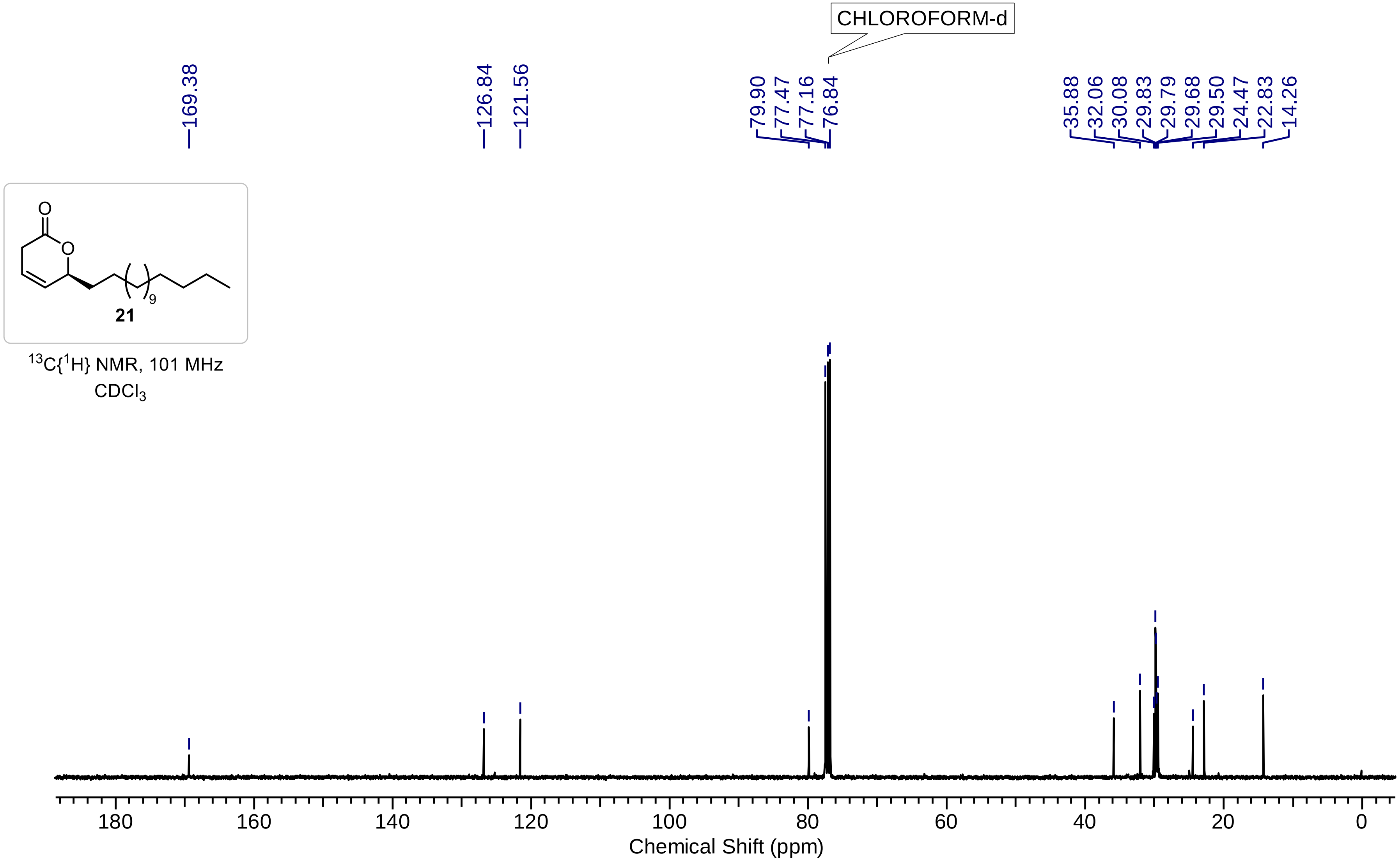

^{1}H NMR spectrum of Passifetilactone C (3):

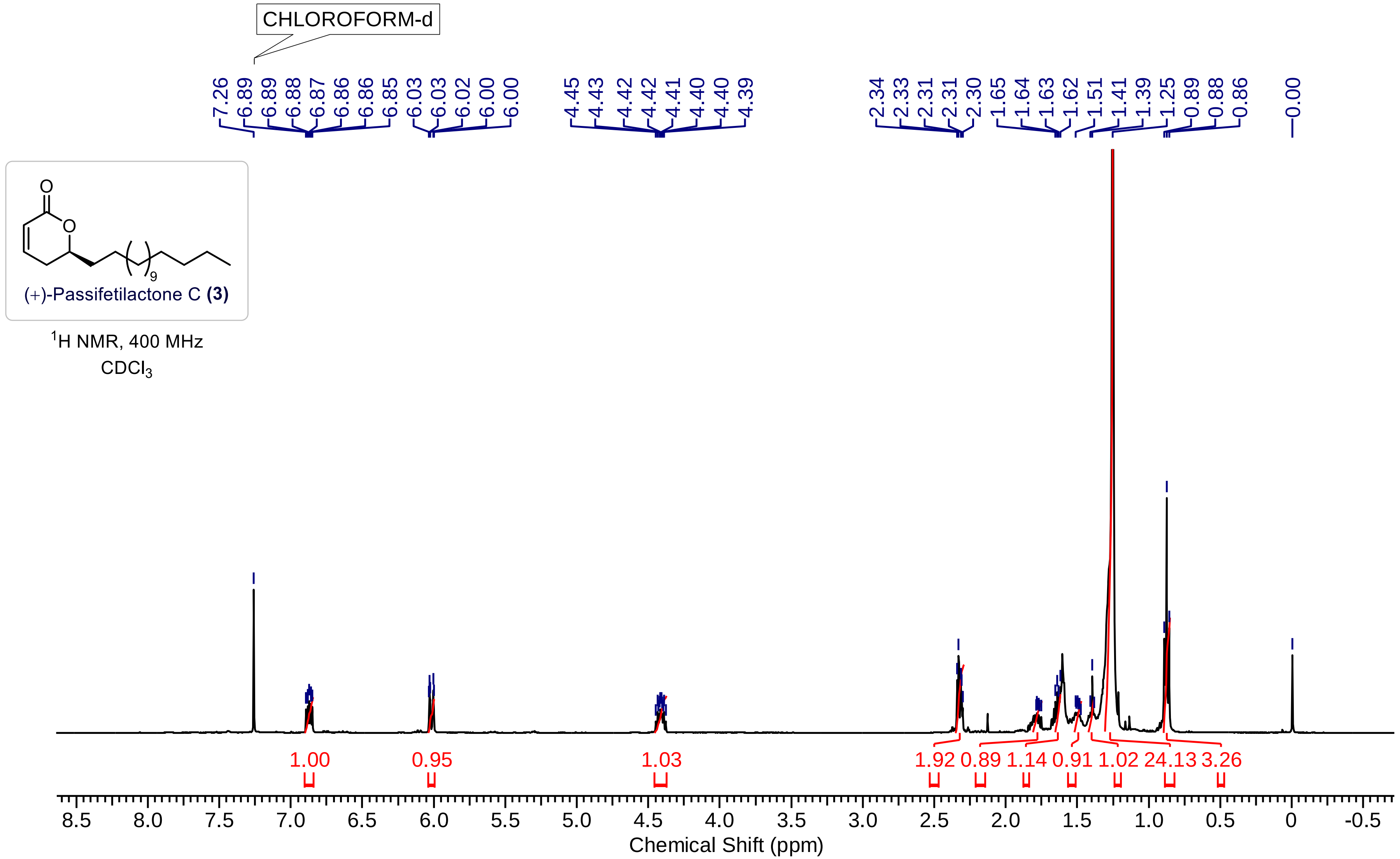

$^{13}C\{^{1}H\}$ NMR spectrum of Passifetilactone C (3):

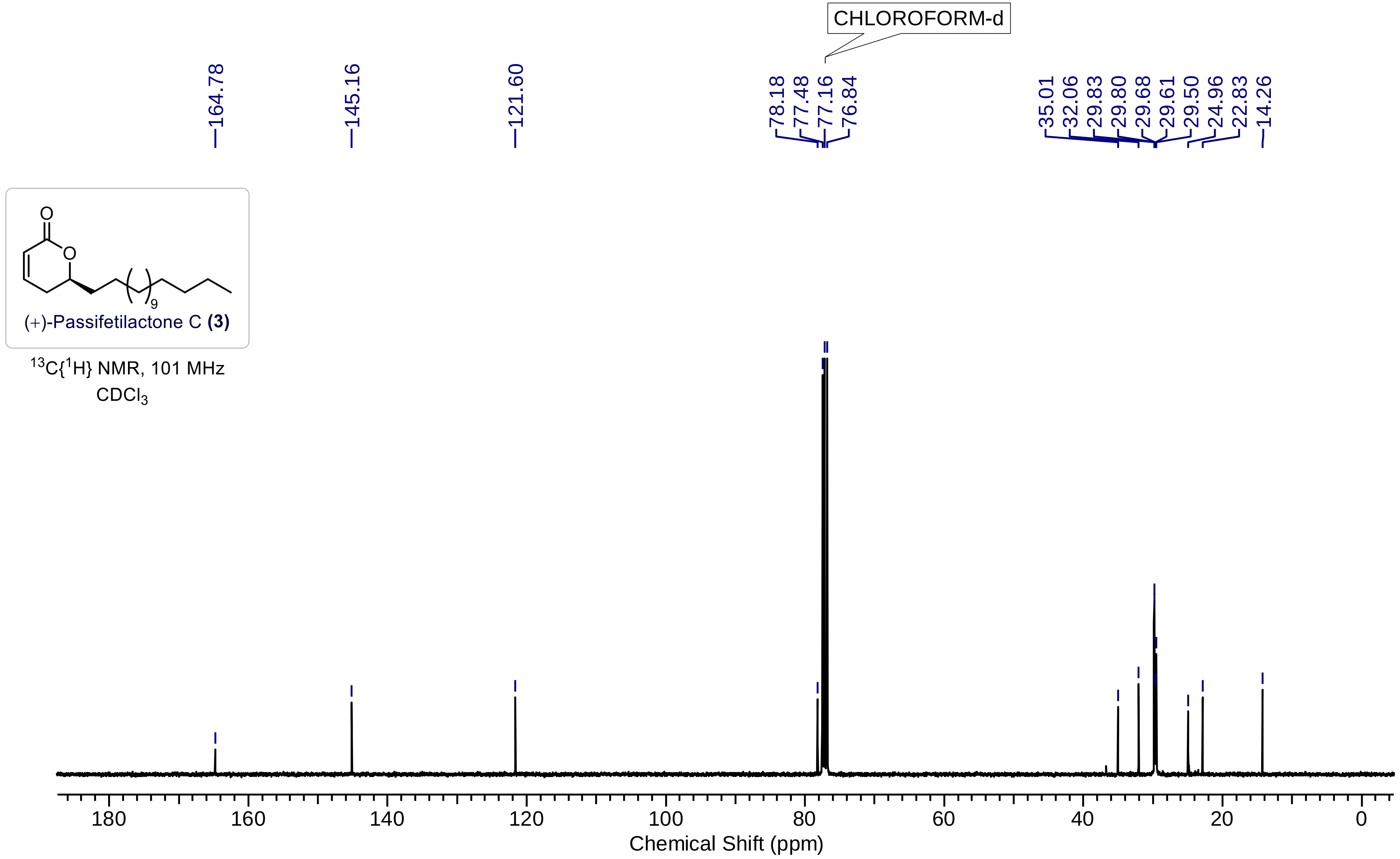

^{1}H NMR spectrum of 8-((Tert-butyldimethylsilyl)oxy)octanal(23):

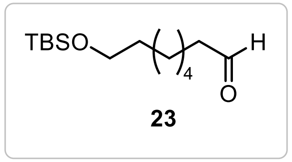

^{1}H NMR, 400 MHz
$CDCl_3$

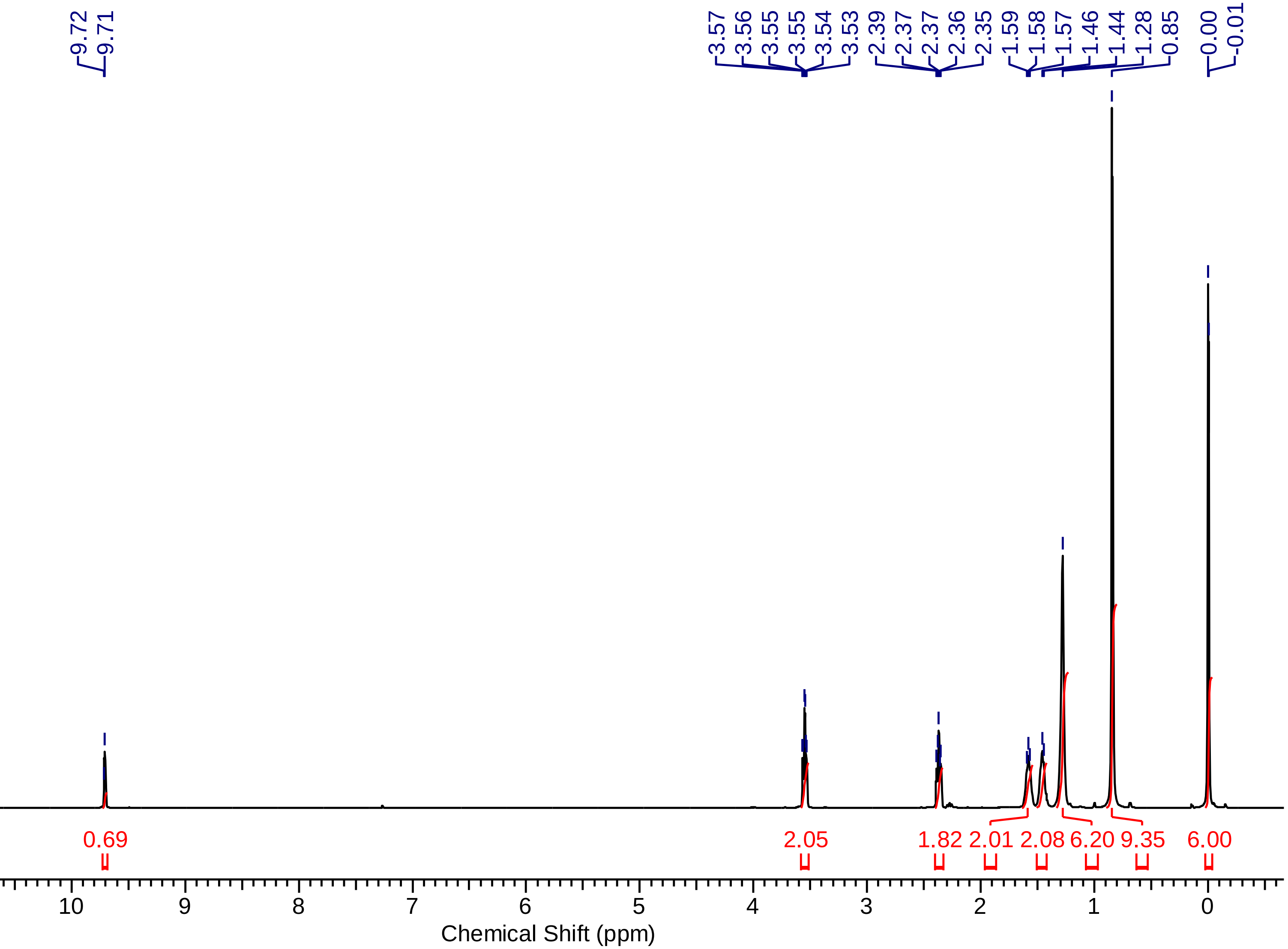

$^{13}C\{^{1}H\}$ NMR spectrum of 8-((Tert-butyldimethylsilyl)oxy)octanal (23):

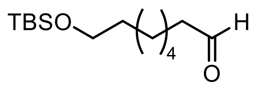

23

$^{13}C\{^{1}H\}$ NMR, 101 MHz

$CDCl_3$

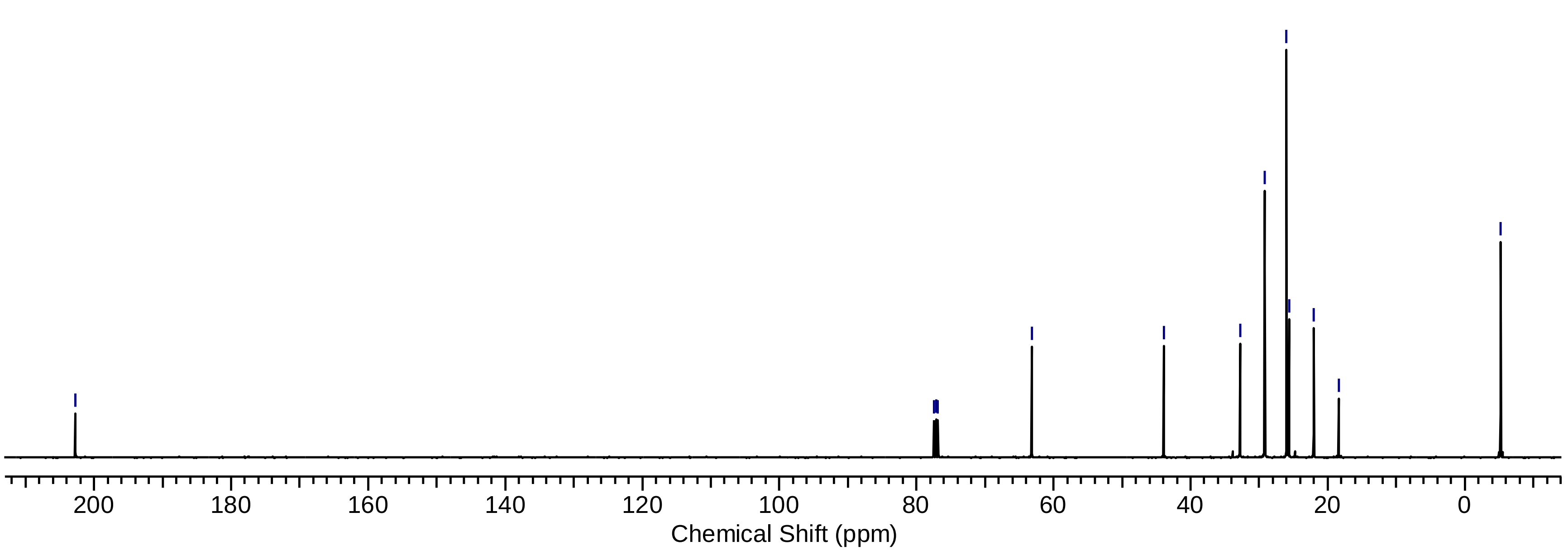

^{1}H NMR spectrum of (*Z*)-Tert-butyldimethyl(pentadec-8-en-1-yloxy)silane (25):

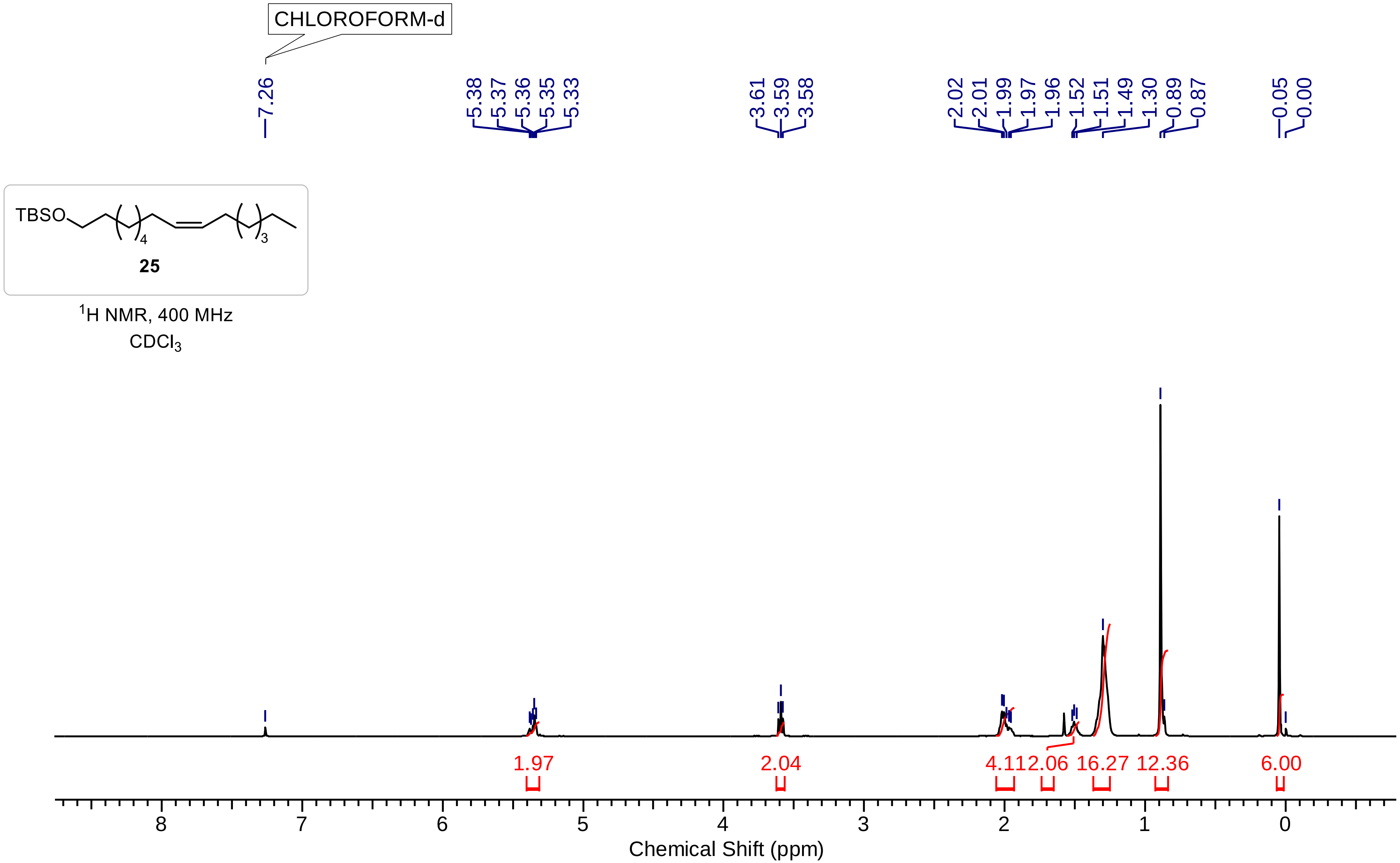

$^{13}C\{^{1}H\}$ NMR spectrum of (*Z*)-Tert-butyldimethyl(pentadec-8-en-1-yloxy)silane (25):

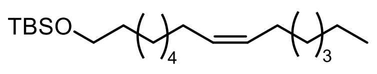

25

$^{13}C\{^{1}H\}$ NMR, 101 MHz
$CDCl_3$

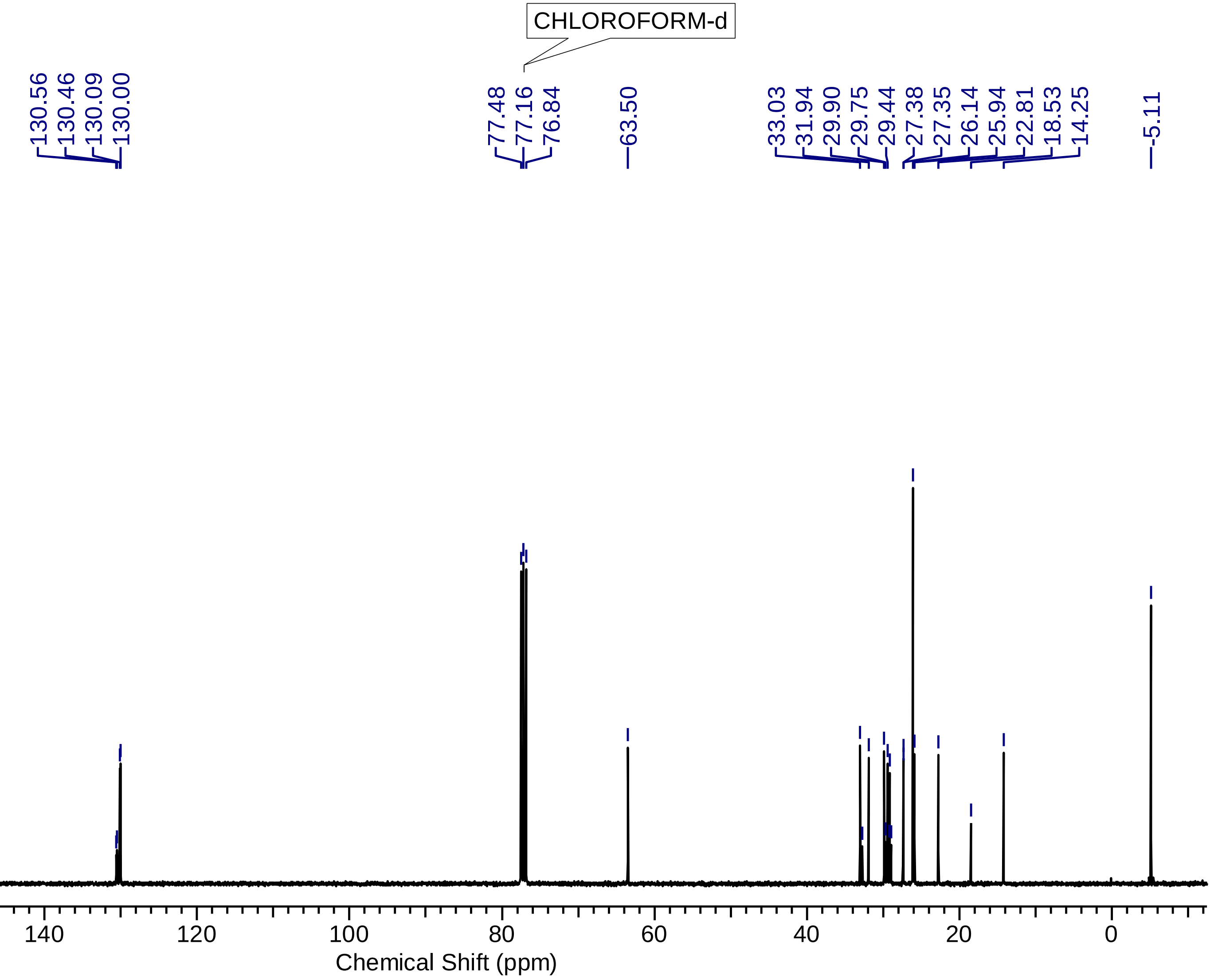

^{1}H NMR spectrum of (*Z*)-Pentadec-8-en-1-ol (S2):

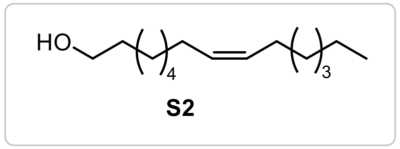

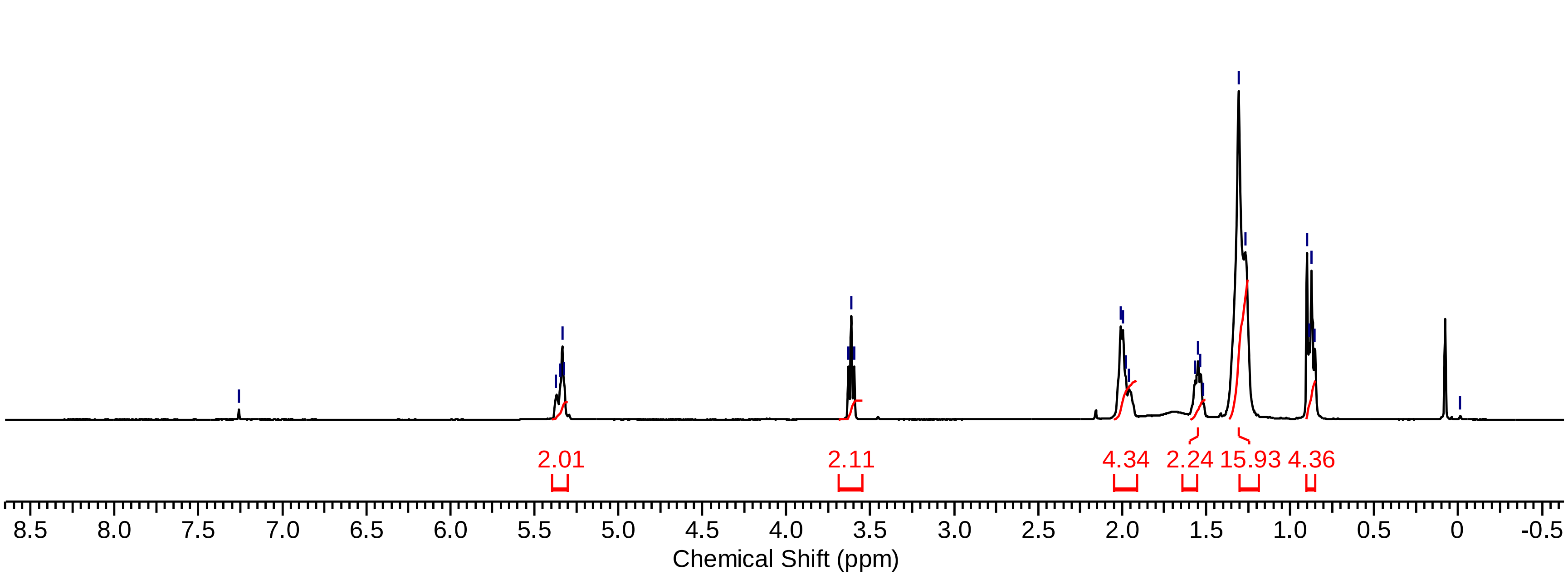

$^{13}C\{^1H\}$ NMR spectrum of (*Z*)-Pentadec-8-en-1-ol (S2):

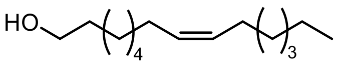

S2

$^{13}C\{^1H\}$ NMR, 101 MHz
$CDCl_3$

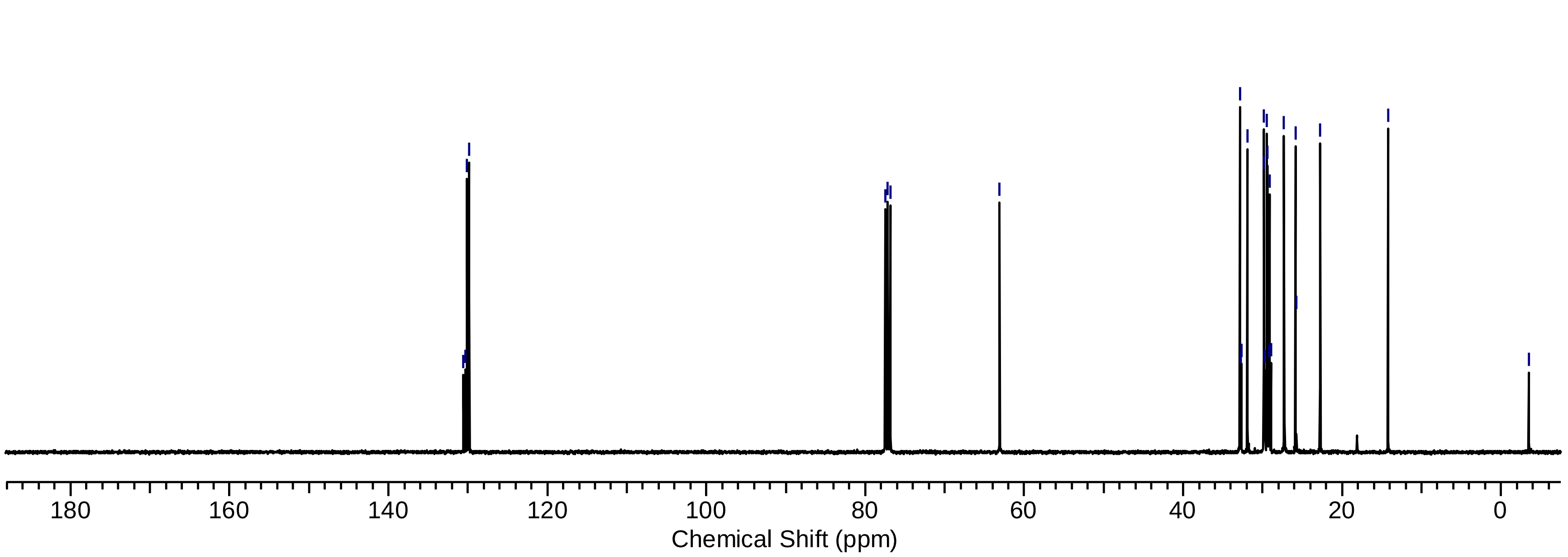

^{1}H NMR spectrum of (*Z*)-15-Bromopentadec-7-ene (26):

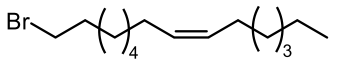

26

^{1}H NMR, 400 MHz
$CDCl_3$

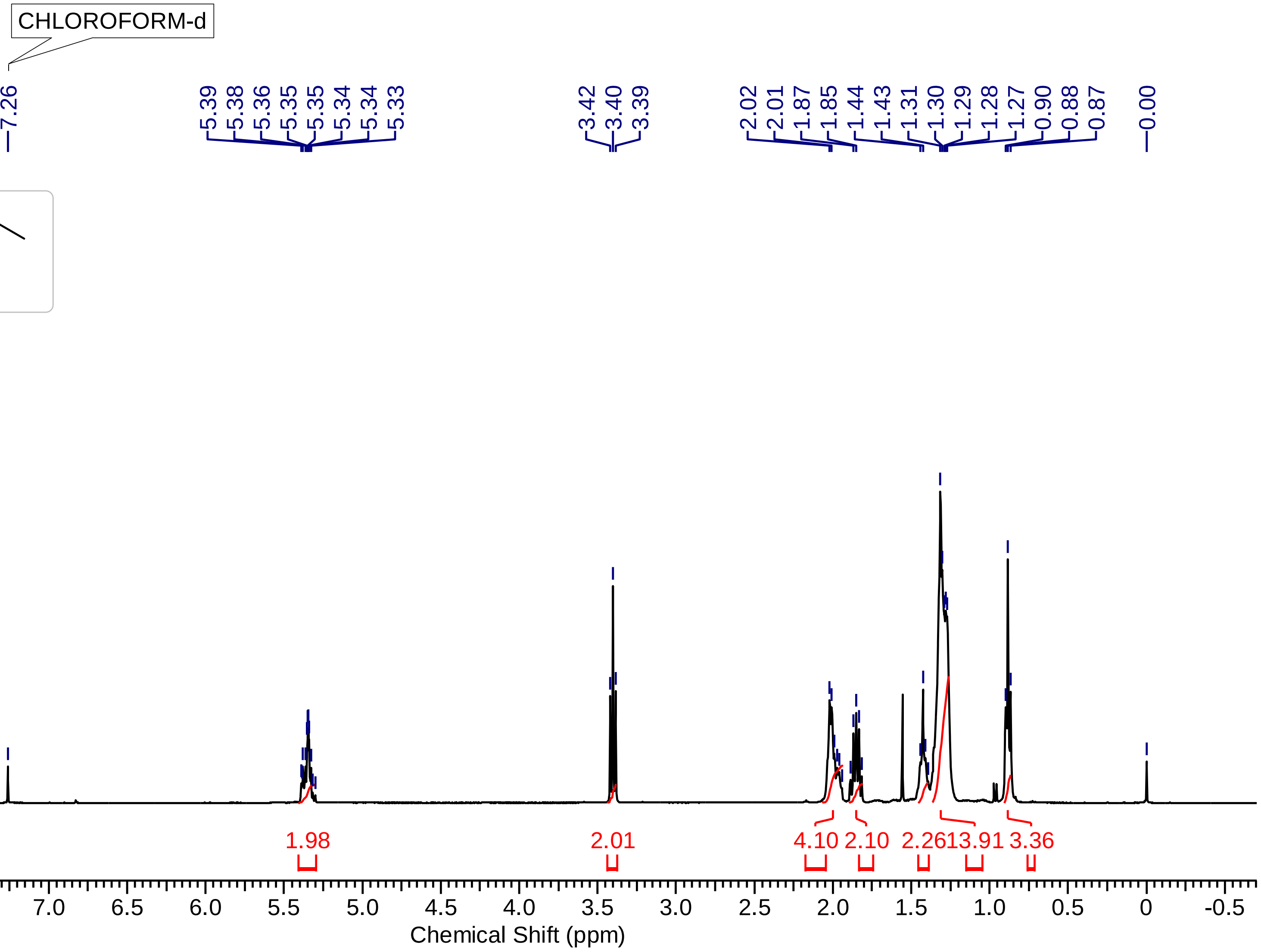

$^{13}C\{^1H\}$ NMR spectrum of (*Z*)-15-Bromopentadec-7-ene (26):

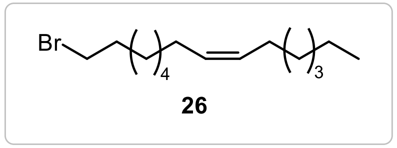

$^{13}C\{^1H\}$ NMR, 101 MHz
$CDCl_3$

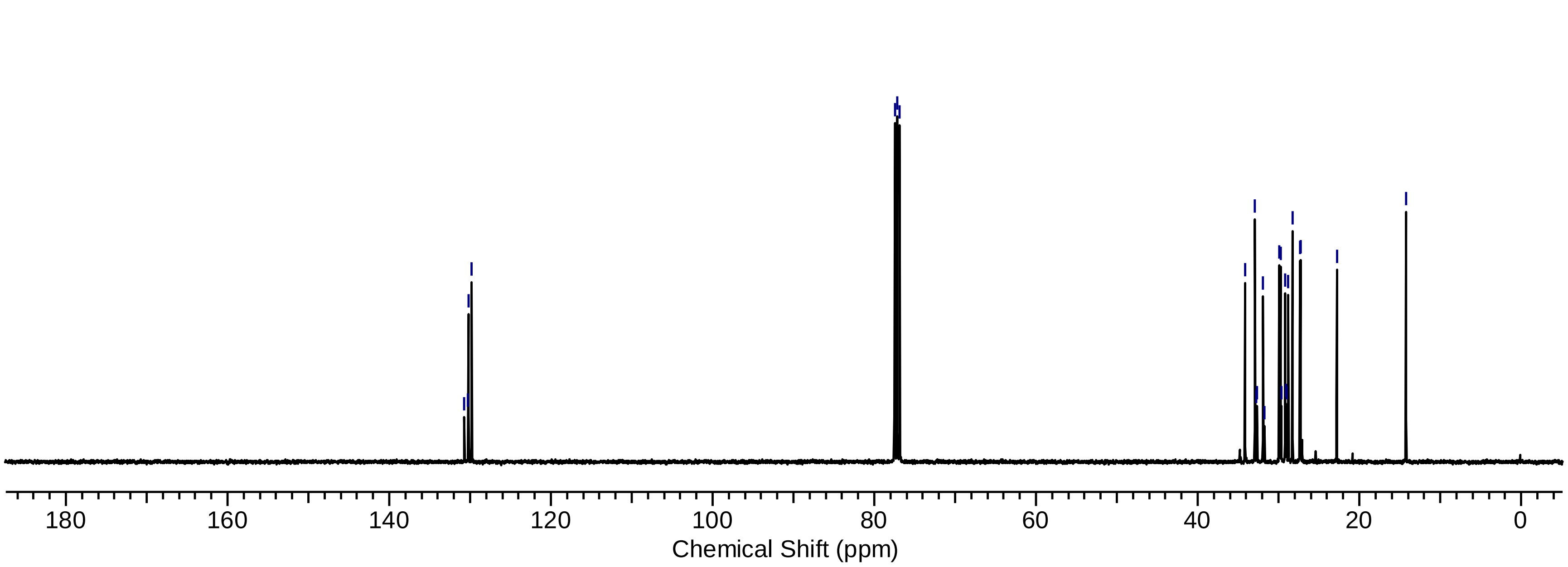

^{1}H NMR spectrum of 9-((Tert-butyldimethylsilyl)oxy)nonan-1-ol (S3):

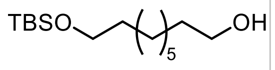

S3

^{1}H NMR, 400 MHz
$CDCl_3$

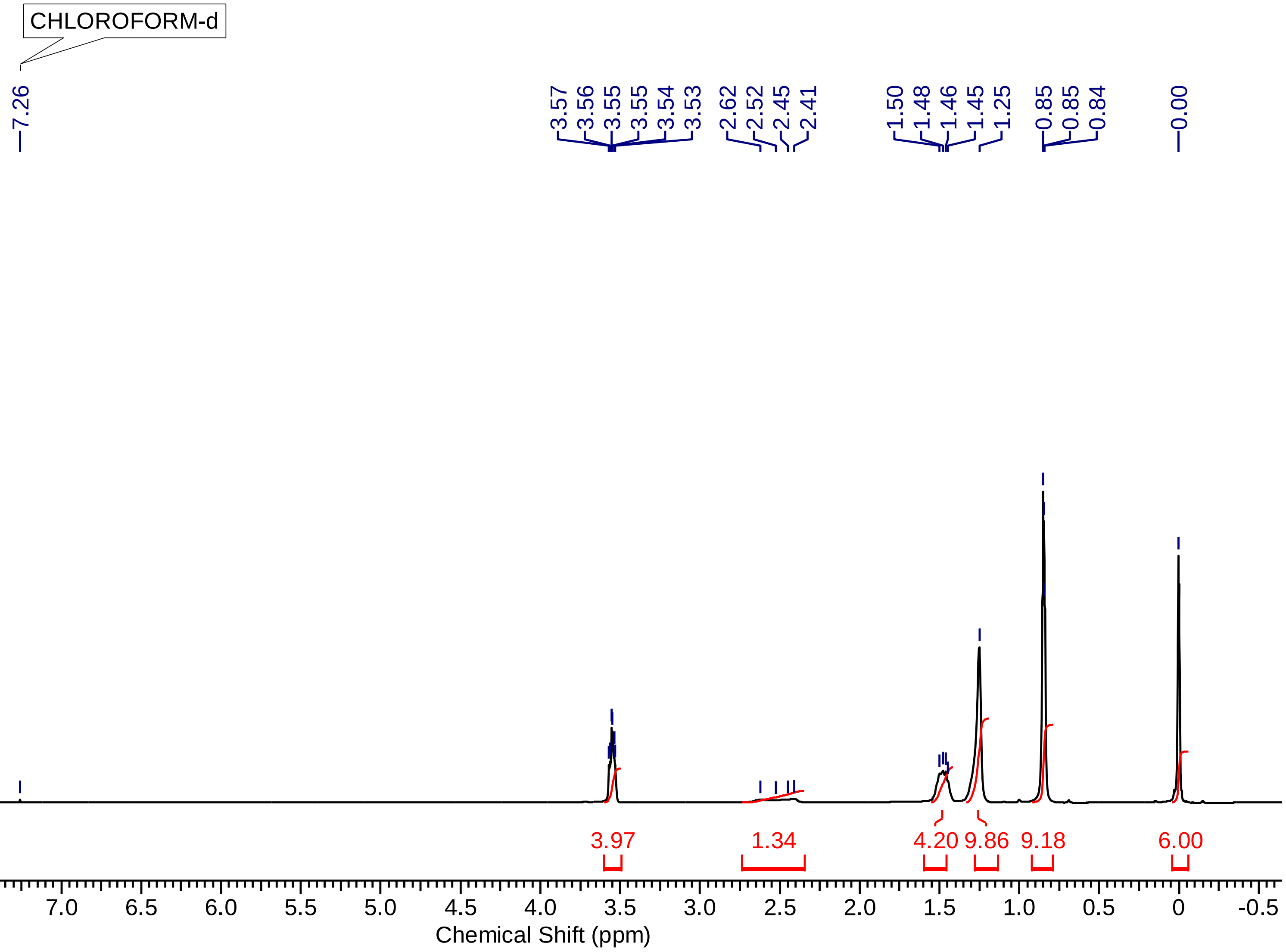

$^{13}C\{^1H\}$ NMR spectrum of 9-((Tert-butyldimethylsilyl)oxy)nonan-1-ol (S3):

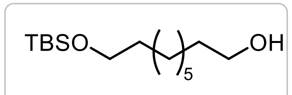

S3

$^{13}C\{^1H\}$ NMR, 101 MHz
$CDCl_3$

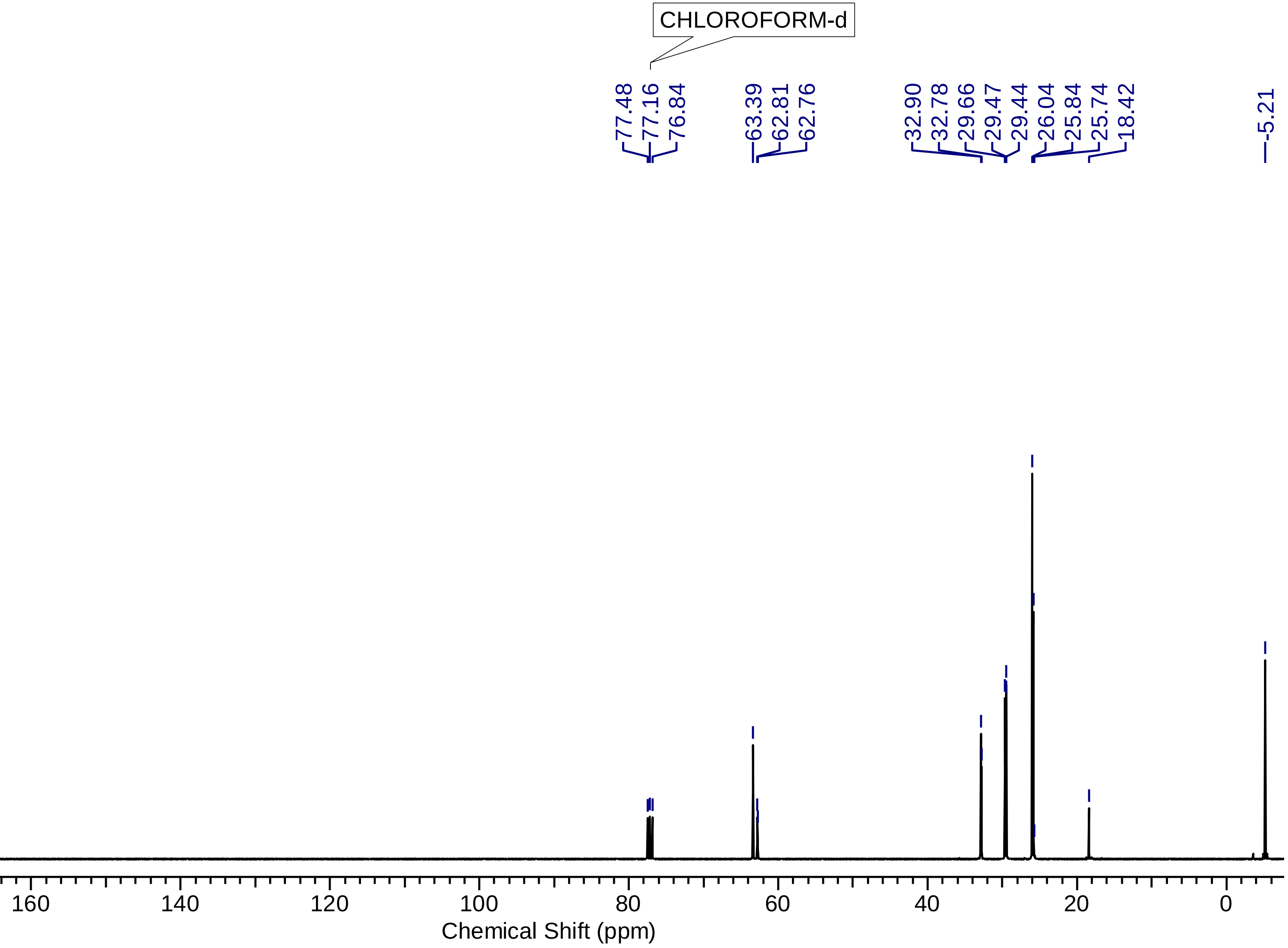

^{1}H NMR spectrum of 9-((Tert-butyldimethylsilyl)oxy)nonanal (29):

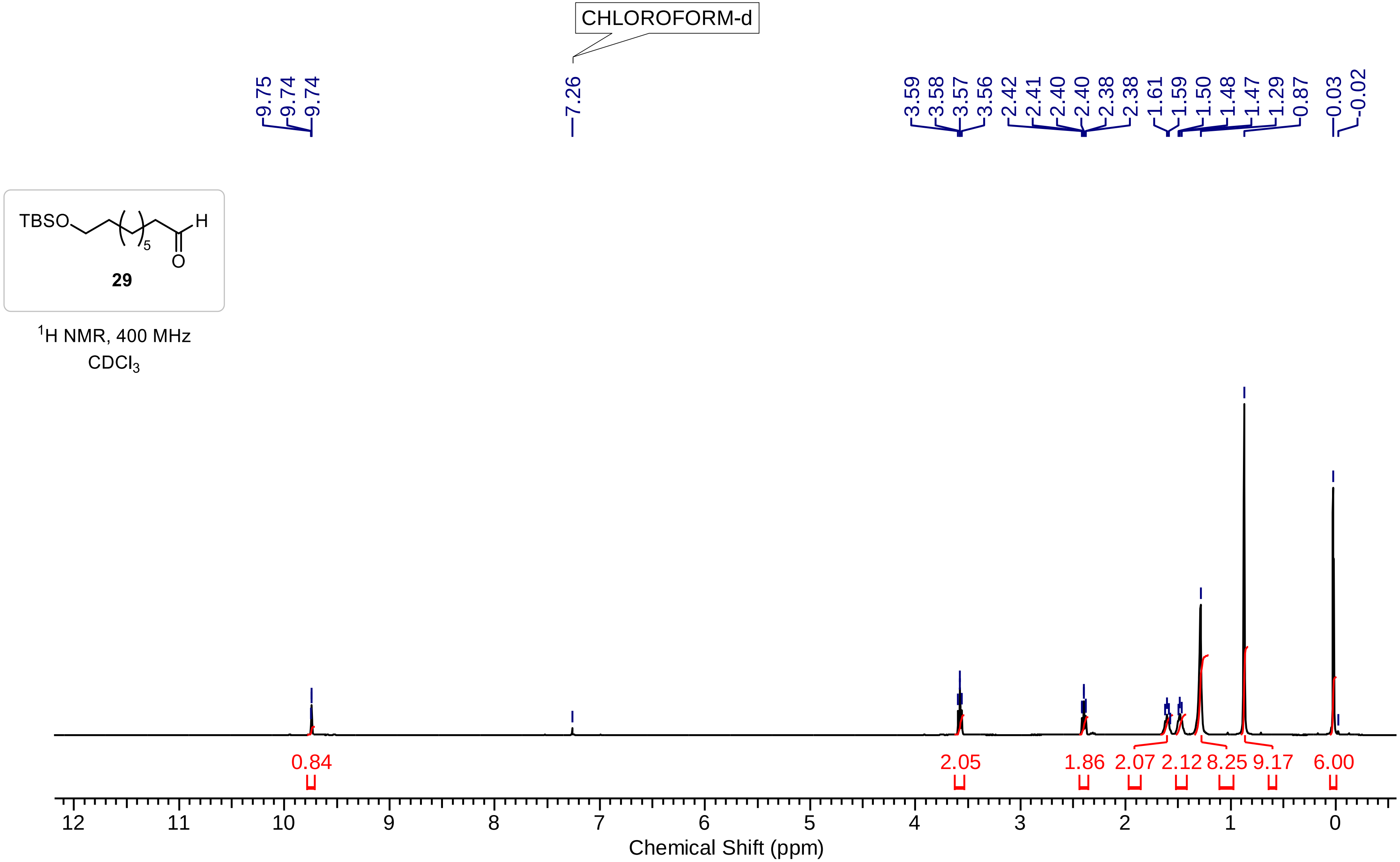

$^{13}C\{^{1}H\}$ NMR spectrum of 9-((Tert-butyldimethylsilyl)oxy)nonanal (29):

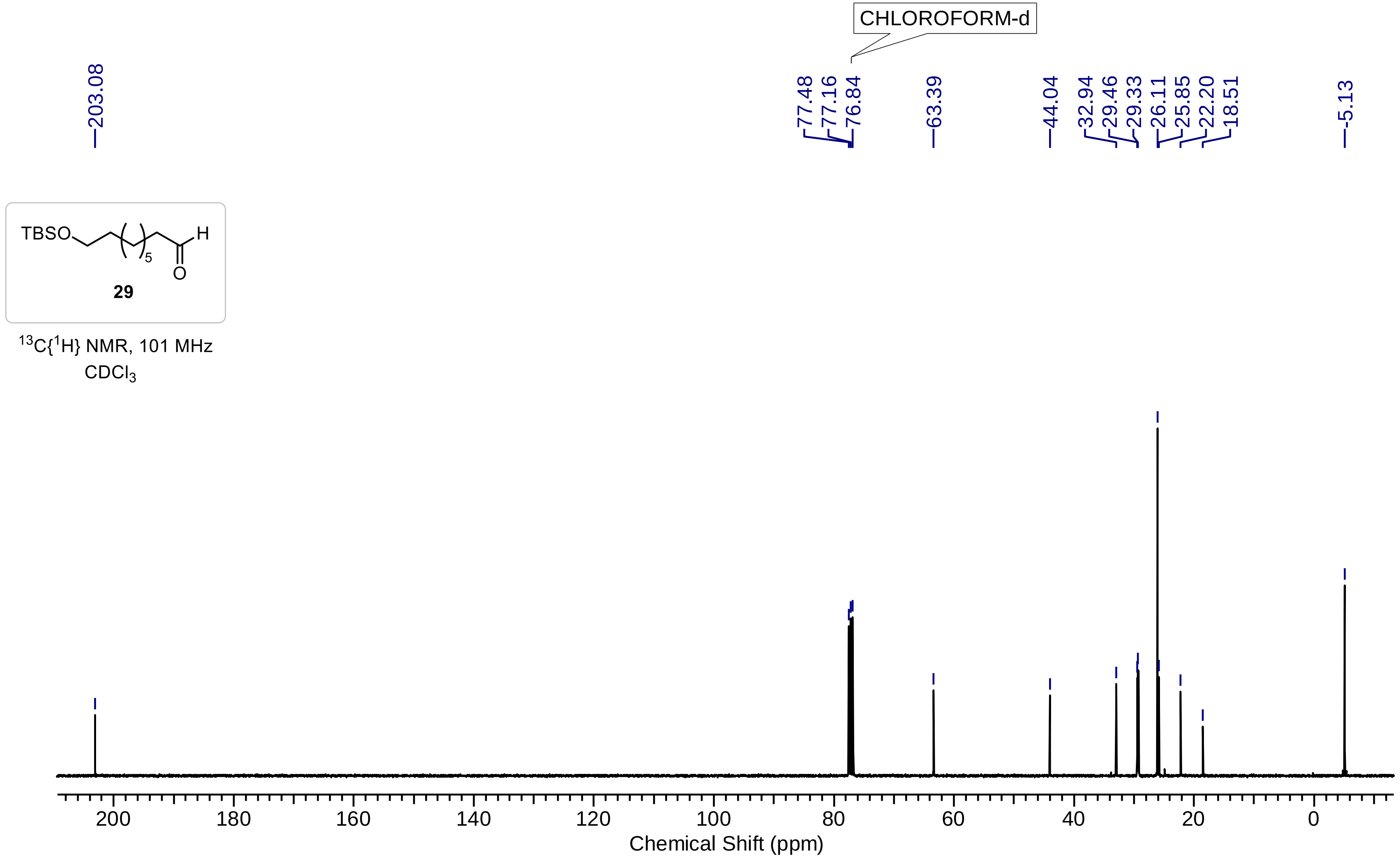

^{1}H NMR spectrum of (*Z*)-Tert-butyl(hexadec-9-en-1-yloxy)dimethylsilane (30):

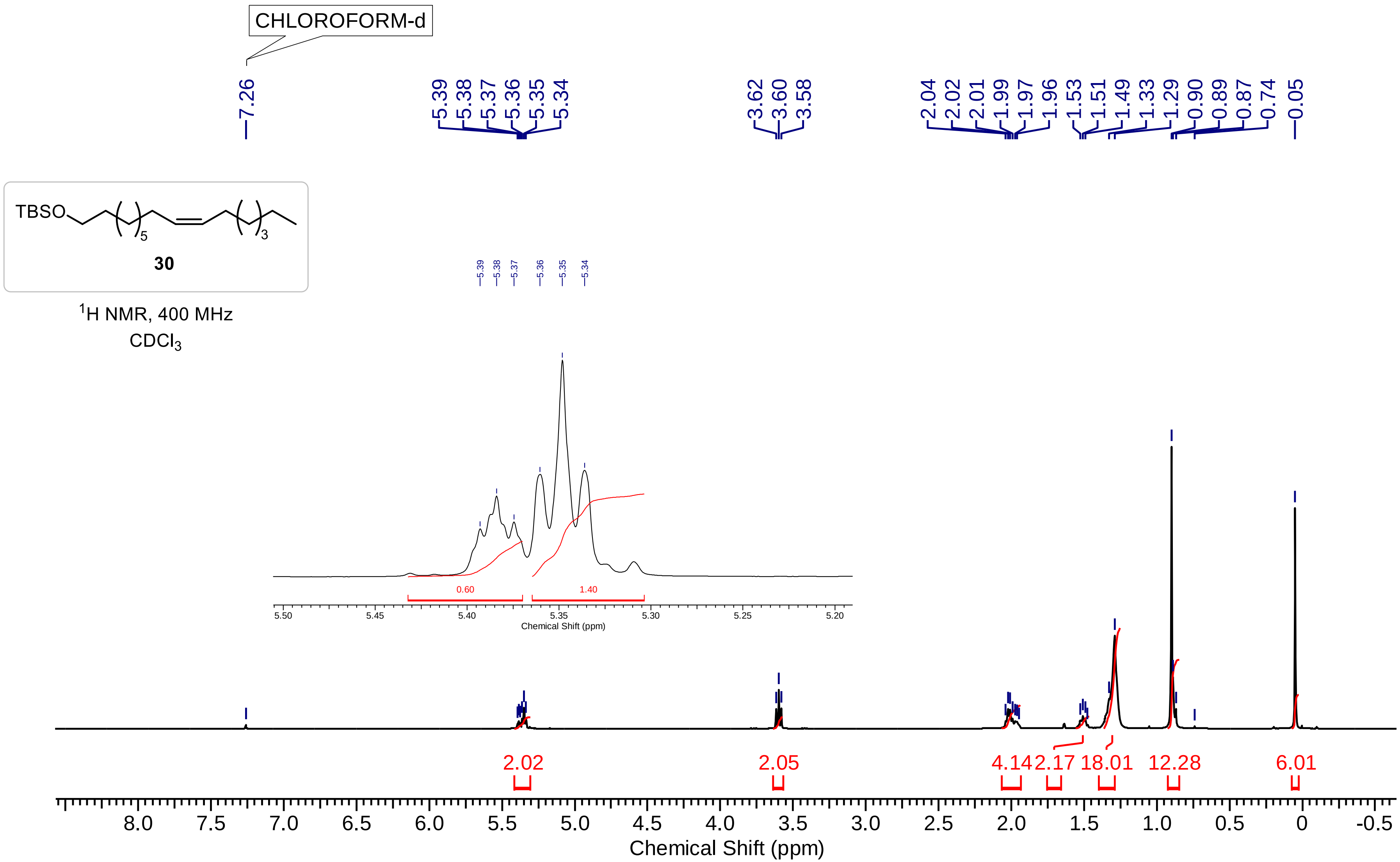

^{13}C{^{1}H} NMR spectrum of (*Z*)-Tert-butyl(hexadec-9-en-1-yloxy)dimethylsilane (30):

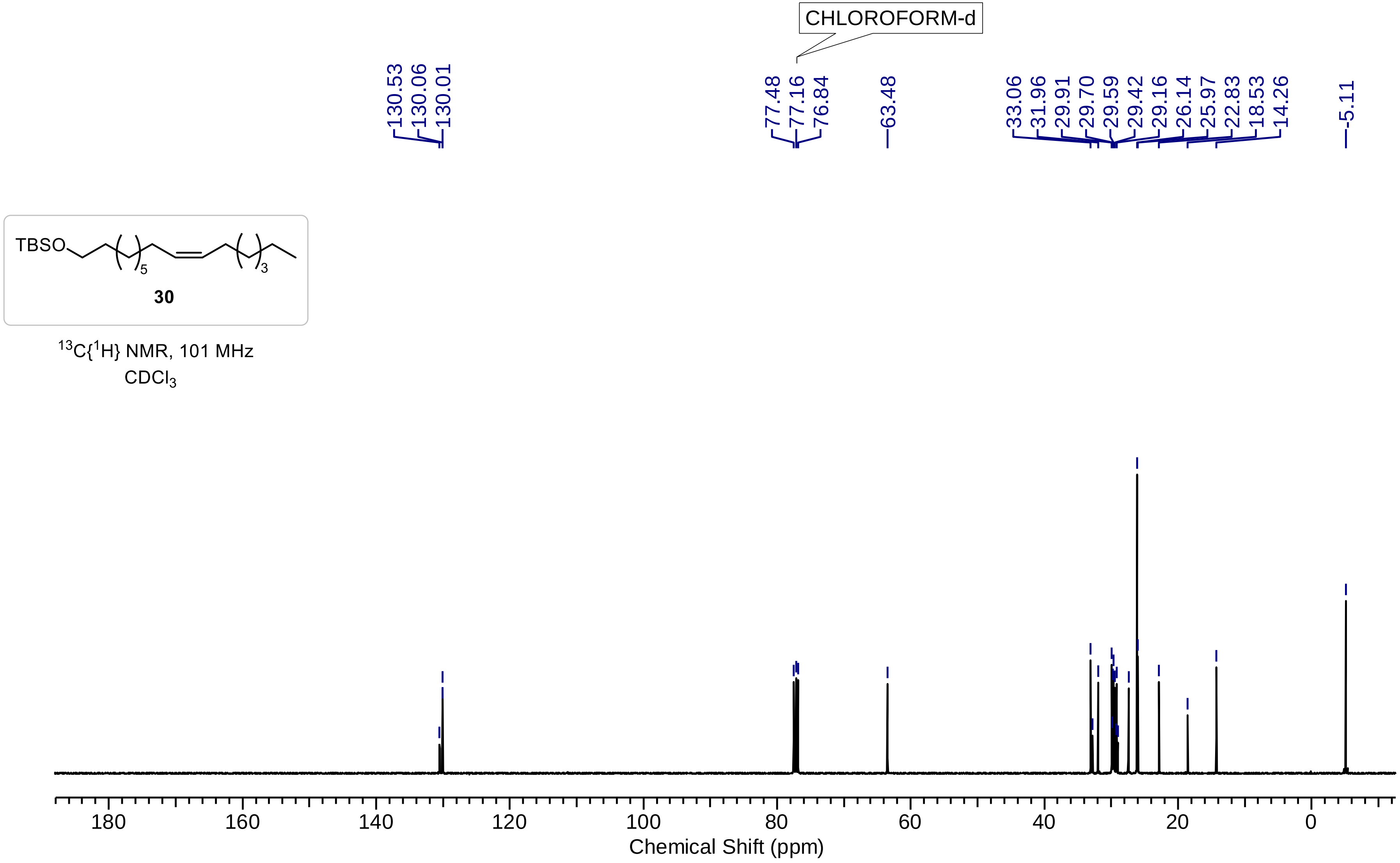

^{1}H NMR spectrum of (*Z*)-Hexadec-9-en-1-ol (S4):

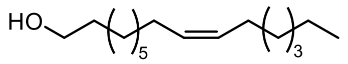

S4

^{1}H NMR, 400 MHz
$CDCl_3$

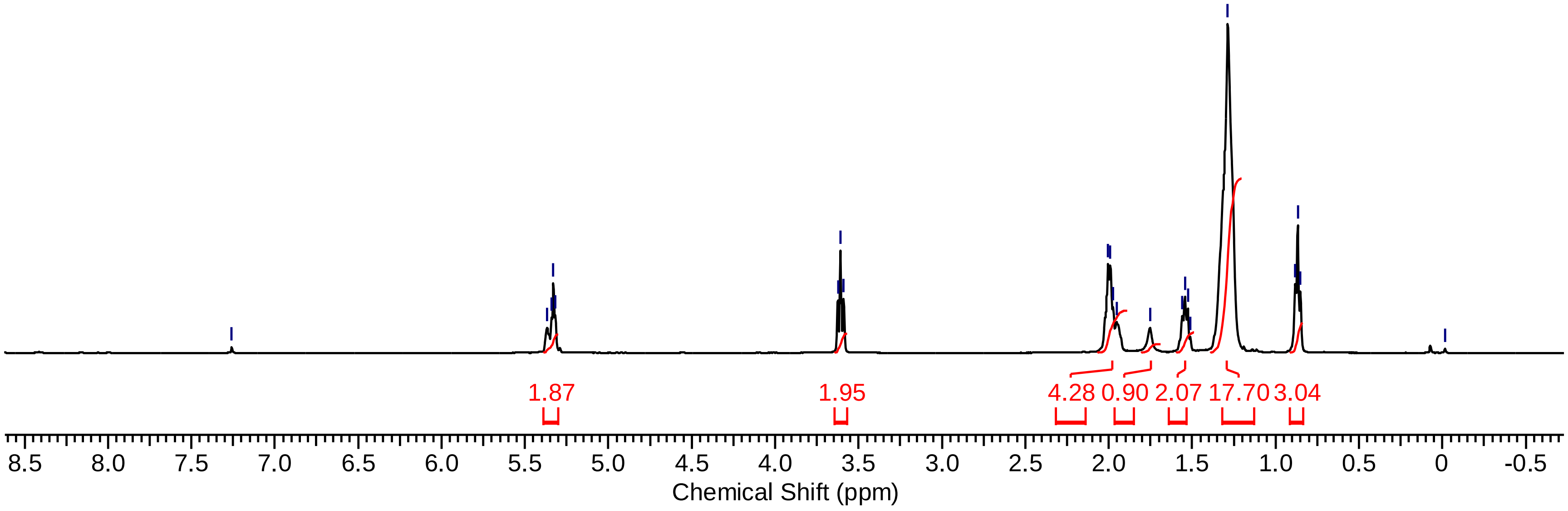

$^{13}C\{^1H\}$ NMR spectrum of (*Z*)-Hexadec-9-en-1-ol (S4):

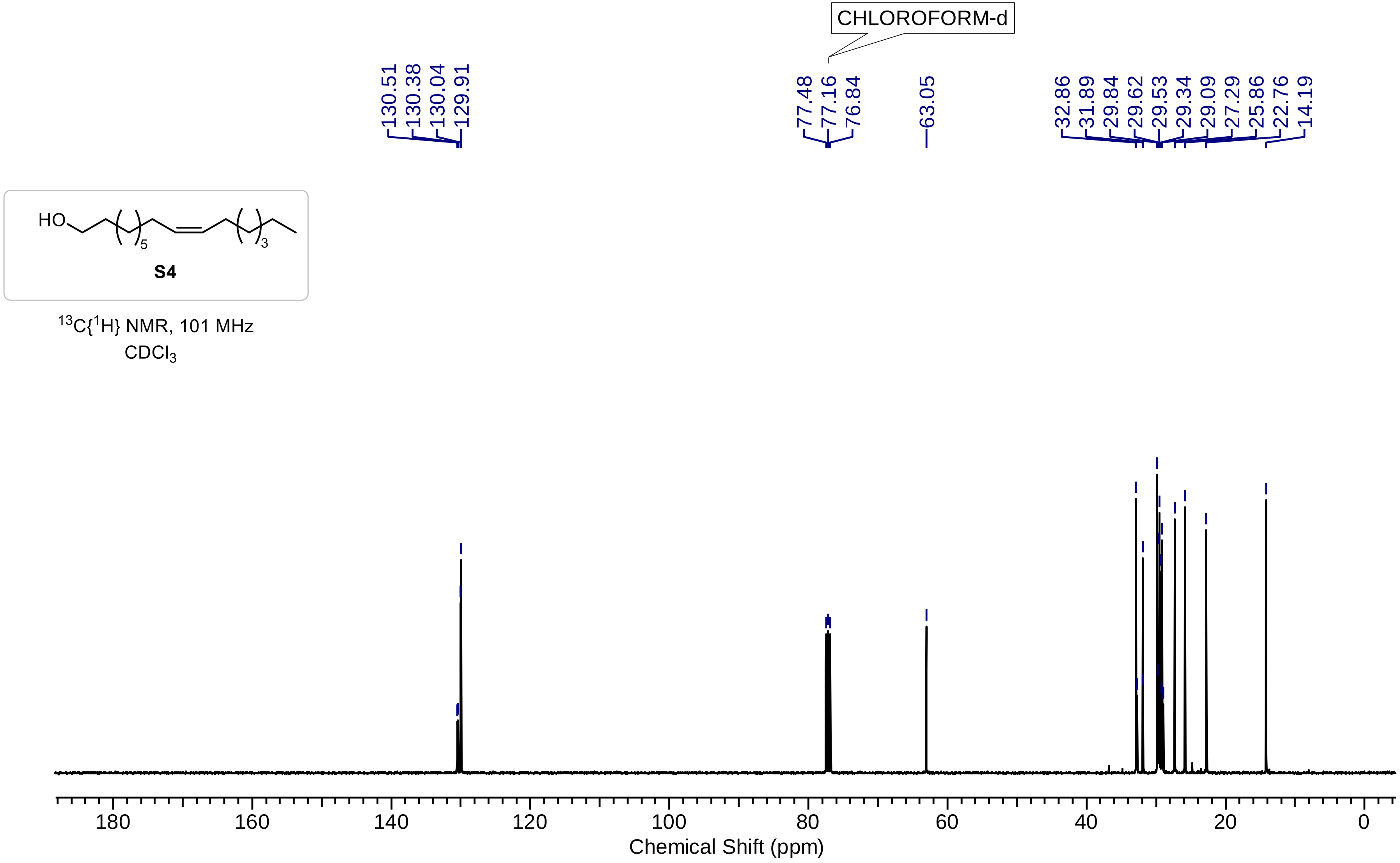

^{1}H NMR spectrum of (*Z*)-Hexadec-9-enal (31):

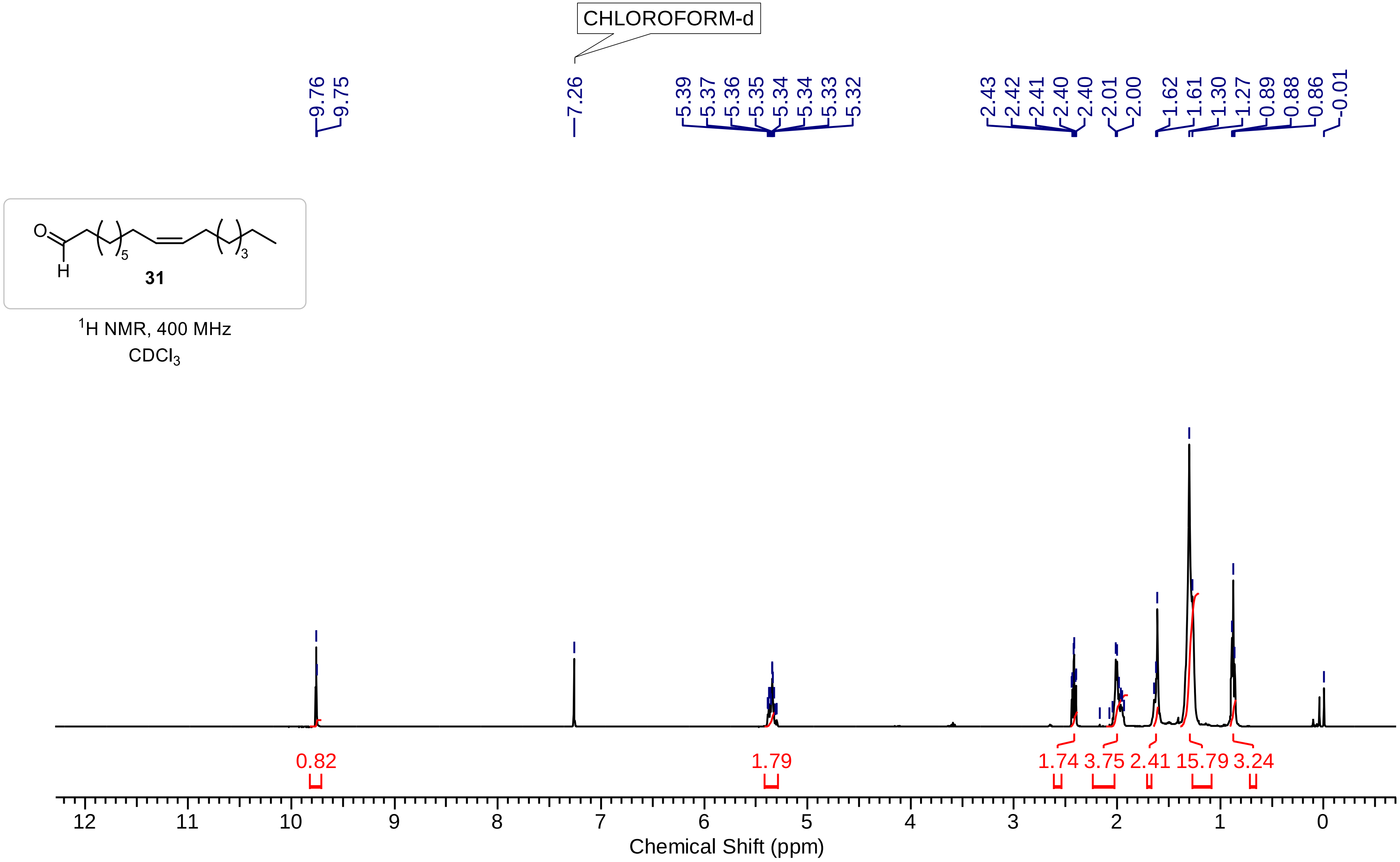

$^{13}C\{^{1}H\}$ NMR spectrum of (*Z*)-Hexadec-9-enal (31):

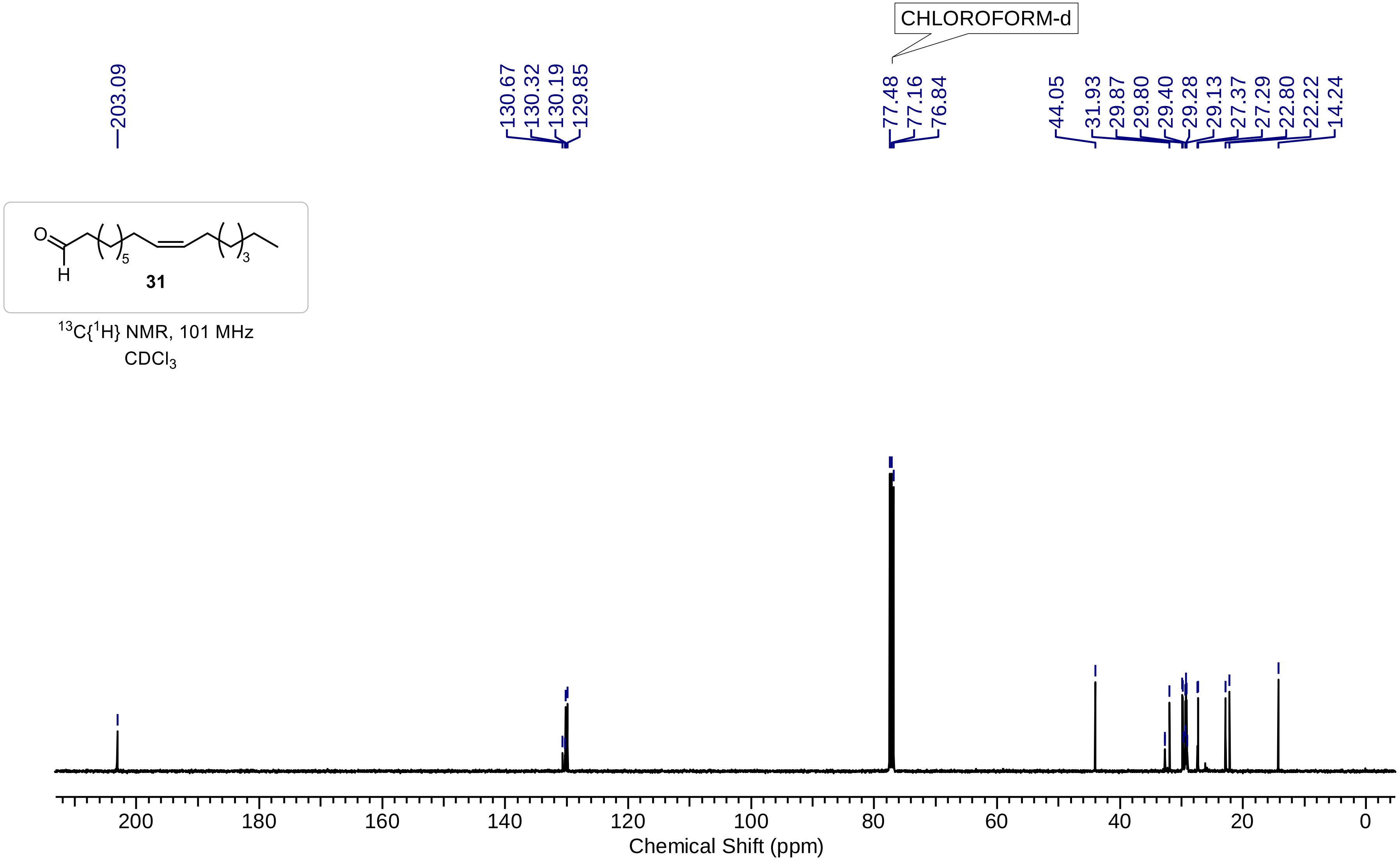

^{1}H NMR spectrum of (*Z*)-1-(Furan-2-yl)hexadec-9-en-1-ol (27):

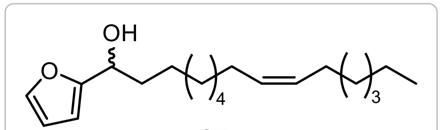

27

^{1}H NMR, 400 MHz
$CDCl_3$

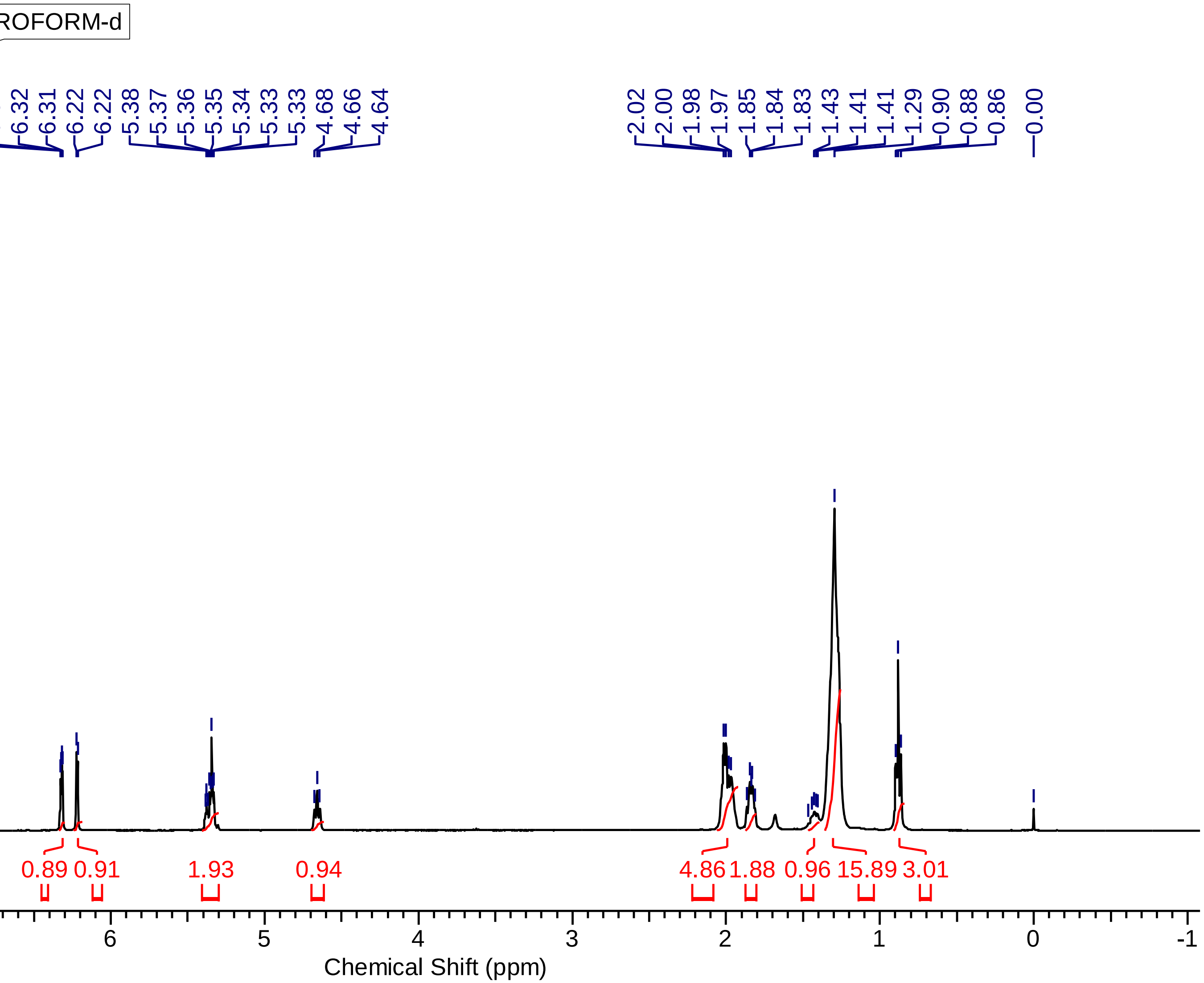

^{13}C{^{1}H} NMR spectrum of (*Z*)-1-(Furan-2-yl)hexadec-9-en-1-ol (27):

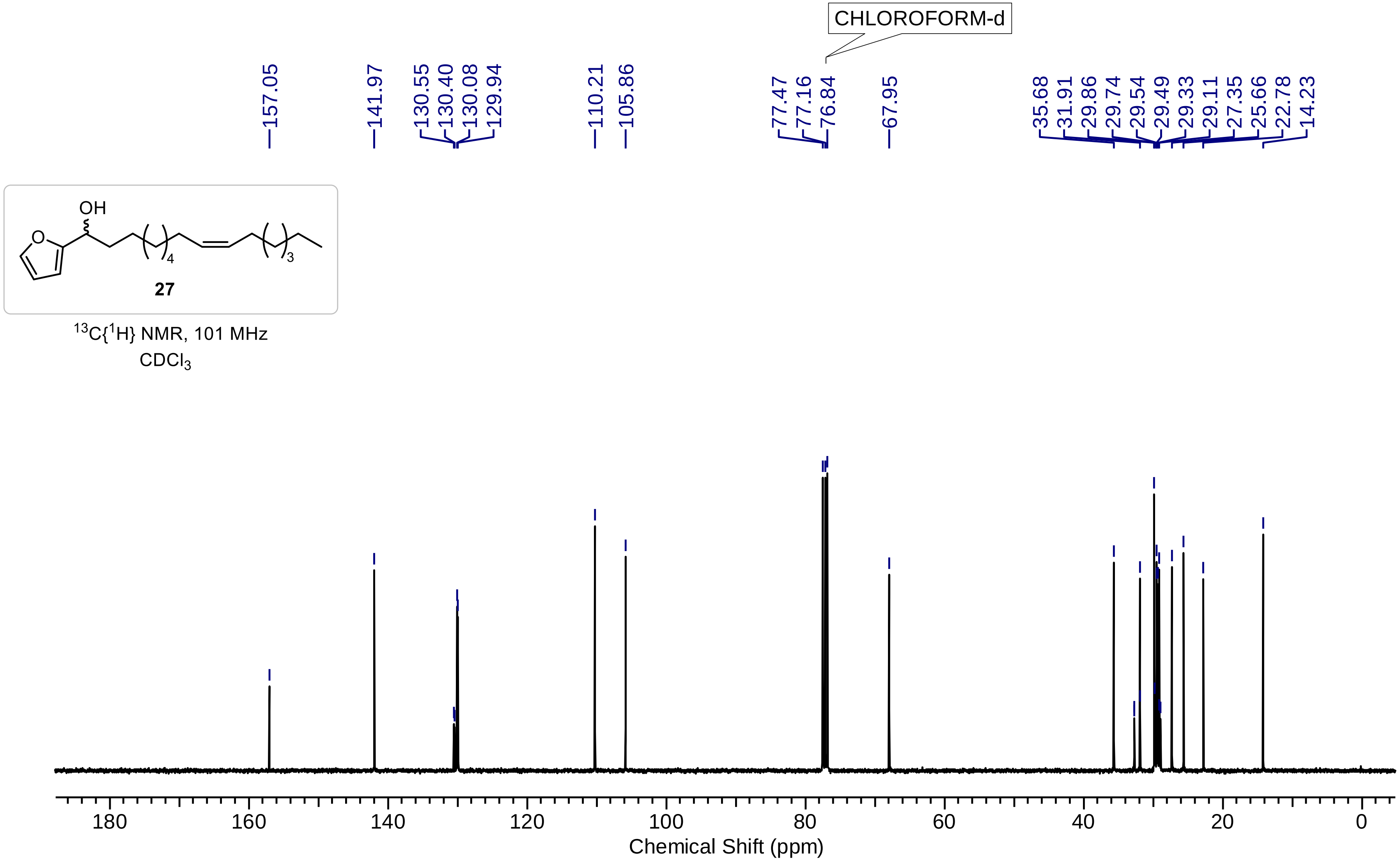

^{1}H NMR spectrum of (*Z*)-1-(Furan-2-yl)hexadec-9-en-1-one (17b):

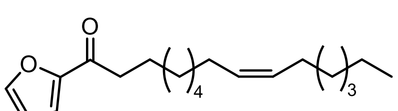

17b

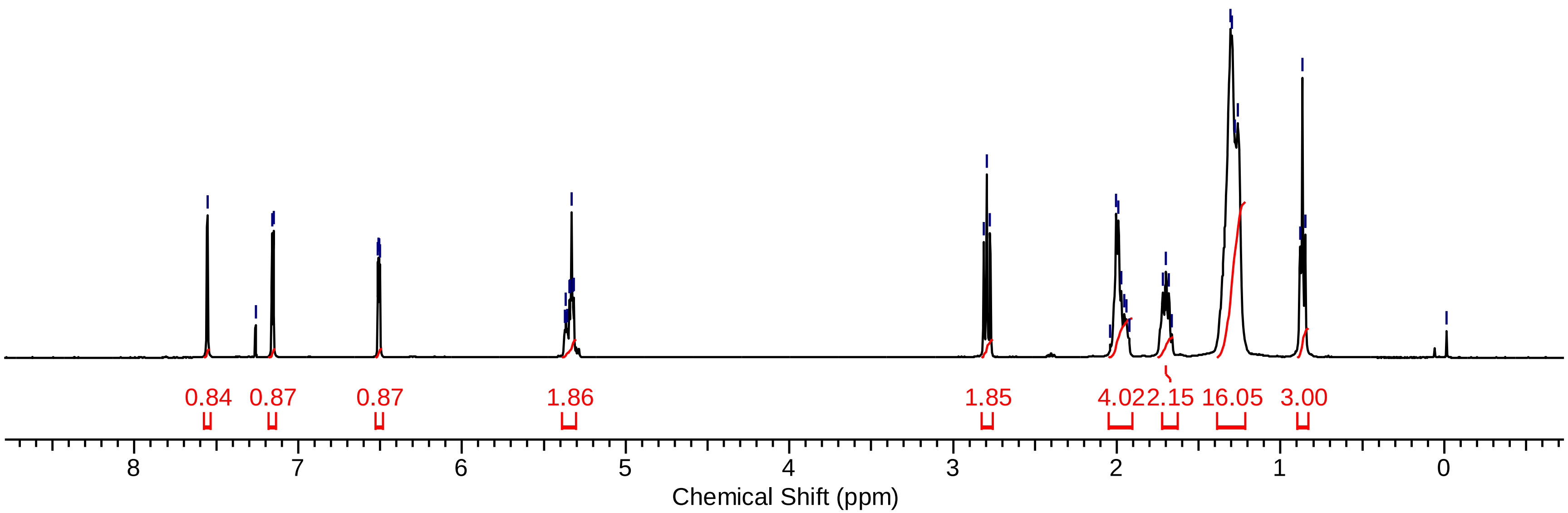

$^{13}C\{^1H\}$ NMR spectrum of (*Z*)-1-(Furan-2-yl)hexadec-9-en-1-one (17b):

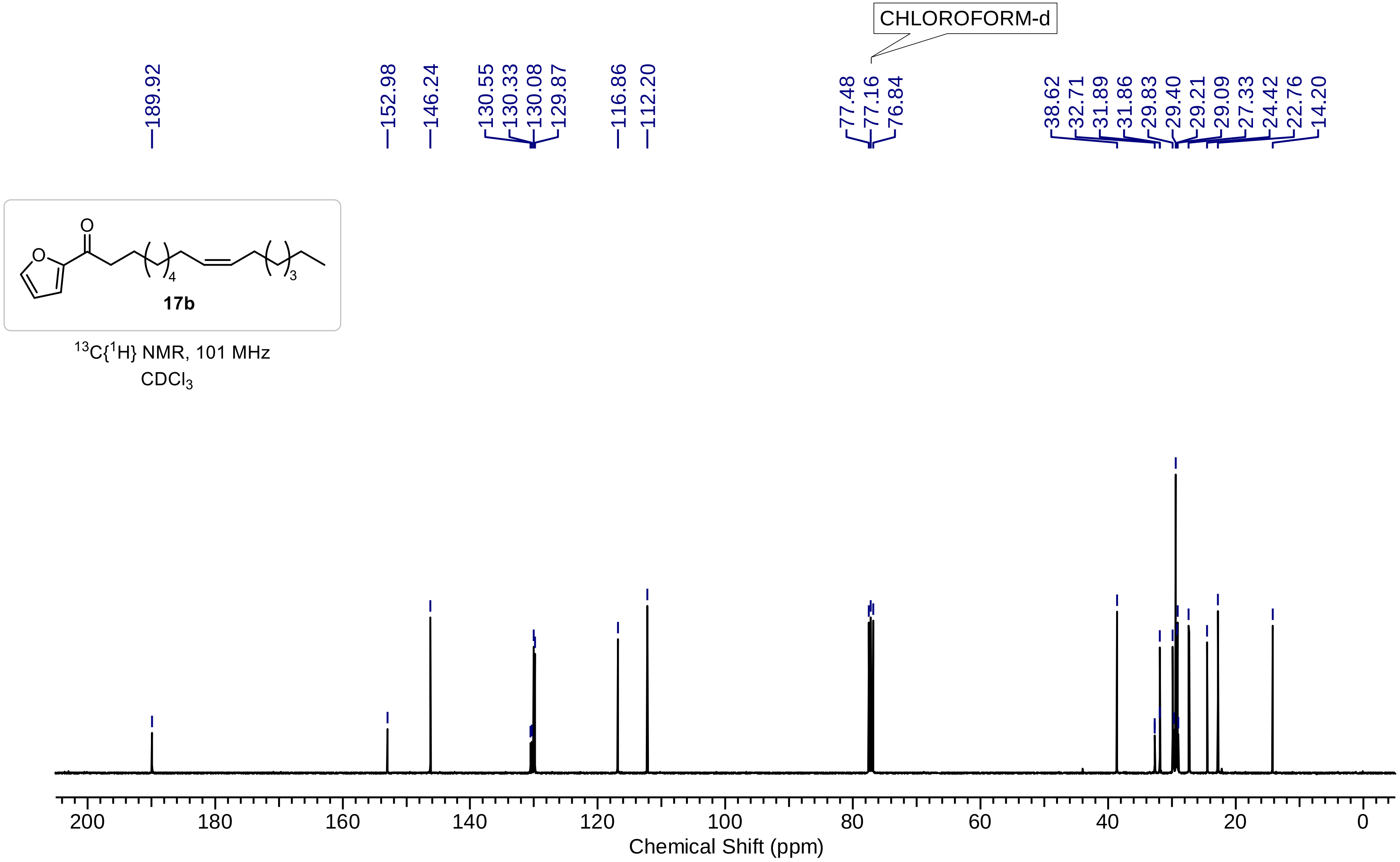

^{1}H NMR spectrum of (*S*,*Z*)-1-(Furan-2-yl)hexadec-9-en-1-ol (18b):

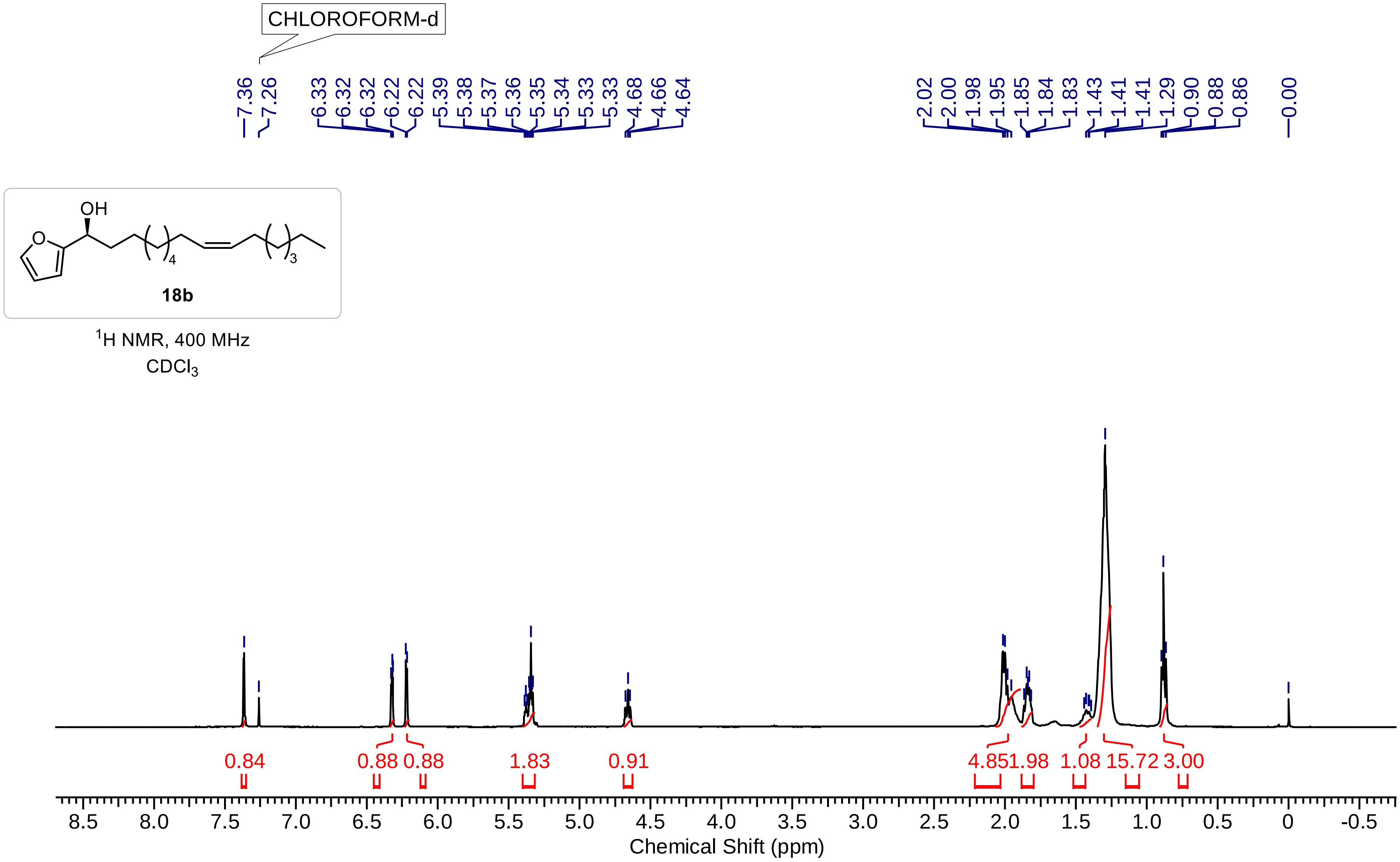

$^{13}C\{^1H\}$ NMR spectrum of (*S*,*Z*)-1-(Furan-2-yl)hexadec-9-en-1-ol (18b):

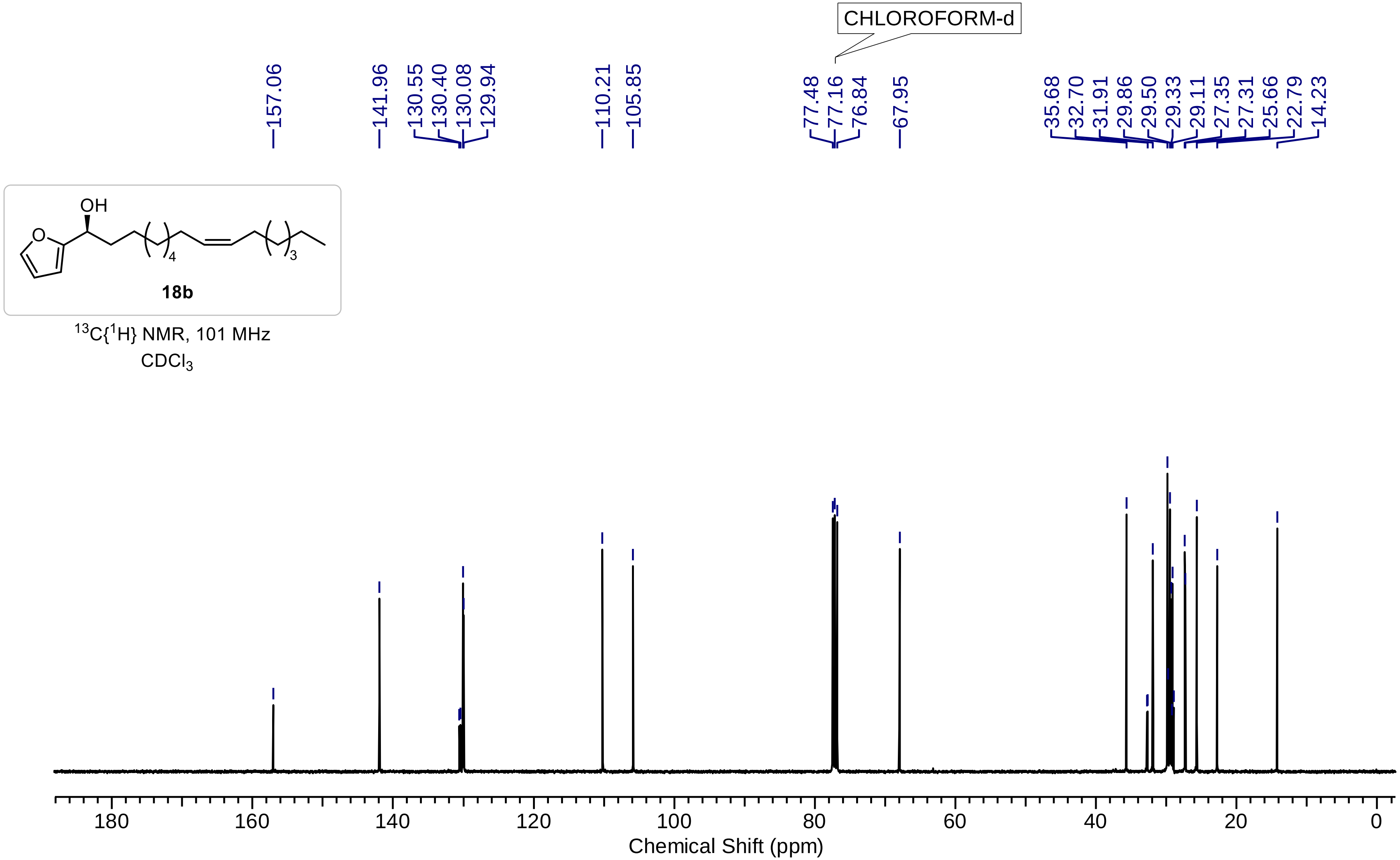

HPLC spectrum of (±)-(*Z*)-1-(Furan-2-yl)hexadec-9-en-1-ol (27):

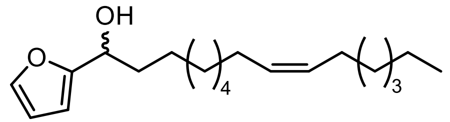

27

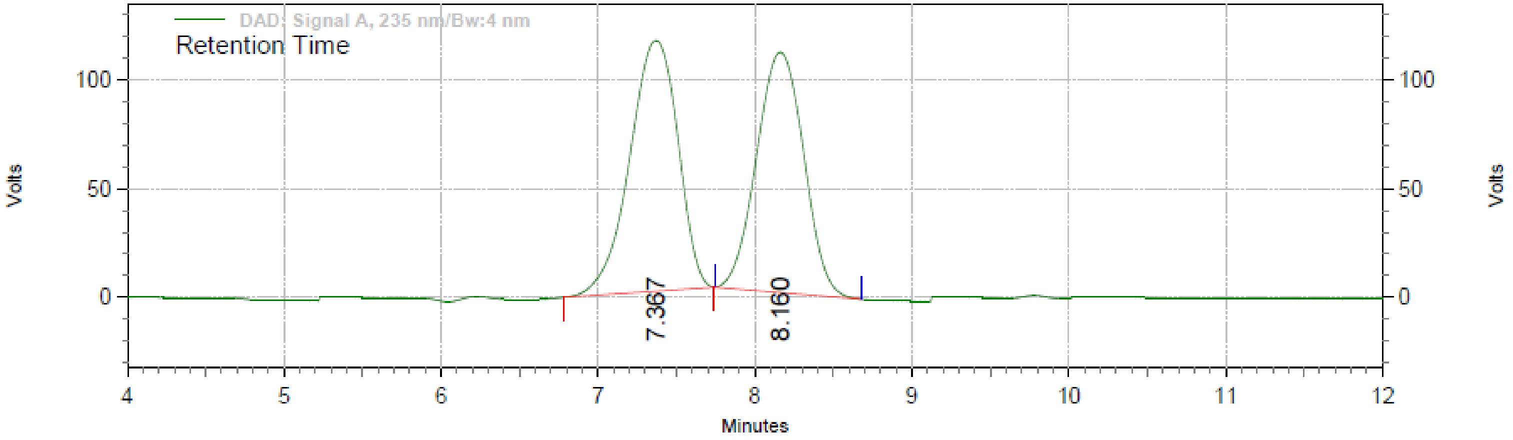

DAD: Signal A,
235 nm/Bw:4 nm
Results

Retention Time	Area	Area %	Height	Height %
7.367	5165306	51.60	241770	51.07
8.160	4845414	48.40	231638	48.93
Totals	10010720	100.00	473408	100.00

HPLC spectrum of (*S*,*Z*)-1-(Furan-2-yl)hexadec-9-en-1-ol (18b):

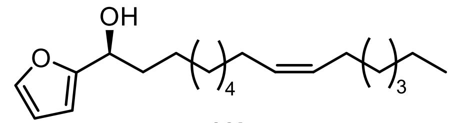

18b

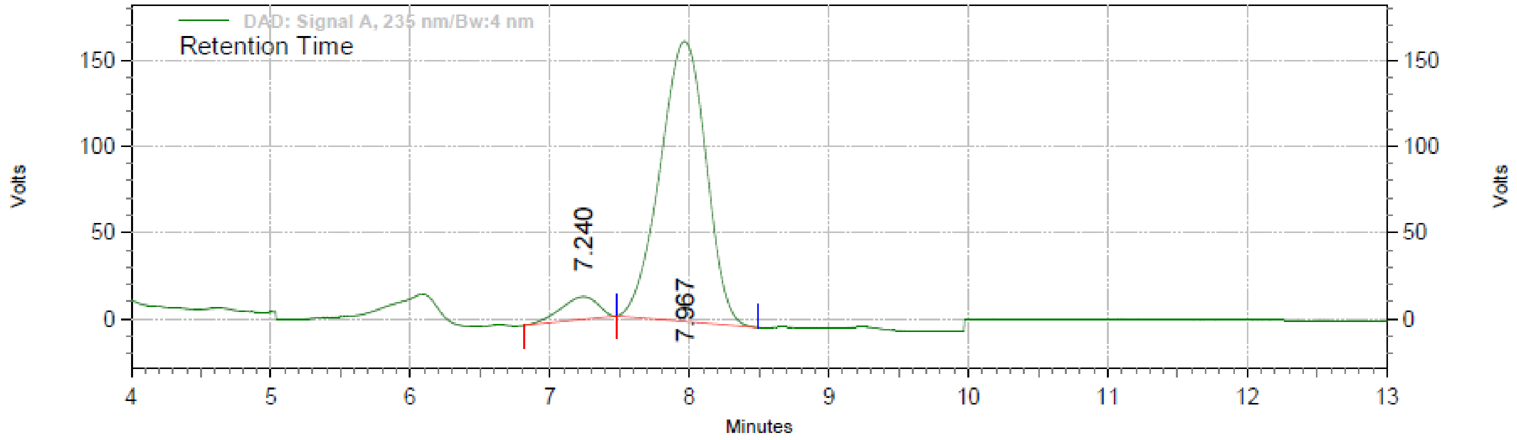

DAD: Signal A,
235 nm/Bw:4 nm
Results

Retention Time	Area	Area %	Height	Height %
7.240	506385	6.12	27370	7.45
7.967	7769481	93.88	339963	92.55
Totals	8275866	100.00	367333	100.00

^{1}H NMR spectrum of (2*S*)-6-Hydroxy-2-((*Z*)-pentadec-8-en-1-yl)-2H-pyran-3(6H)-one (19b):

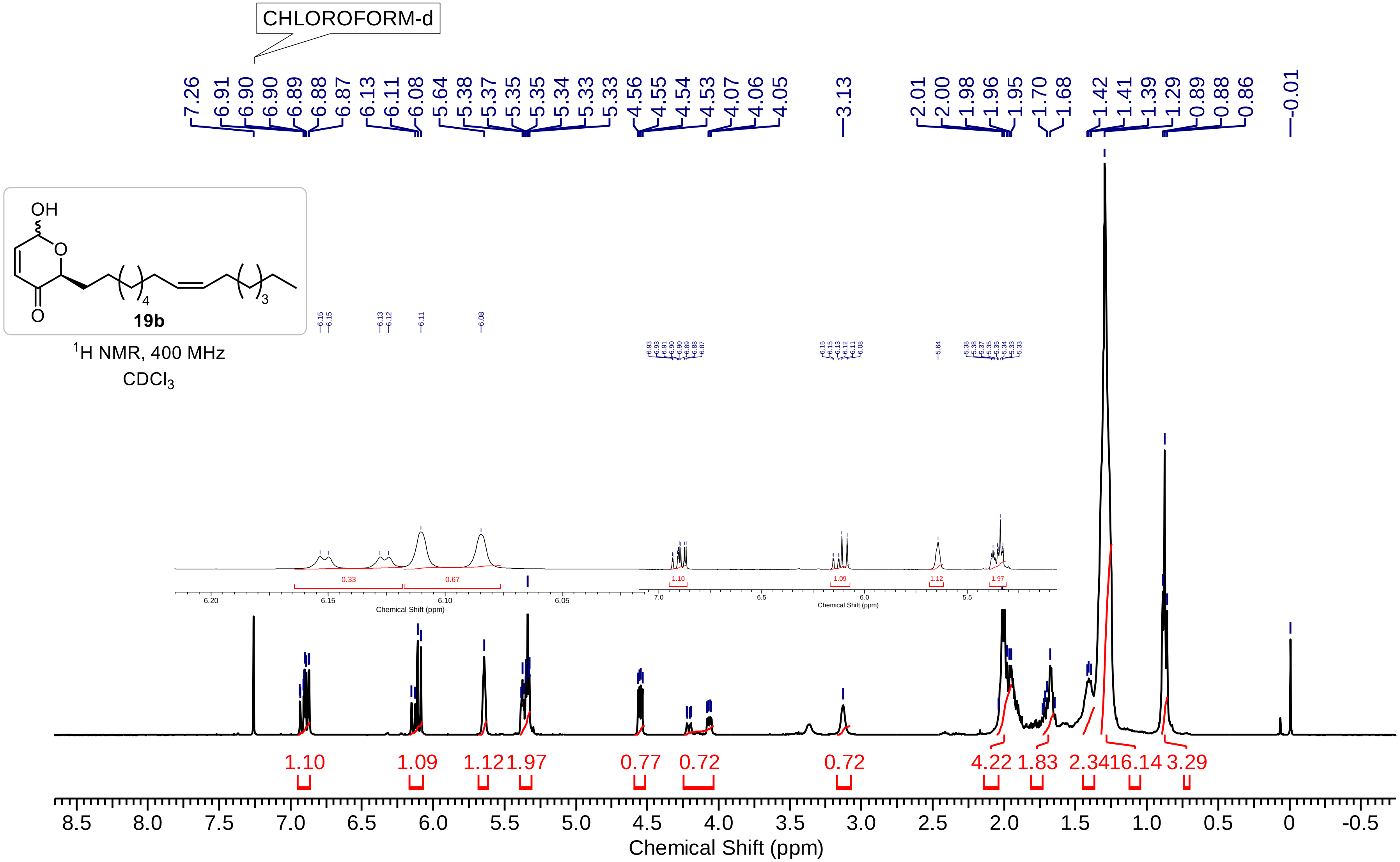

$^{13}C\{^{1}H\}$ NMR spectrum of (2*S*)-6-Hydroxy-2-((*Z*)-pentadec-8-en-1-yl)-2H-pyran-3(6H)-one (19b):

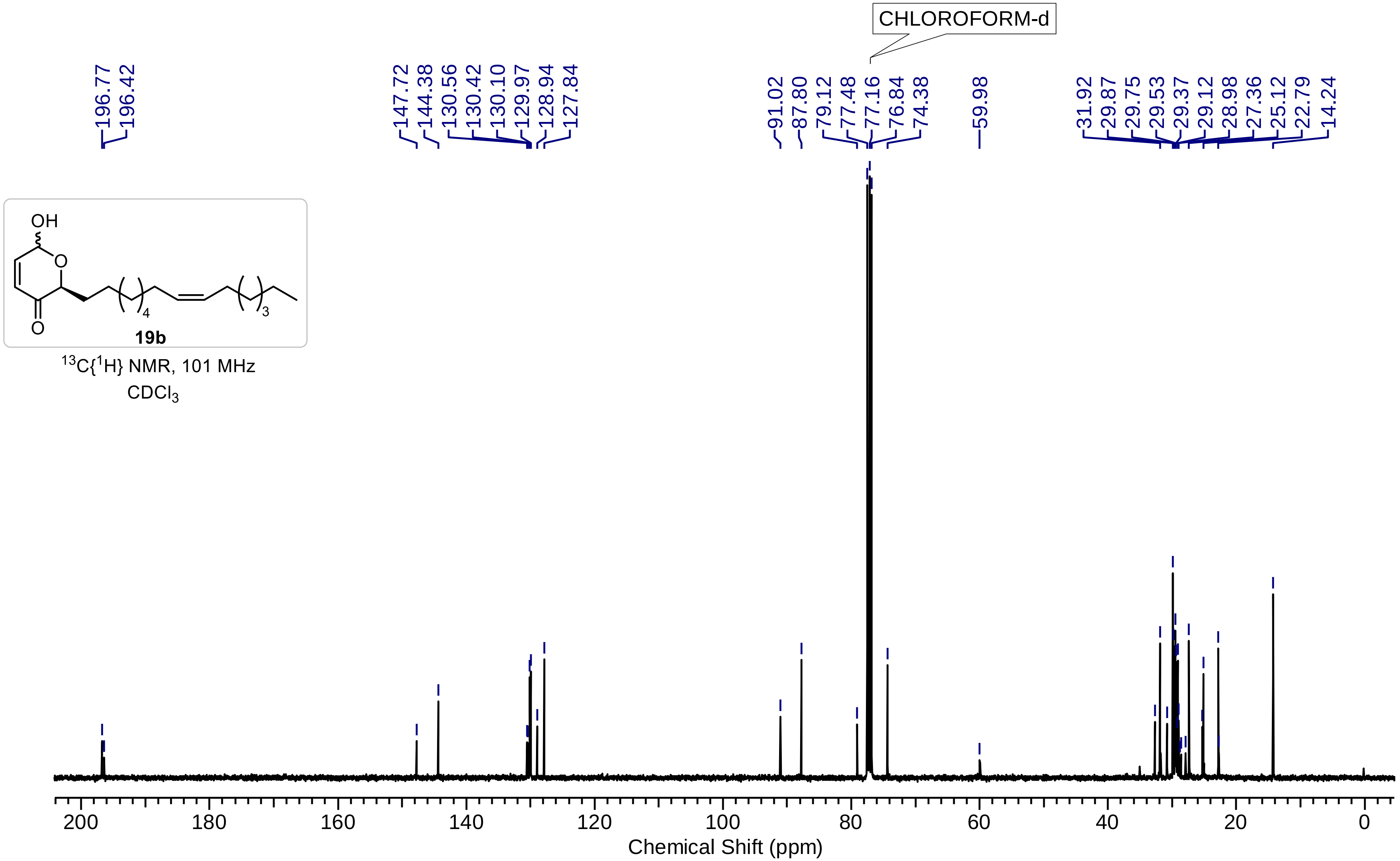

^{1}H NMR spectrum of 9-((Tert-butyldimethylsilyl)oxy)-1-(furan-2-yl)nonan-1-ol (36):

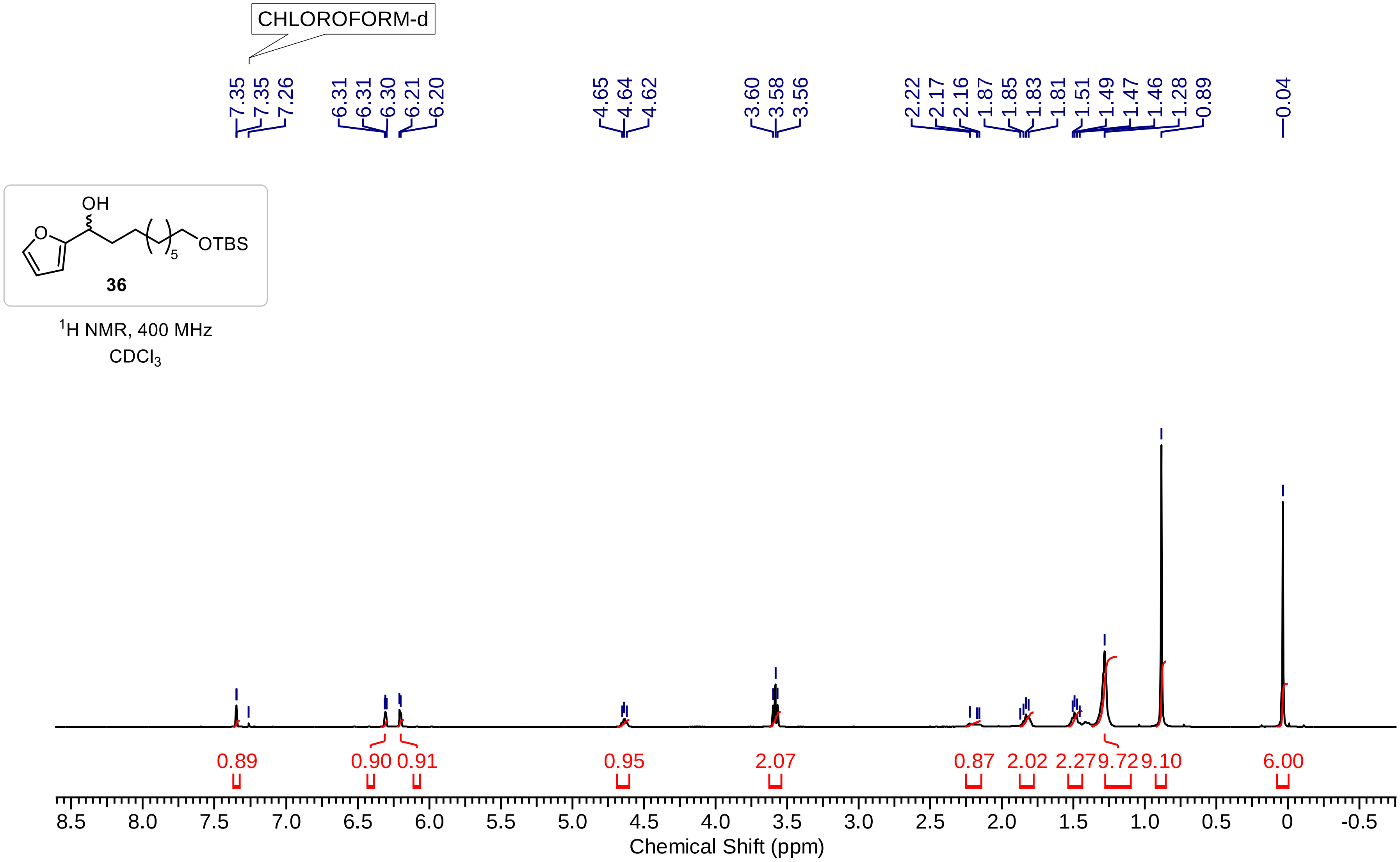

$^{13}C\{^{1}H\}$ NMR spectrum of 9-((Tert-butyldimethylsilyl)oxy)-1-(furan-2-yl)nonan-1-ol (36):

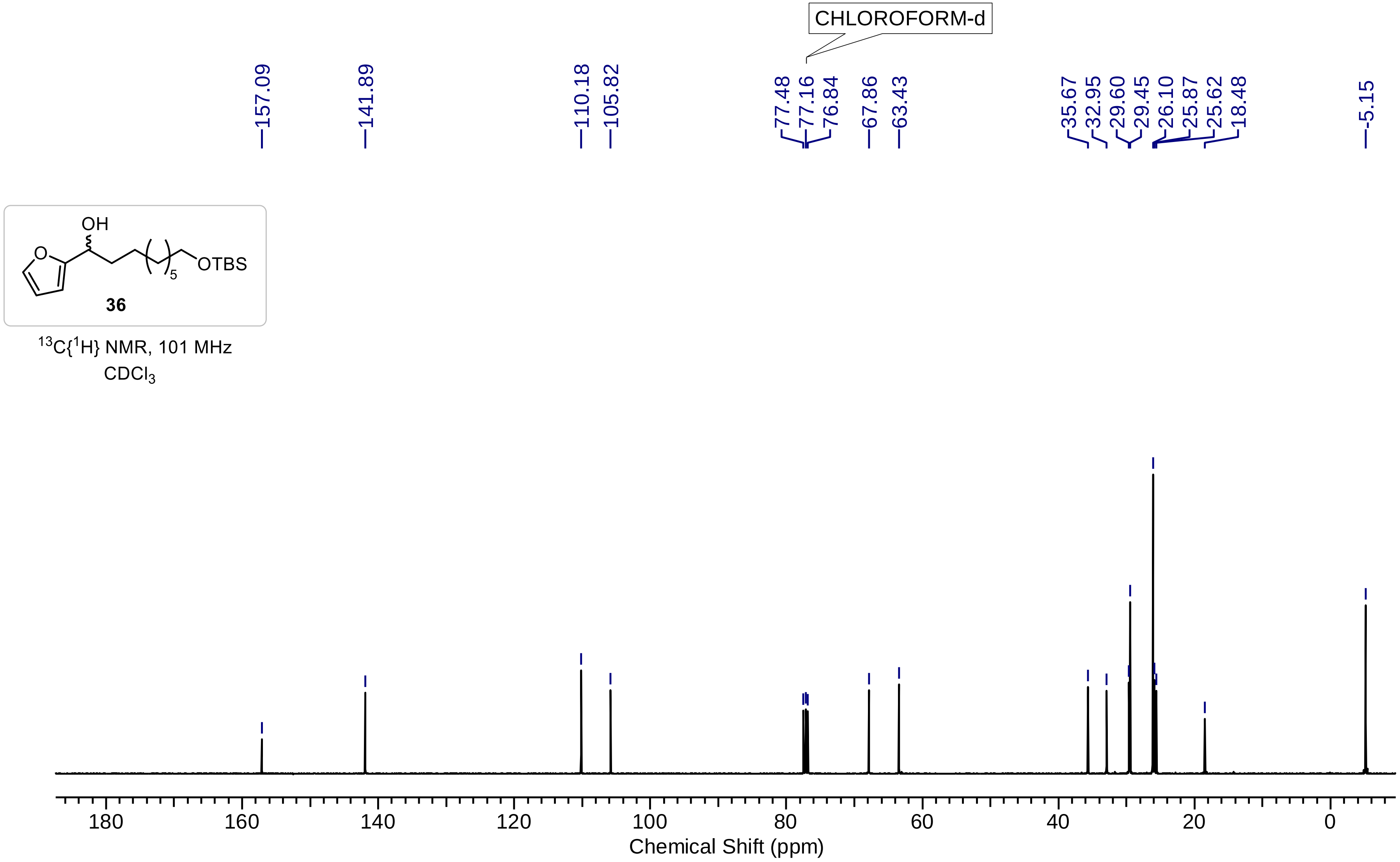

^{1}H NMR spectrum of 9-((Tert-butyldimethylsilyl)oxy)-1-(furan-2-yl)nonan-1-one (37):

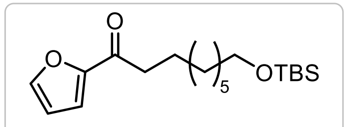

^{1}H NMR, 400 MHz
$CDCl_3$

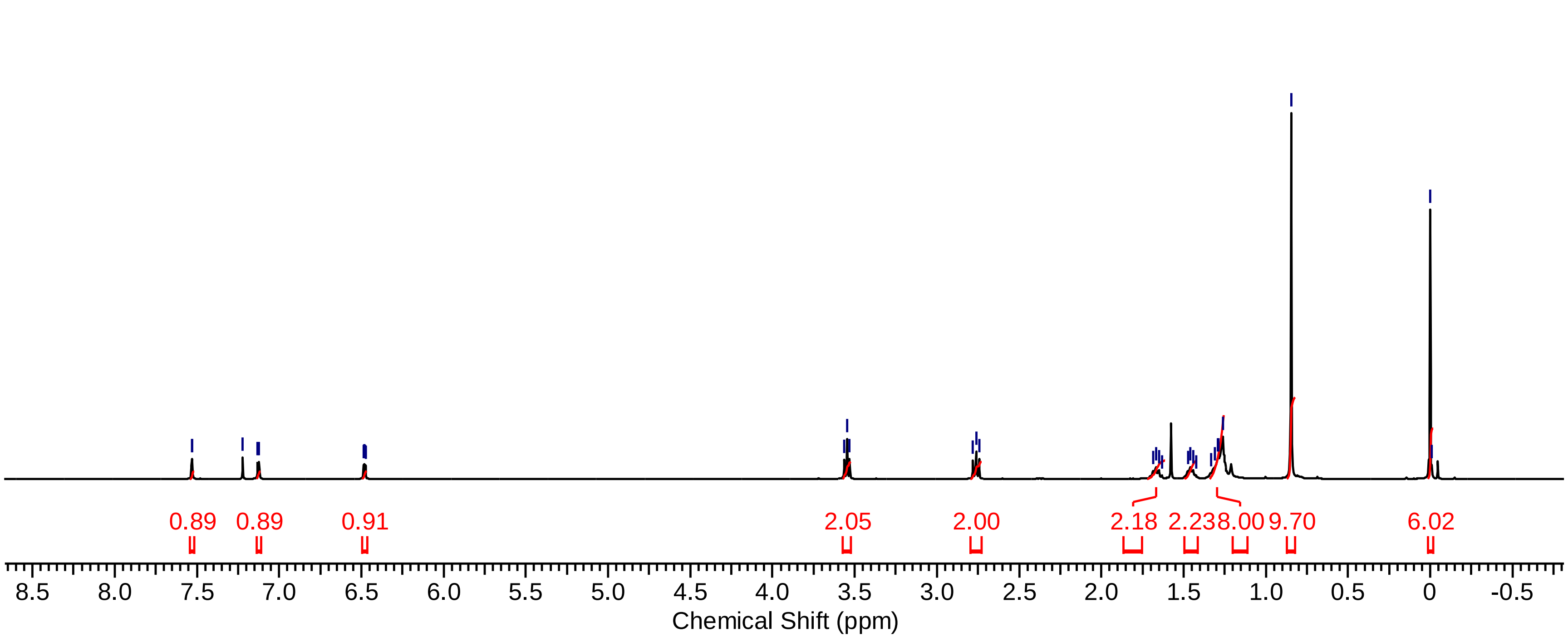

$^{13}C\{^{1}H\}$ NMR spectrum of 9-((Tert-butyldimethylsilyl)oxy)-1-(furan-2-yl)nonan-1-one (37):

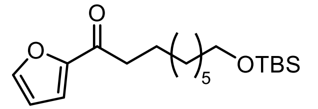

37

$^{13}C\{^{1}H\}$ NMR, 101 MHz
$CDCl_3$

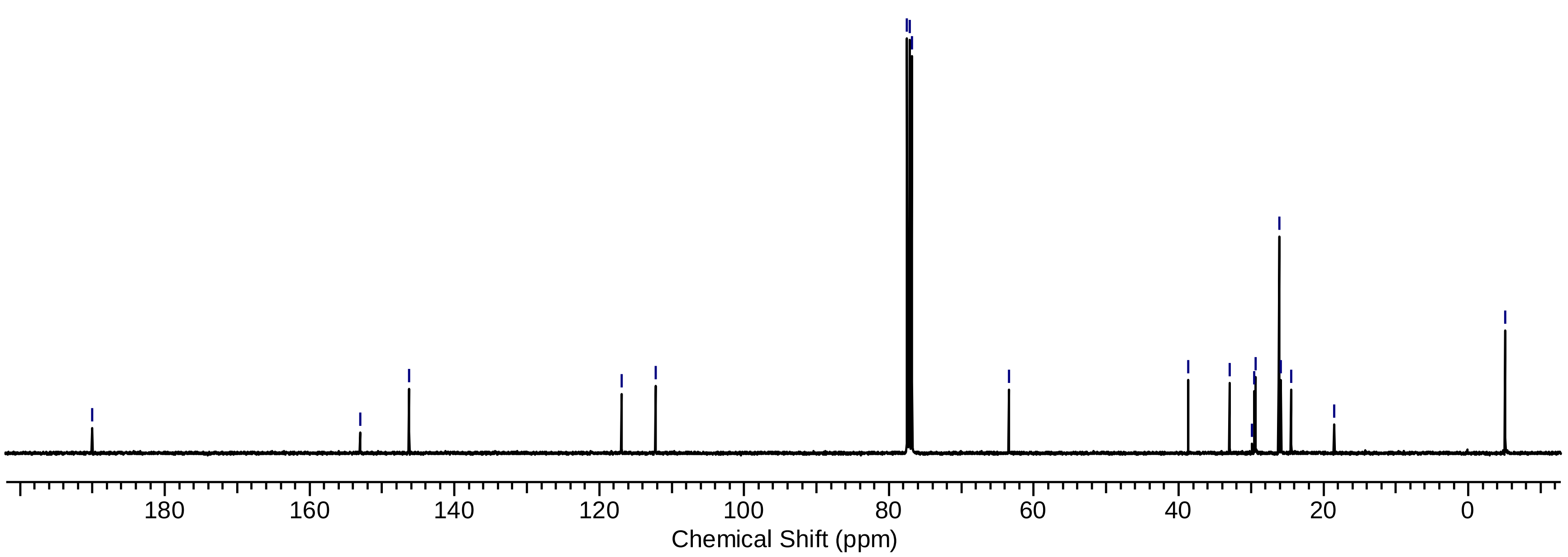

^{1}H NMR spectrum of (*S*)-9-((Tert-butyldimethylsilyl)oxy)-1-(furan-2-yl)nonan-1-ol (38):

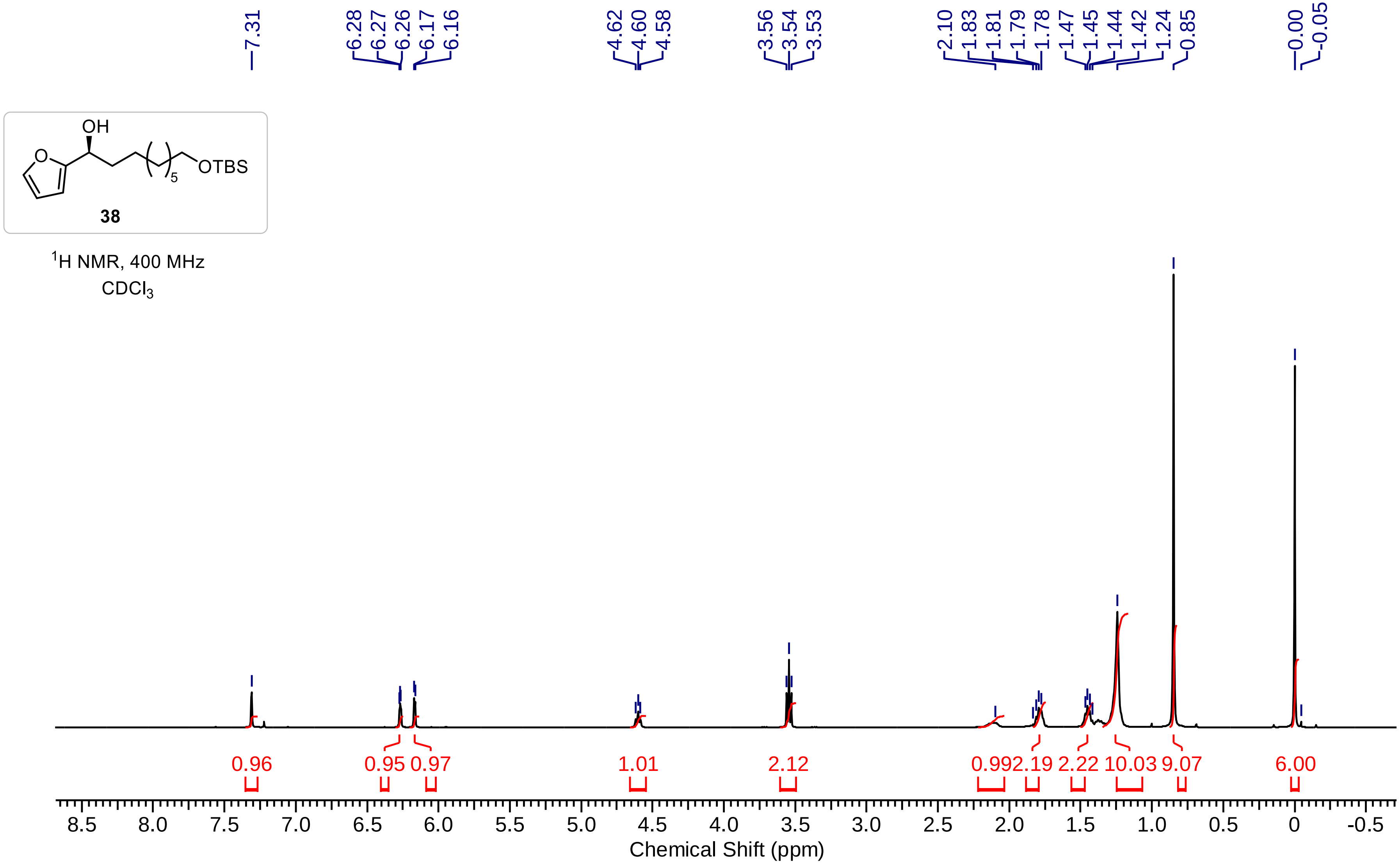

$^{13}C\{^1H\}$ NMR spectrum of (*S*)-9-((Tert-butyldimethylsilyl)oxy)-1-(furan-2-yl)nonan-1-ol (38):

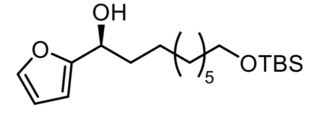

38

$^{13}C\{^1H\}$ NMR, 101 MHz
$CDCl_3$

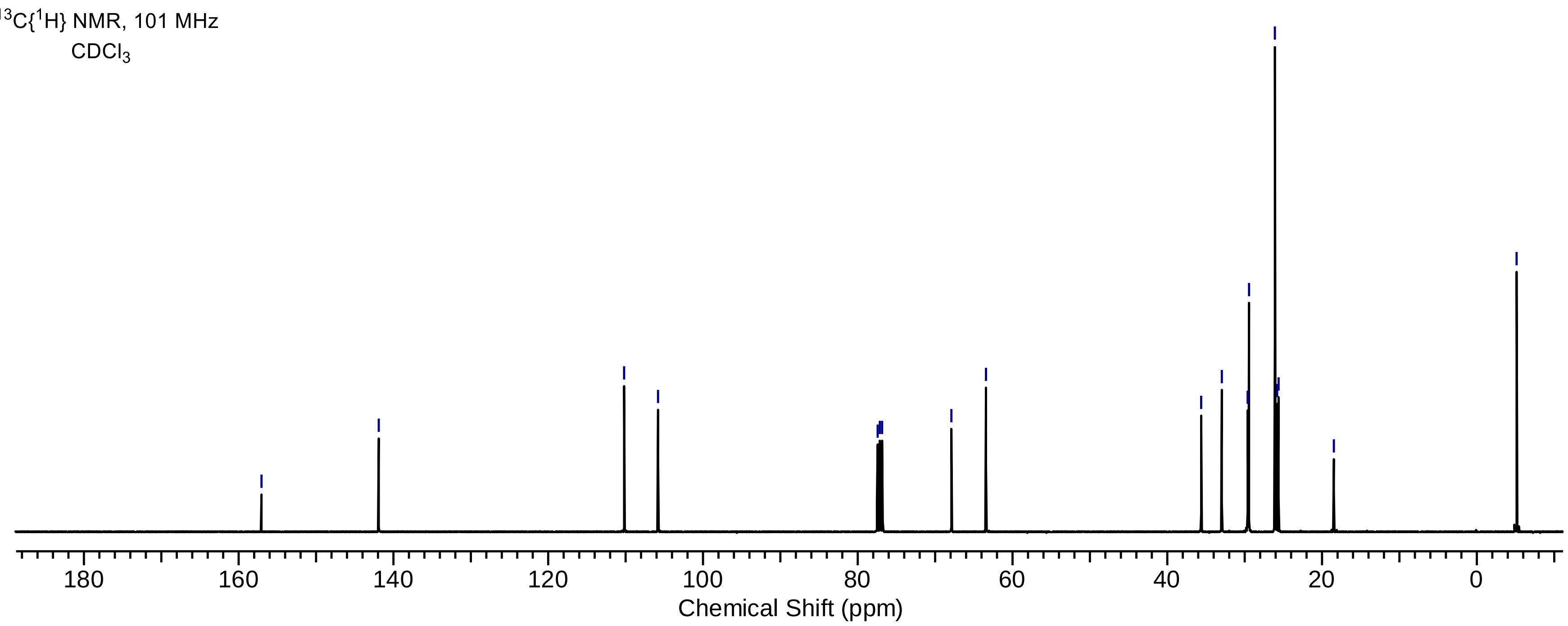

HPLC spectrum of (±) 9-((Tert-butyldimethylsilyl)oxy)-1-(furan-2-yl)nonan-1-ol (36):

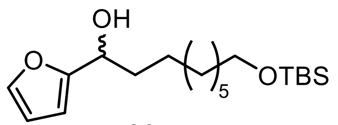

36

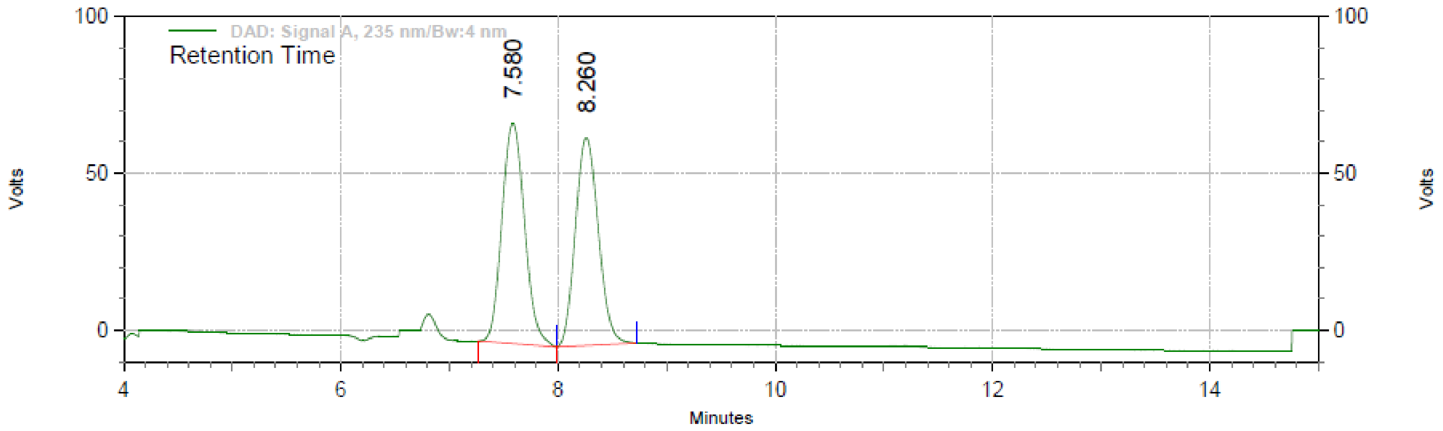

DAD: Signal A,
235 nm/Bw:4 nm
Results

Retention Time	Area	Area %	Height	Height %
7.580	2132913	51.13	146676	51.44
8.260	2038342	48.87	138450	48.56
Totals	4171255	100.00	285126	100.00

HPLC spectrum of (*S*)-9-((Tert-butyldimethylsilyl)oxy)-1-(furan-2-yl)nonan-1-ol (38):

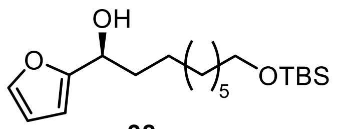

38

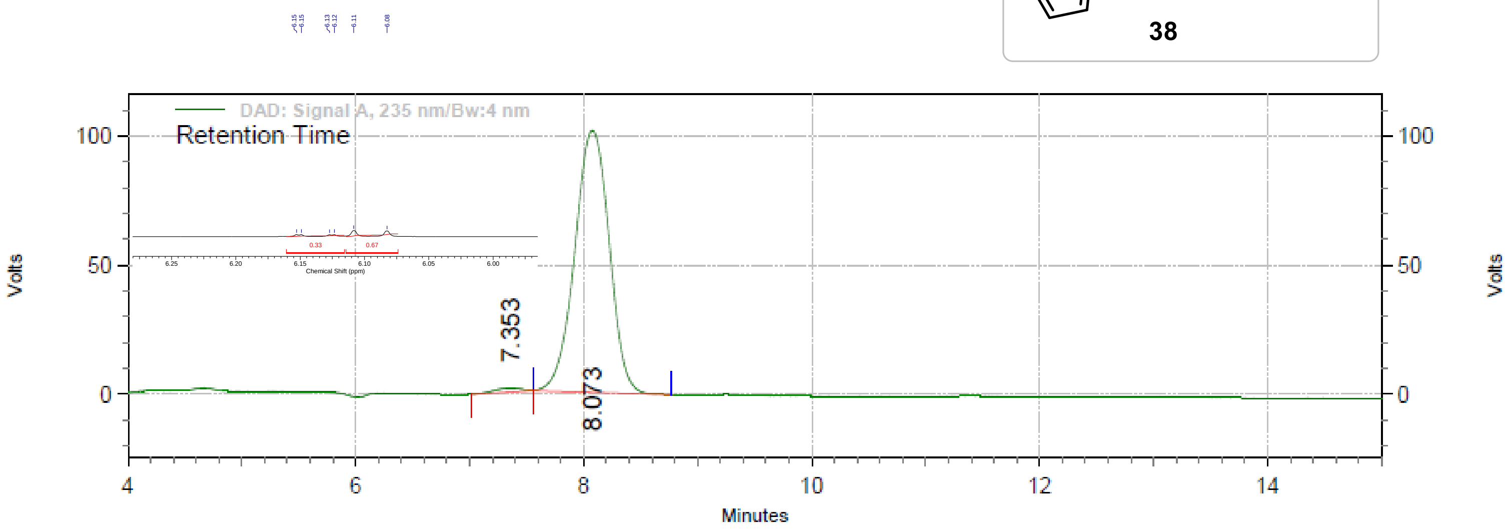

DAD: Signal A,
235 nm/Bw:4 nm
Results

Retention Time	Area	Area %	Height	Height %
7.353	50226	1.11	3105	1.44
8.073	4465481	98.89	212823	98.56
Totals	4515707	100.00	215928	100.00

^{1}H NMR spectrum of (2*S*)-2-(8-((Tert-butyldimethylsilyl)oxy)octyl)-6-hydroxy-2H-pyran-3(6H)-one (39):

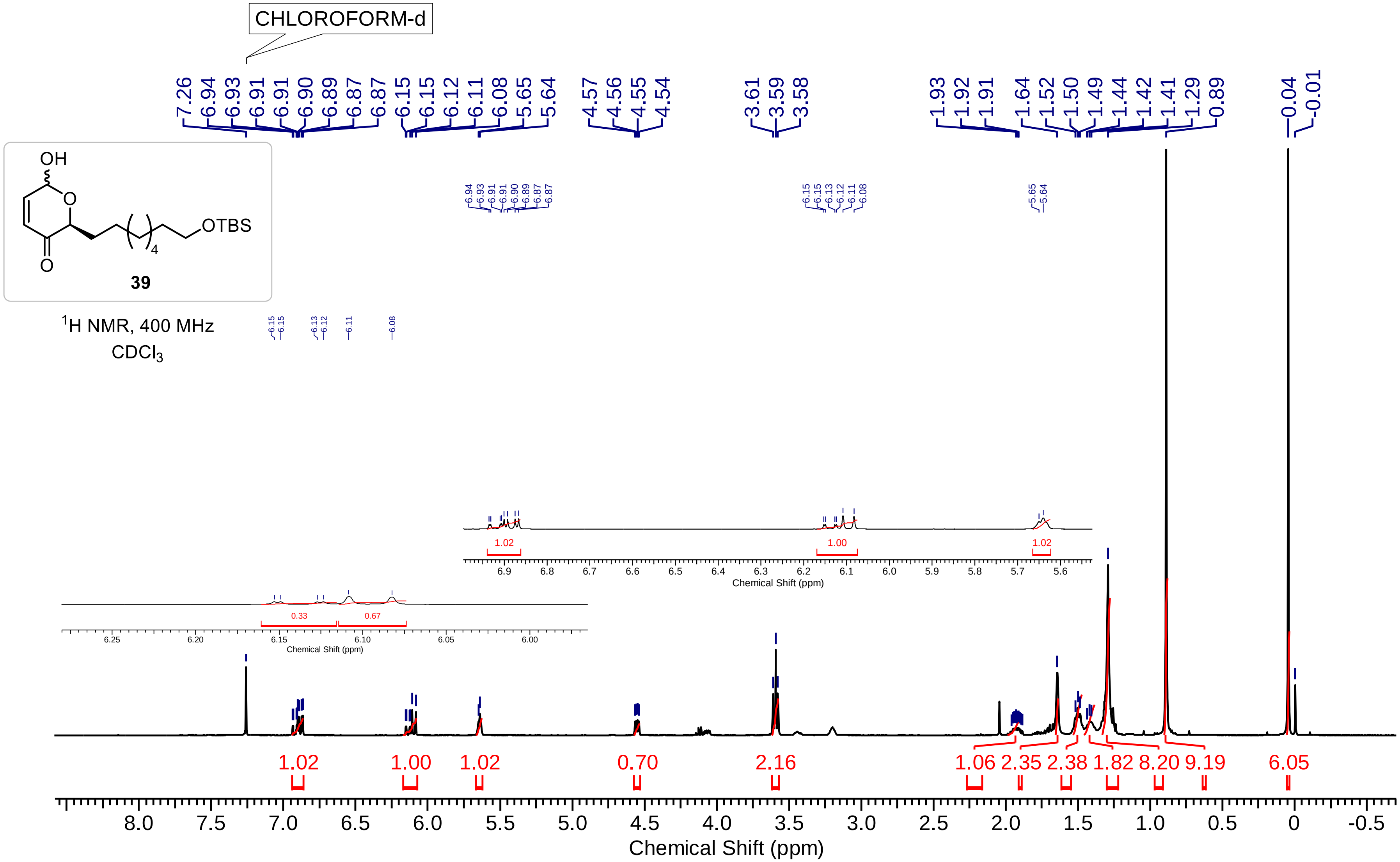

$^{13}C\{^1H\}$ NMR spectrum of (2*S*)-2-(8-((Tert-butyldimethylsilyl)oxy)octyl)-6-hydroxy-2H-pyran-3(6H)-one (39):

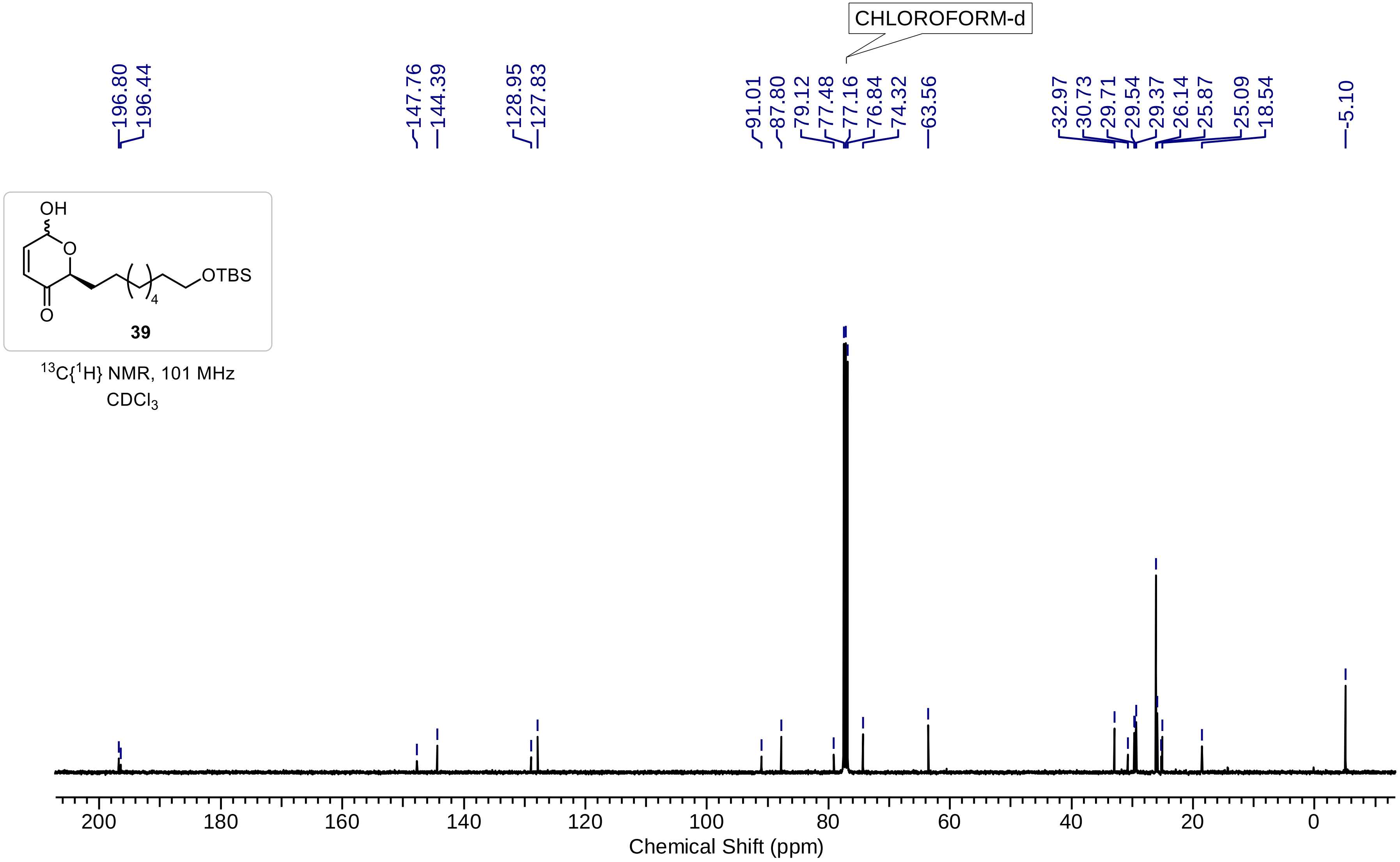

^{1}H NMR spectrum of (5*S*,6*S*)-6-(8-((Tert-butyldimethylsilyl)oxy)octyl)-5-hydroxy-5,6-dihydro-2H-pyran-2-one (40):

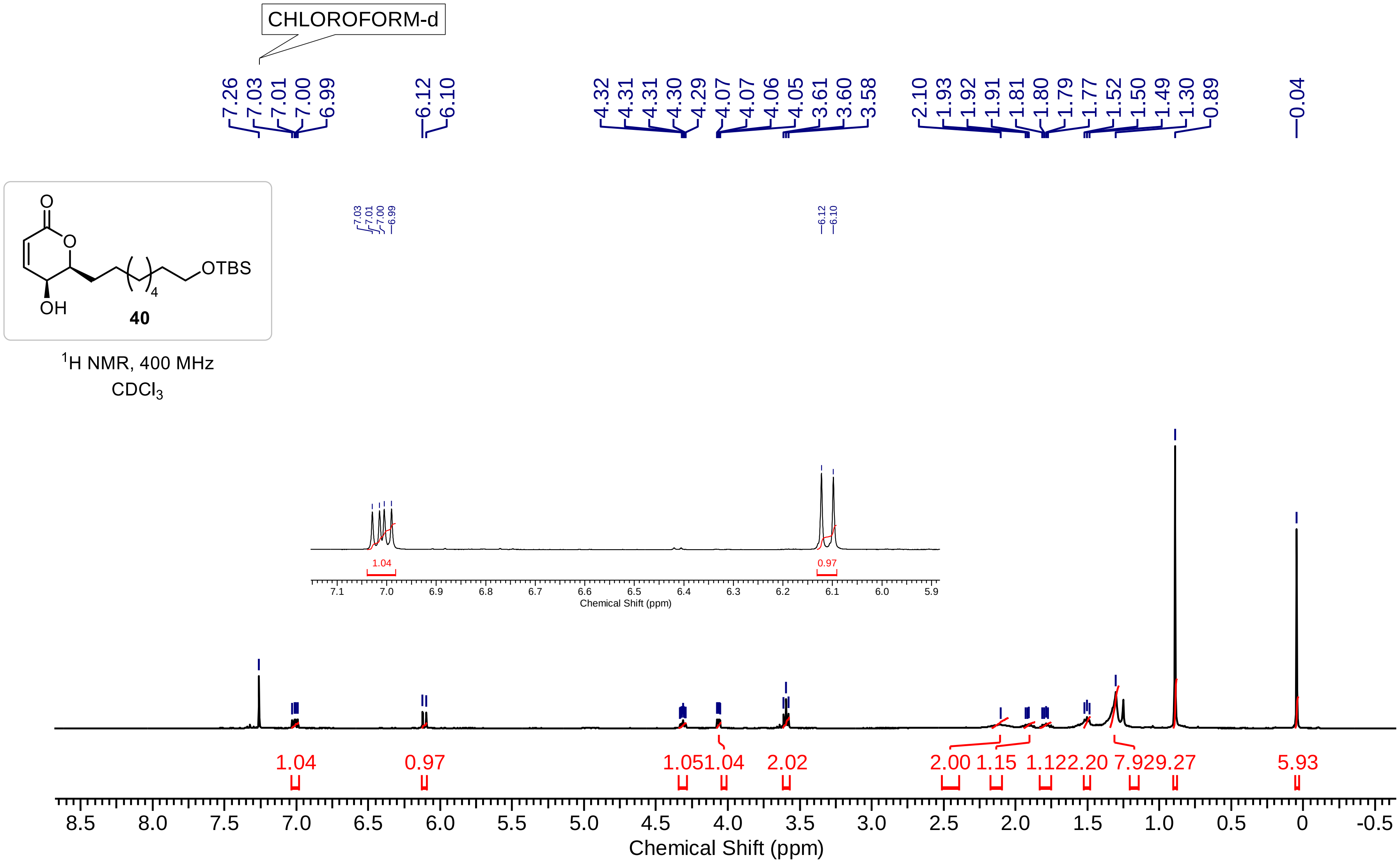

$^{13}C\{^1H\}$ NMR spectrum of (5*S*,6*S*)-6-(8-((Tert-butyldimethylsilyl)oxy)octyl)-5-hydroxy-5,6-dihydro-2H-pyran-2-one (40):

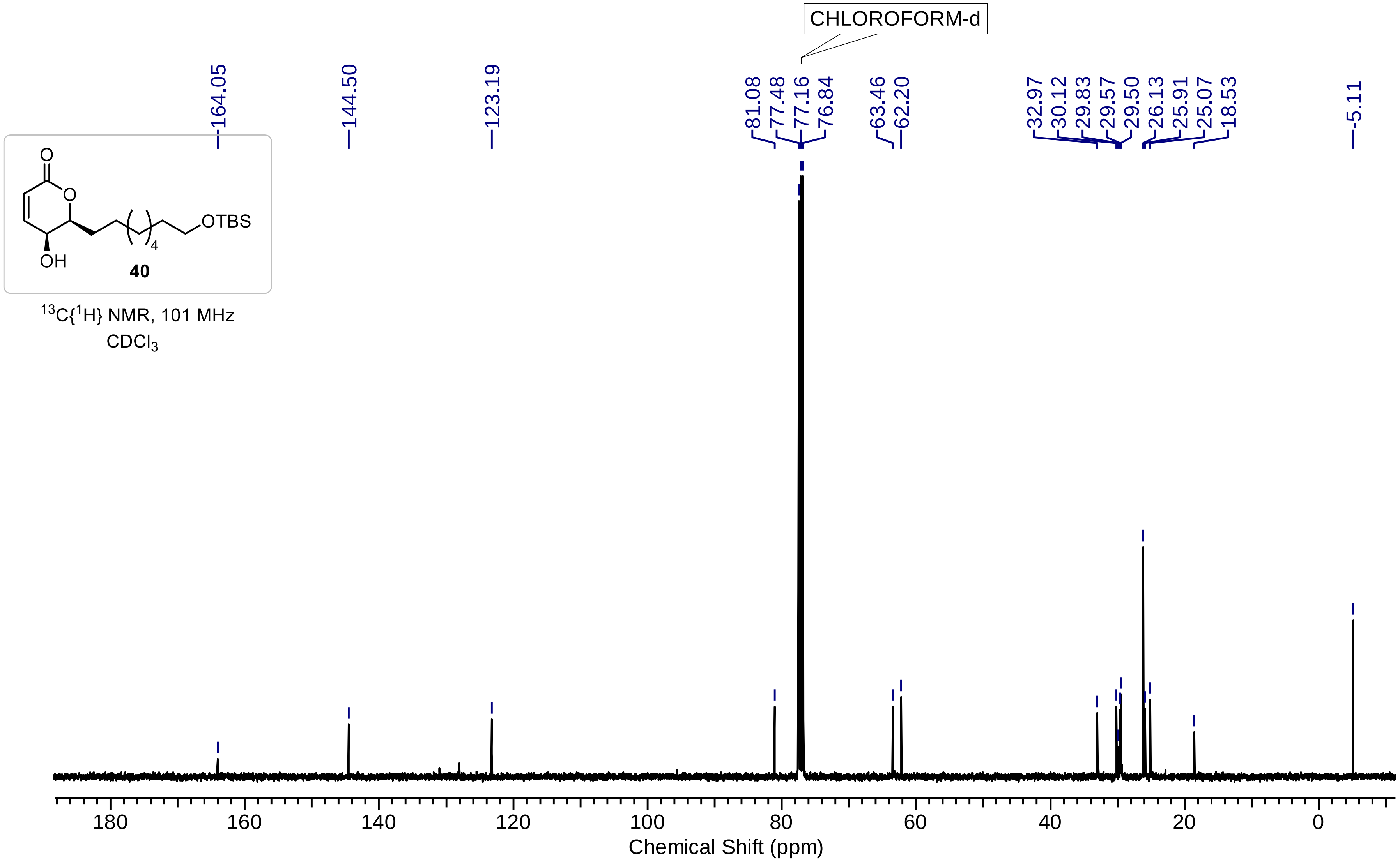

^{1}H NMR spectrum of (2*S*,3*S*)-2-(8-((Tert-butyldimethylsilyl)oxy)octyl)-6-oxo-3,6-dihydro-2H-pyran-3-yl acetate (41):

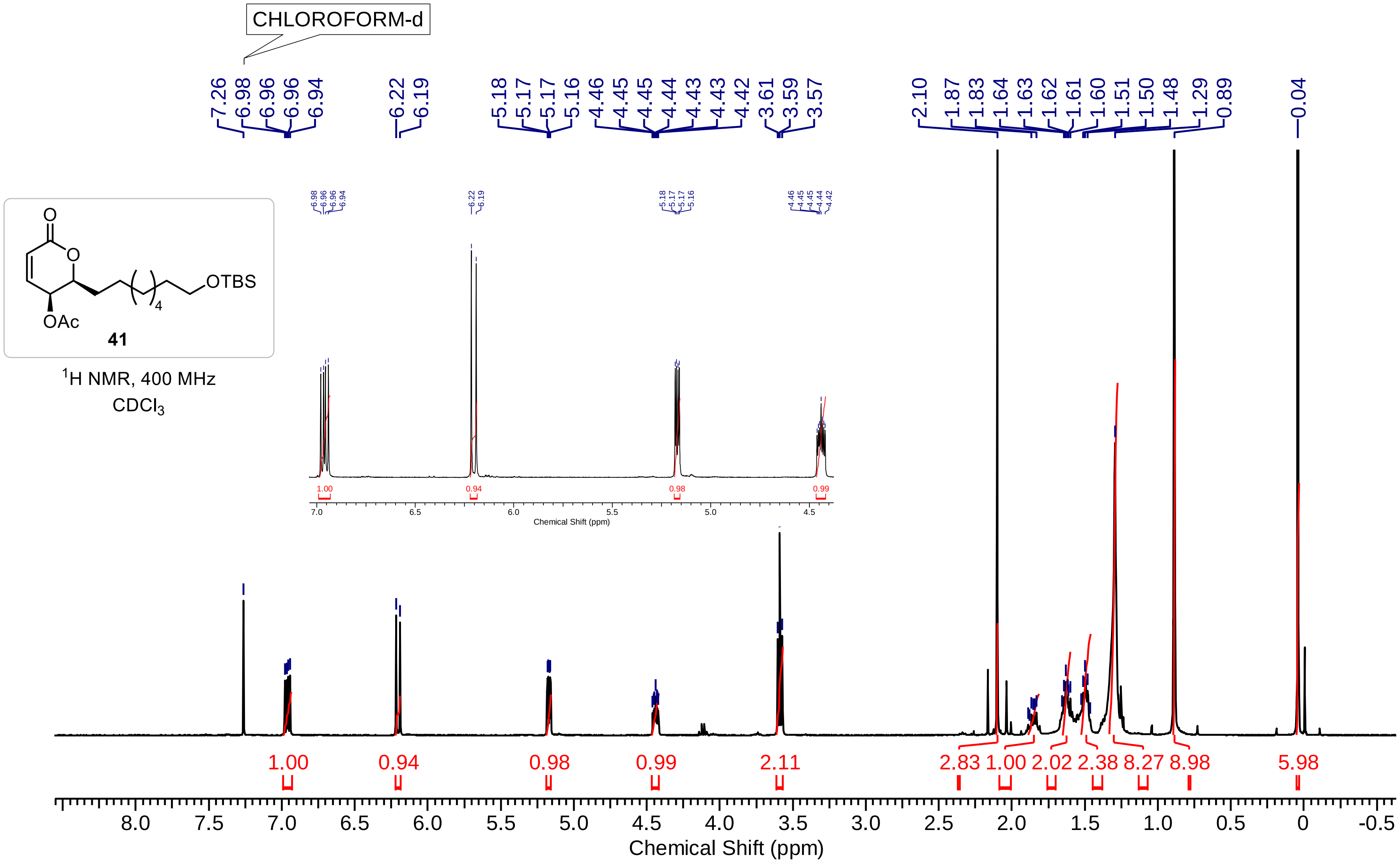

$^{13}C\{^{1}H\}$ NMR spectrum of (2*S*,3*S*)-2-(8-((Tert-butyldimethylsilyl)oxy)octyl)-6-oxo-3,6-dihydro-2H-pyran-3-yl acetate (41):

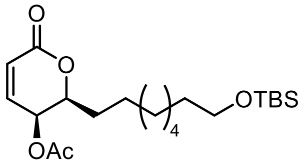

41

$^{13}C\{^{1}H\}$ NMR, 101 MHz
$CDCl_3$

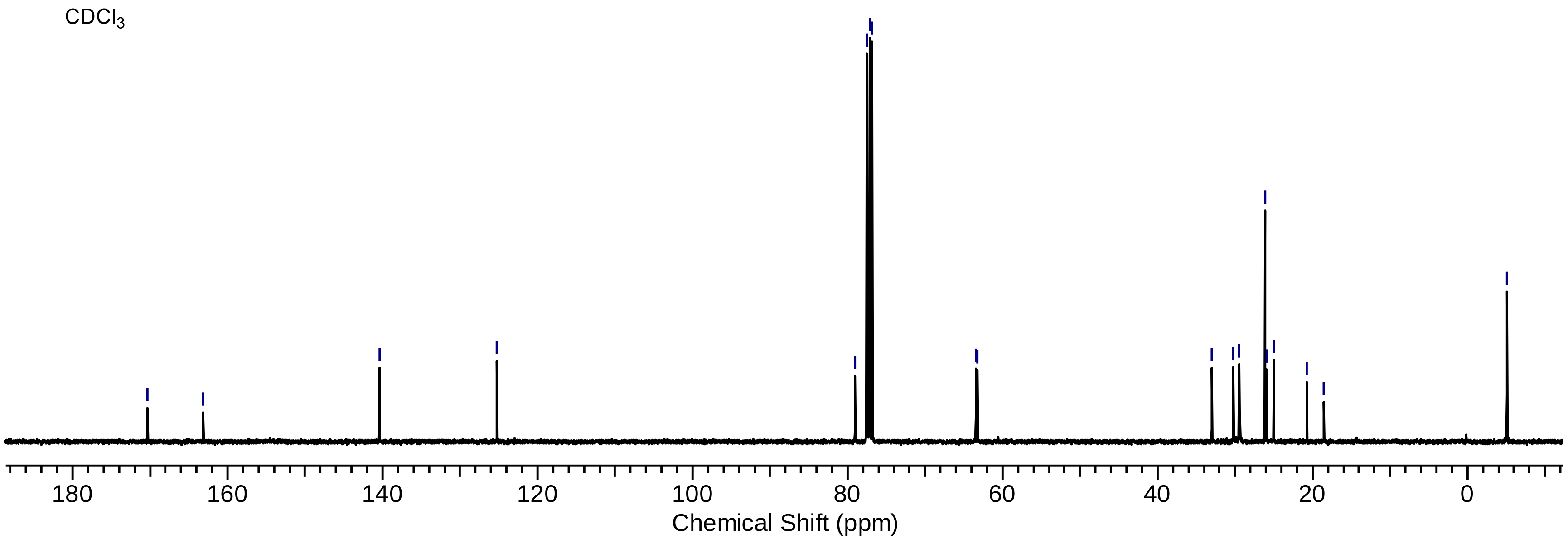

^{1}H NMR spectrum of (*S*)-6-(8-((Tert-butyldimethylsilyl)oxy)octyl)-3,6-dihydro-2H-pyran-2-one (S5):

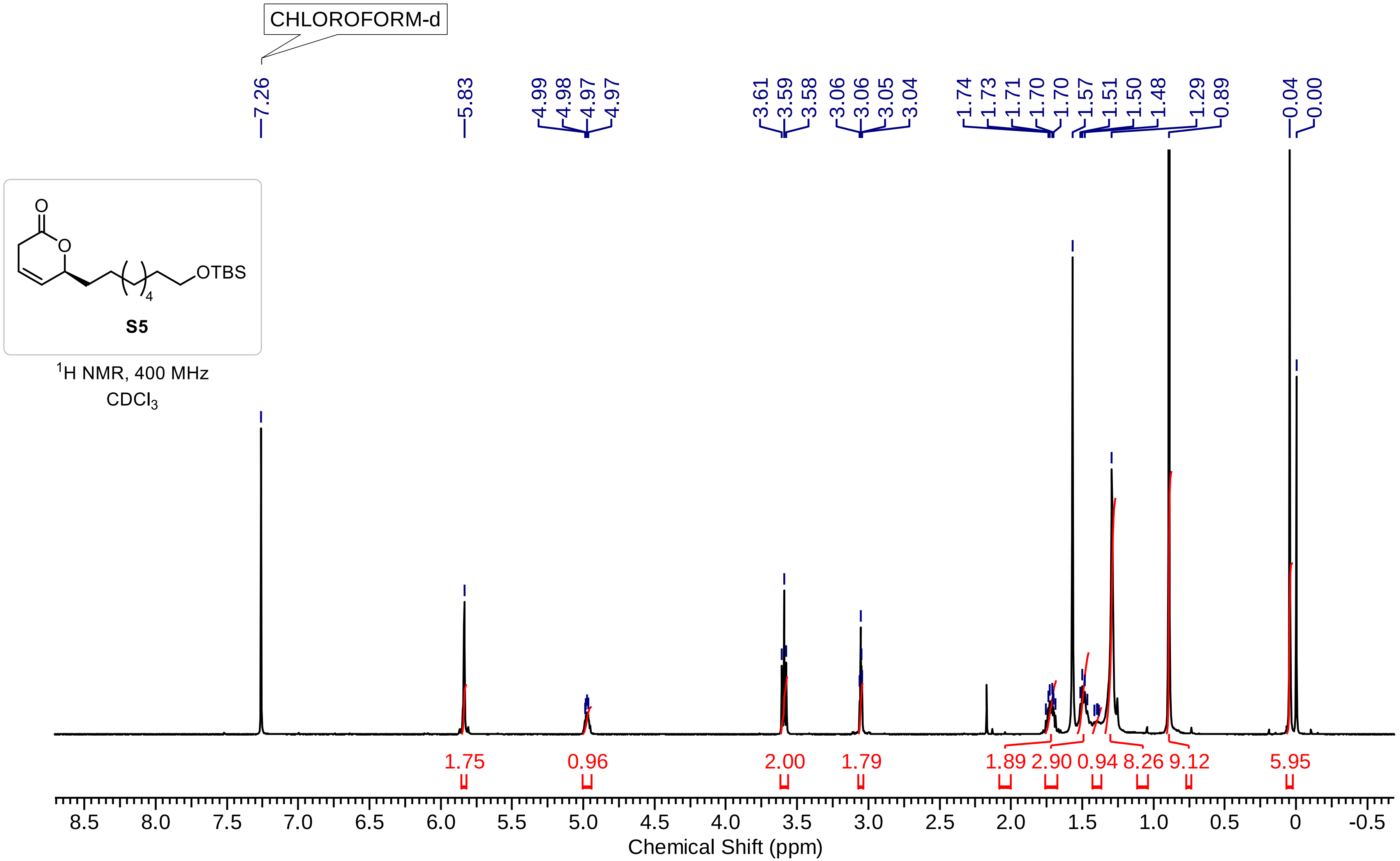

$^{13}C\{^{1}H\}$ NMR spectrum of (*S*)-6-(8-((Tert-butyldimethylsilyl)oxy)octyl)-3,6-dihydro-2H-pyran-2-one (S5):

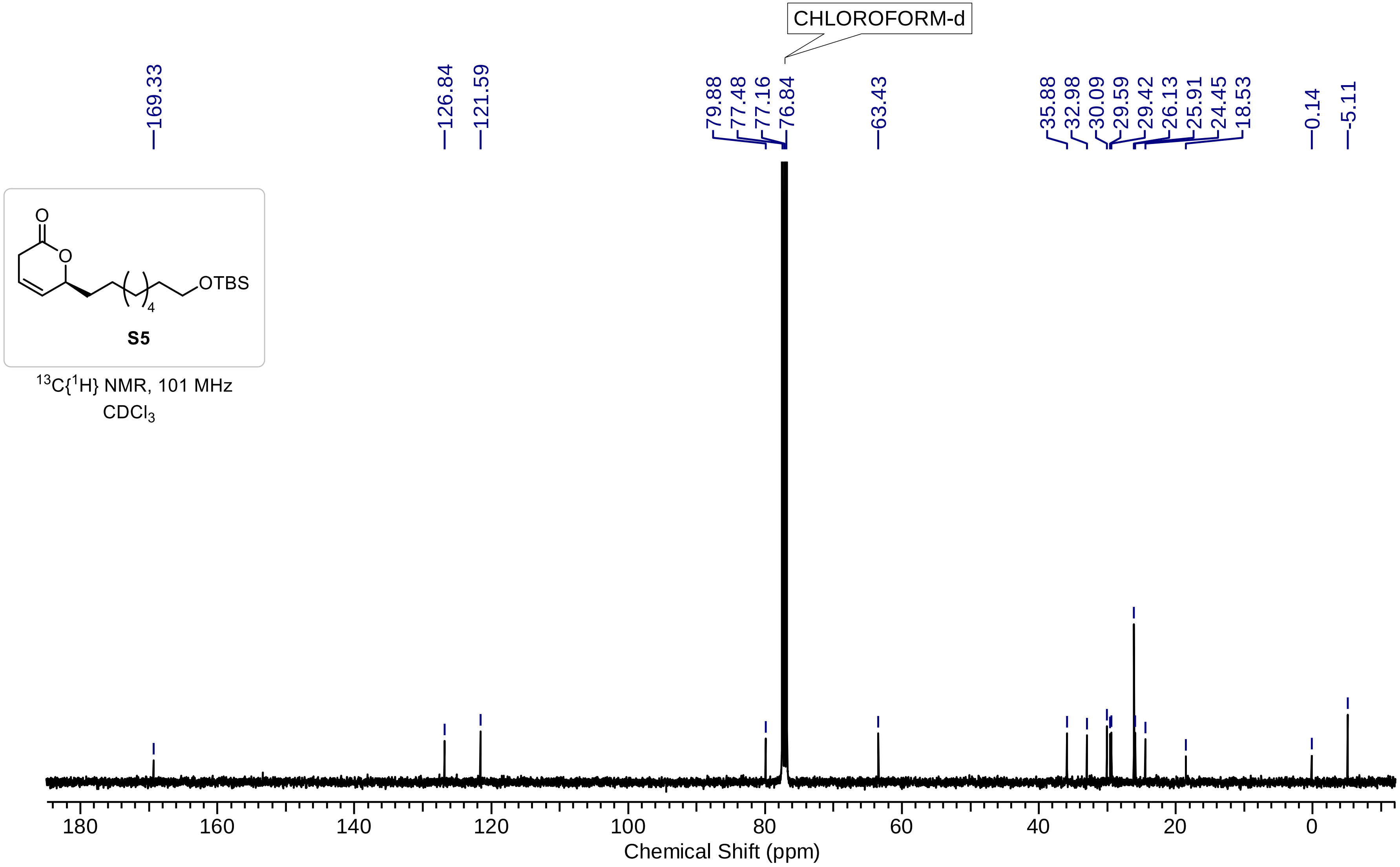

$^{13}C\{^{1}H\}$ NMR, 101 MHz
$CDCl_3$

^{1}H NMR spectrum of (*S*)-6-(8-((Tert-butyldimethylsilyl)oxy)octyl)-5,6-dihydro-2H-pyran-2-one (42):

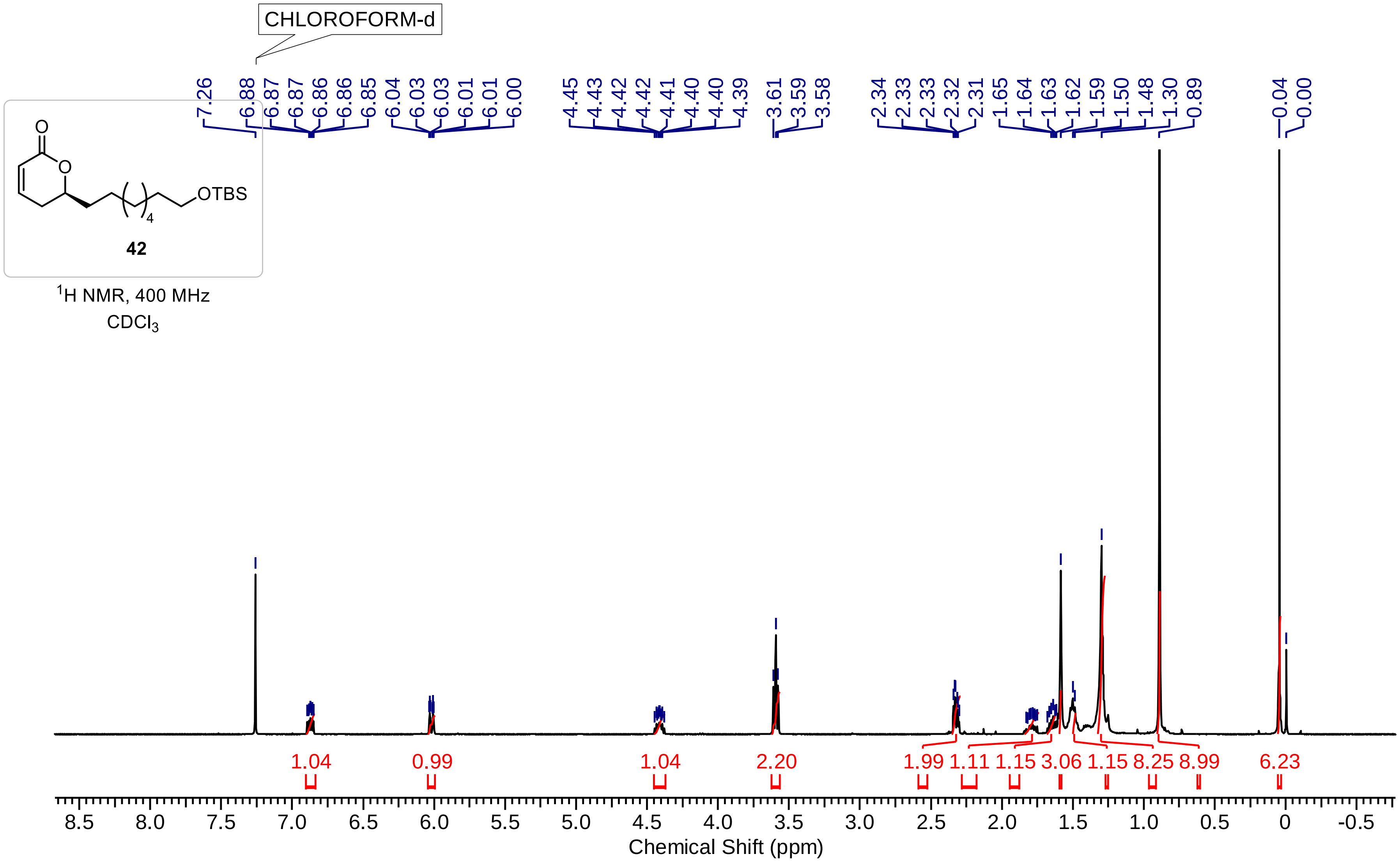

$^{13}C\{^{1}H\}$ NMR spectrum of (*S*)-6-(8-((Tert-butyldimethylsilyl)oxy)octyl)-5,6-dihydro-2H-pyran-2-one (42):

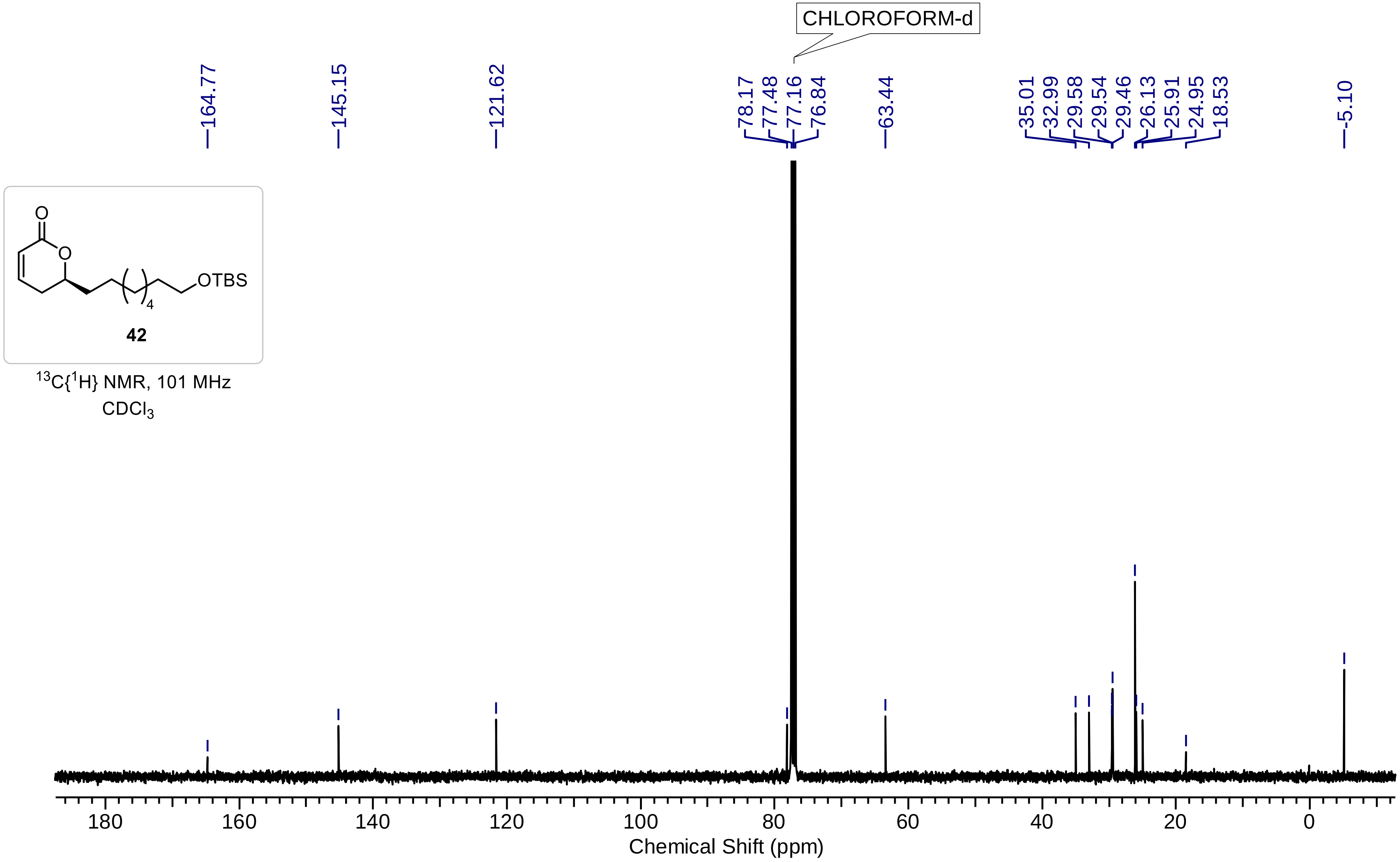

42

$^{13}C\{^{1}H\}$ NMR, 101 MHz
$CDCl_3$

^{1}H NMR spectrum of (*S*)-6-(8-Hydroxyoctyl)-5,6-dihydro-2H-pyran-2-one (S6):

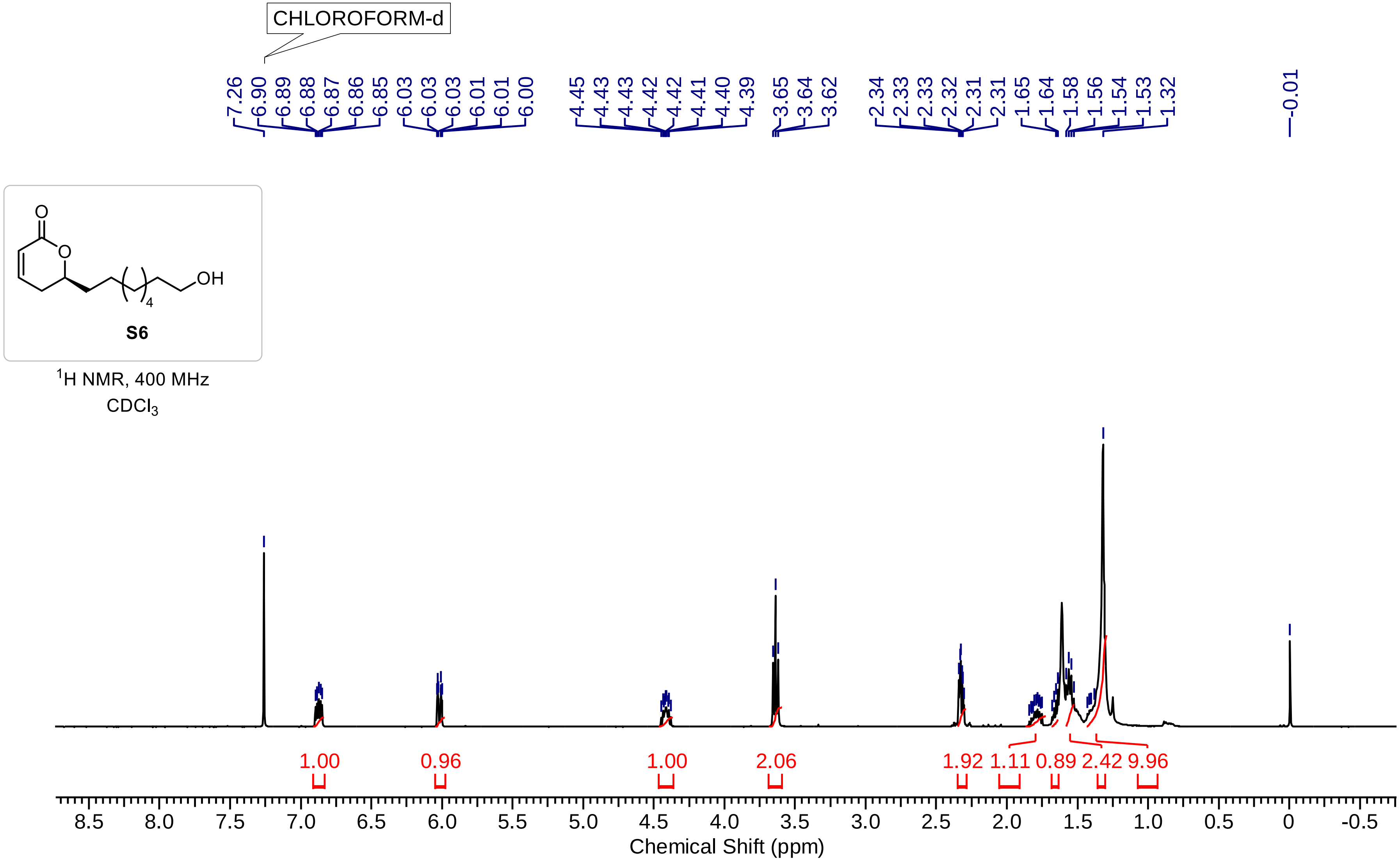

$^{13}C\{^1H\}$ NMR spectrum of (*S*)-6-(8-Hydroxyoctyl)-5,6-dihydro-2H-pyran-2-one (S6):

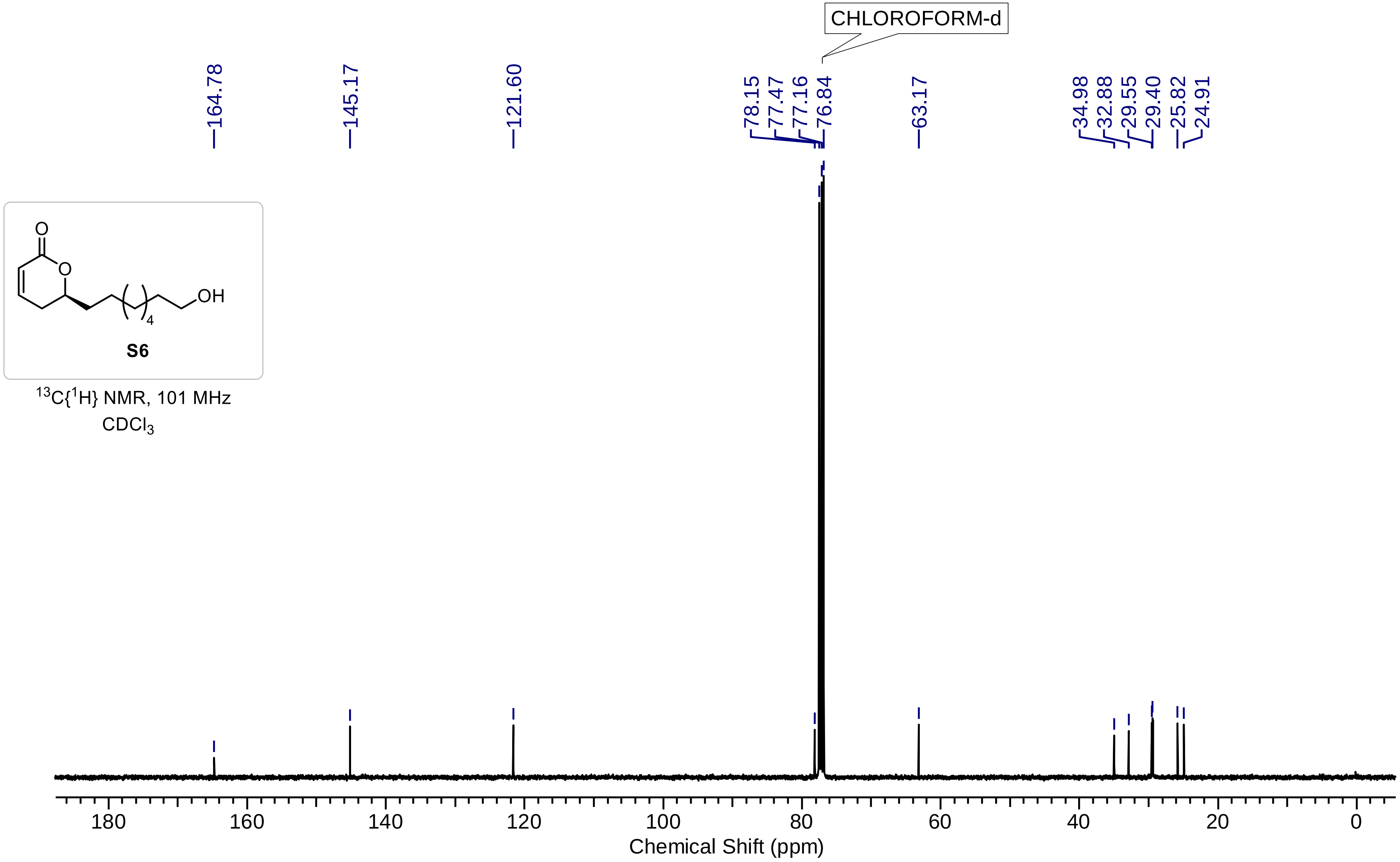

¹H NMR spectrum of (*S*)-8-(6-Oxo-3,6-dihydro-2H-pyran-2-yl)octanal (43):

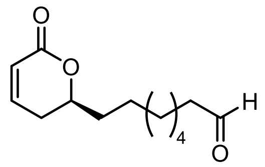

43

¹H NMR, 400 MHz
$CDCl_3$

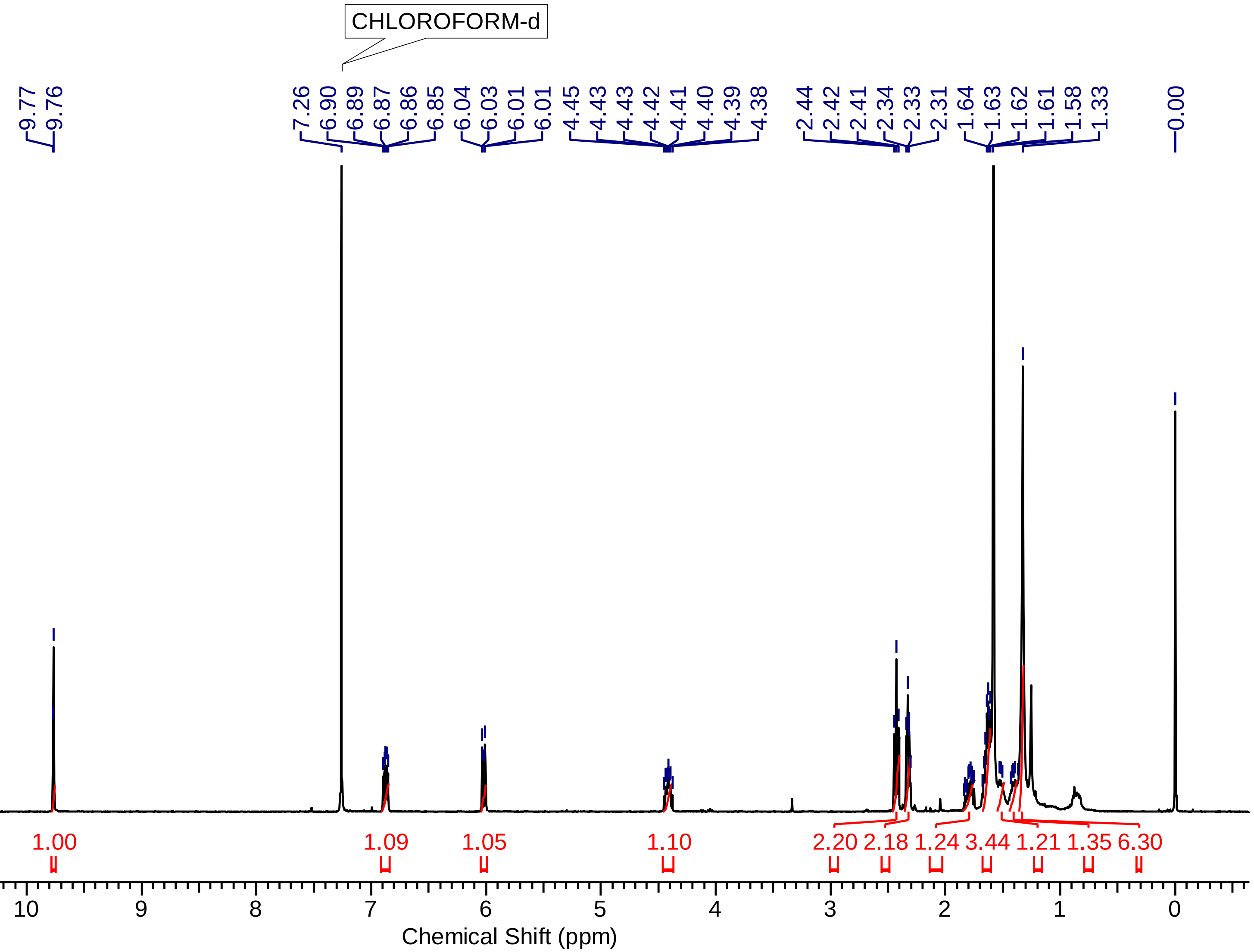

^{13}C{^{1}H} NMR spectrum of (*S*)-8-(6-Oxo-3,6-dihydro-2H-pyran-2-yl)octanal (43):

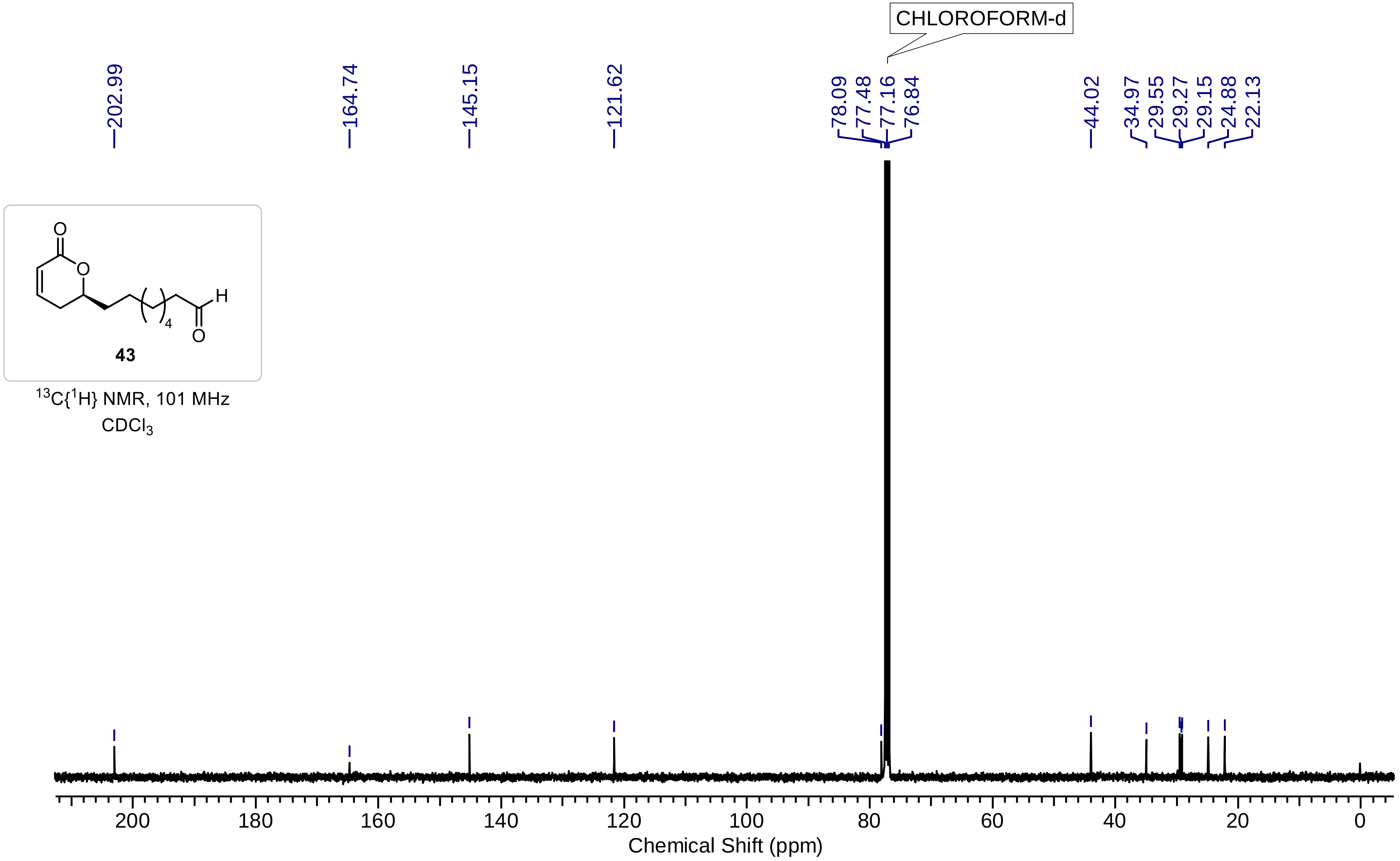

^{1}H NMR spectrum of Passifetilactone A (1):

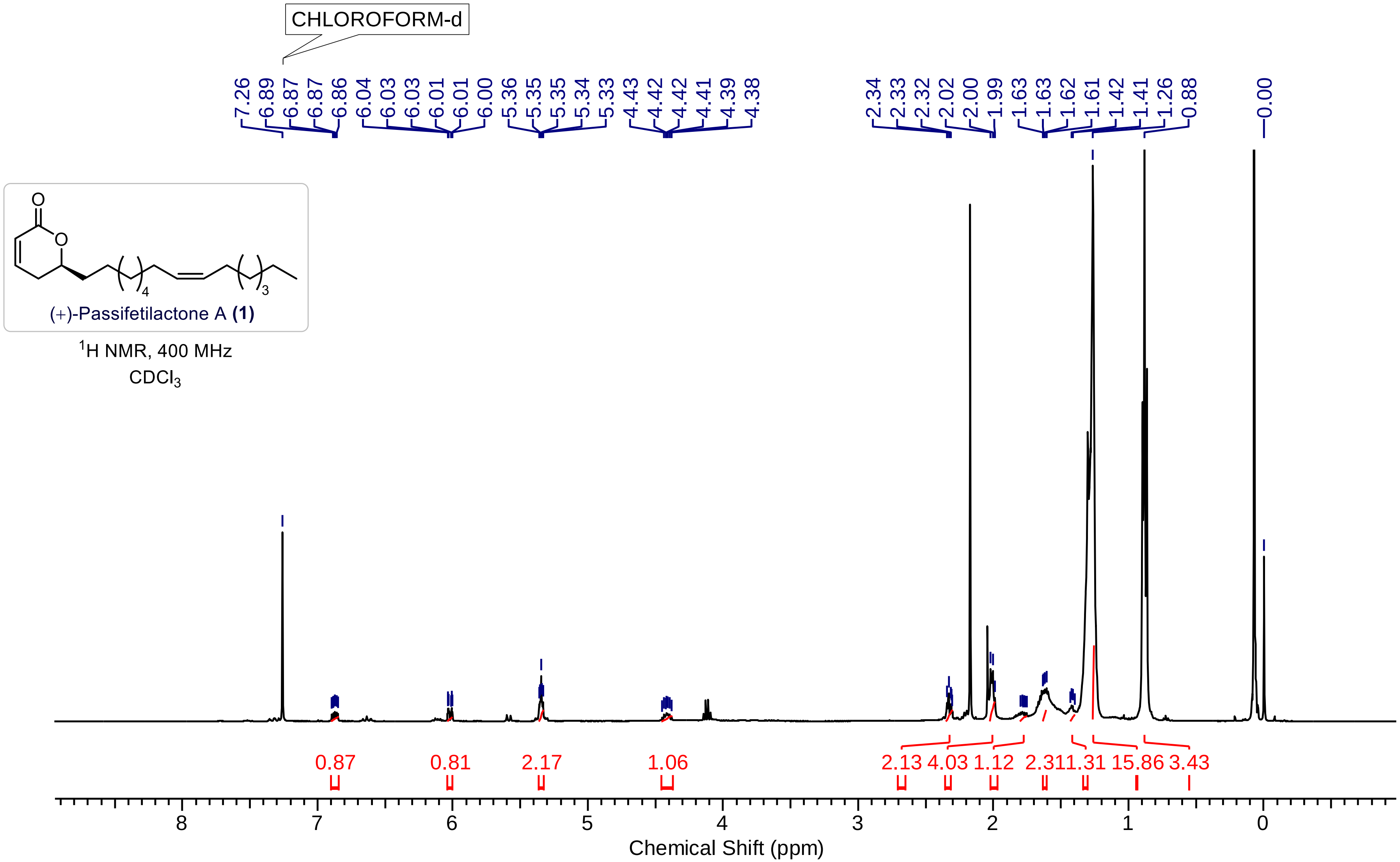

$^{13}C\{^{1}H\}$ NMR spectrum of Passifetilactone A (1):

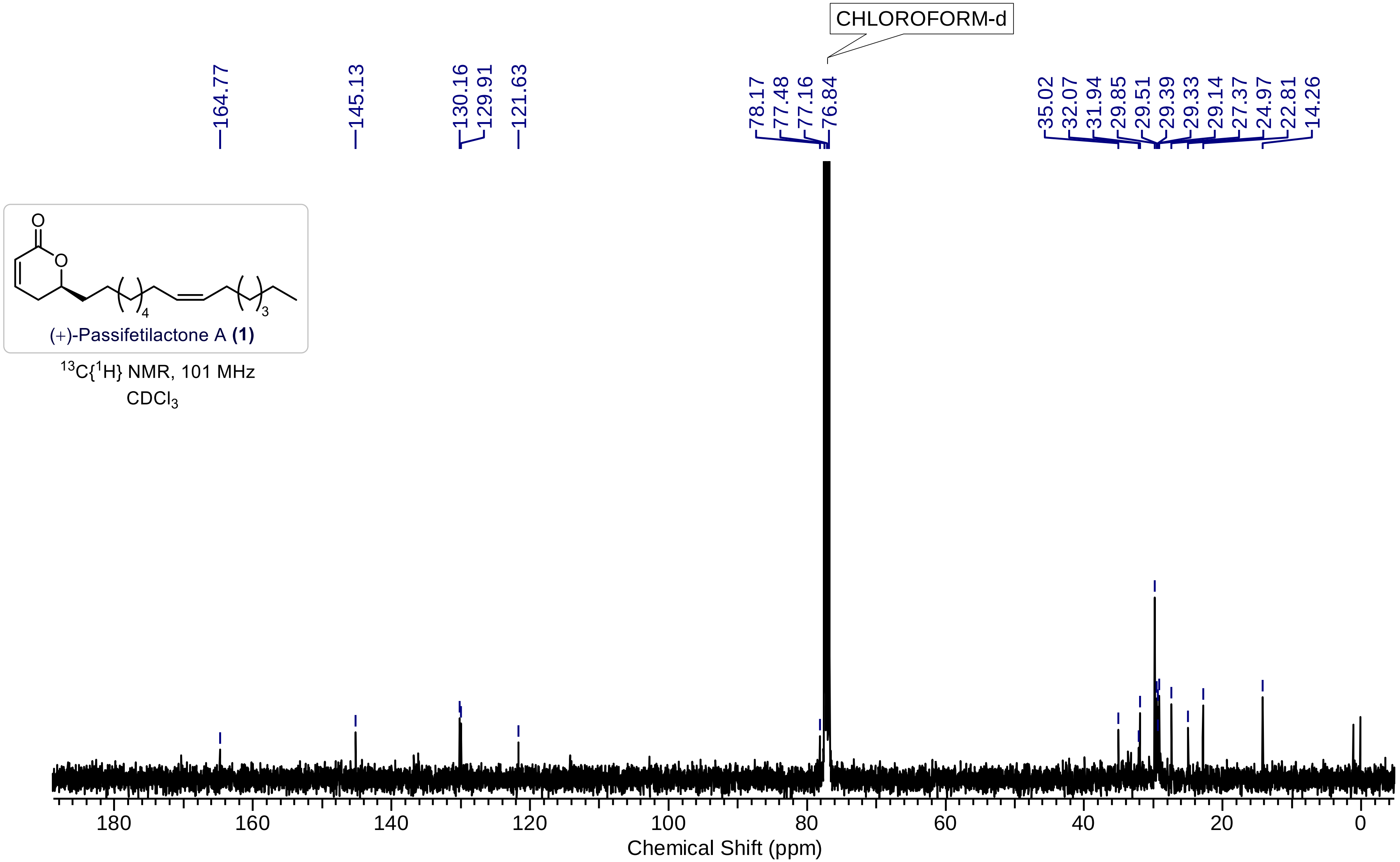

THE END
